# Targeting the hERG1/β1 integrin complex in lipid rafts potentiates statins anti-cancer activity in pancreatic cancer

**DOI:** 10.1038/s41420-025-02321-2

**Published:** 2025-02-03

**Authors:** Claudia Duranti, Jessica Iorio, Valeria Manganelli, Giacomo Bagni, Rossella Colasurdo, Tiziano Lottini, Michele Martinelli, Chiara Capitani, Giulia Boso, Franco Nicolas D’Alessandro, Maurizio Sorice, Andrea Becchetti, Roberta Misasi, Tina Garofalo, Annarosa Arcangeli

**Affiliations:** 1 Department of Experimental and Clinical Medicine, Section of Internal Medicine, University of Florence, Florence, Italy; 2 Department of Experimental Medicine, “Sapienza” University, Rome, Italy; 3 Department of Medical Biotechnologies, University of Siena, Siena, Italy; 4 Department of Biotechnology and Biosciences, University of Milano Bicocca, Milan, Italy

**Keywords:** Target identification, Pancreatic cancer

## Abstract

Plasma membrane macromolecular complexes function as signaling hubs that regulate cell behavior, which is particularly relevant in cancer. Our study provides evidence that the complex formed by the hERG1 potassium channel and the β1 subunit of integrin receptors preferentially localizes in Lipid Rafts (LRs) in Pancreatic Ductal Adenocarcinoma (PDAC) cell lines and primary samples. The complex recruits the p85 subunit of phosphatidyl-inositol-3-kinase (PI3K), activating phosphoinositide metabolism and triggering an intracellular signaling pathway centered on Akt. This pathway ultimately affects cancer cell proliferation through cyclins and p21, and cell migration through the small GTPase Rac-1 and f-actin organization. The hERG1/β1 integrin complex in LRs can be dissociated and the downstream signaling pathway can be inhibited by either disrupting LRs through methyl-beta-cyclodextrin (MβCD) or inhibiting cholesterol synthesis by statins. Treatment with a single chain bispecific antibody—scDb-hERG1-β1—specifically targeting the complex significantly potentiates the effects of both MβCD and statins on intracellular signaling. Consequently, these treatments decrease PDAC cell proliferation and motility in vitro. From a pharmacological perspective, different statins produce anti-neoplastic effects in synergy with scDb-hERG1-β1. Such combination also enhances tumor sensitivity to chemotherapeutic drugs, such as gemcitabine and oxaliplatin. The efficacy of these combination treatments depends on the amount of the hERG1/β1 integrin complex present on the plasma membrane of cancer cells. Finally, the combined treatment with statins and scDb-hERG1-β1 significantly reduces tumor growth and improves survival in vivo, in a preclinical mouse model. These results suggest that the combination of scDb-hERG1-β1 and statins represent a potential novel strategy for treating PDAC patients.

## Introduction

Local intracellular signals are increasingly recognized to be frequently triggered by the formation of macromolecular complexes on the plasma membrane which recruit cytoskeletal elements and multiple signaling molecules [1]. Such complexes are often centered on integrin receptors and thus constitute plasma membrane hubs capable of modulating signal transduction according to the features of the mechano-environment [2]. Integrin-centered macromolecular complexes often comprise growth factor receptors [3] as well as ion channels and transporters [4] and thereby regulate signaling pathways whose derangement is critical in the pathophysiology of cancer, such as those implicated in cell proliferation, survival, motility and invasiveness [5].

Lipid rafts (LRs) are major membrane organizers of spatially restricted cellular signals [6]. LRs are highly dynamic molecular assemblages, enriched in cholesterol, sphingolipids, gangliosides (peculiar is the presence of GM1) and proteins, such as caveolin-1 and flotillin-1 [7]. Such molecular composition determines the high lateral fluidity and the constant assembly/disassembly dynamics of LRs [8]. The “Lipid Raft hypothesis” [9] argues that the cell membrane organization in such microdomains is essential to ensure proper modulation of cellular functions [10] and especially of the integrin-mediated cell interaction with the extracellular matrix (ECM) [11]. For instance, LRs concentrate specific glycosylphosphatidylinositol (GPI)-linked and other signaling molecules in a spatially restricted microdomain. In general, the spatial compartmentalization of biochemical pathway components defines the specificity and enhances the efficiency of signal transduction [12]. Hence, LRs are emerging as pivotal membrane hubs to modulate intracellular pathways [13–16] that are particularly relevant in cancer [17], and may represent putative targets for anticancer drug development [18]. Activated integrins are specifically translocated into these microdomains [19], where they can interact with other molecules to regulate the integrin bi-directional signaling [20]. Integrin localization in LRs has a clear meaning in the cancer context [21], as it directs the localization and local activity of potent drivers of tumor progression, such as the small GTPase Rac1 [10, 22, 23].

LRs can also affect the function of ion channels [24] such as the *Shaker*-like [25], and those encoded by the *ether-à-go-go gene 1* (EAG1 [26]) and the *human ether-à-go-go-related gene 1* (hERG1) [27]. In brief, (i) specific lipids can directly affect ion channel functionality, e.g., in voltage-dependent K^+^ channels (K_V_) [27–30], or (ii) LRs concentrate protein kinases that modulate K_V_ activity, such as the Src kinase [24], often through the intervention of caveolin-1 [31]. The functional interaction between ion channels and the lipid component of the plasma membrane may offer a novel perspective in the comprehension of the pathophysiology of cancer [32, 33], where ion channel de-regulation represents one of the hallmarks [34]. In this context, it is worth noting that ion channels directly interact with integrins within macromolecular plasma membrane complexes [4, 35–38]. A wide evidence is offered by hERG1 [39], which is often aberrantly expressed in cancer cells [40], in which it regulates the cell responses to ECM-dependent cell adhesion [38]. In tumors, hERG1 forms a macromolecular complex with the β1 subunit of integrin receptors, after integrin engagement by activation of the non-receptor *Guanine nucleotide Exchange Factor* (GEF) Girdin and Gαi3. When complexed with integrins, hERG1 preferentially resides in the closed conformation and is protected by the RAB5-mediated endocytic pathway [41].

Based on these premises, the present work aims to determine whether the hERG1/β1 integrin complex localizes in LRs in cancer cells, from which it can trigger cancer-relevant signals, and whether such localization of the complex can be exploited for therapeutic purposes. We addressed these aims in Pancreatic Ductal Adeno Carcinoma (PDAC) which still represents an unmet medical need [42] where novel therapeutic interventions are expected.

## Results

### Localization of the hERG1/β1 Integrin complex in lipid rafts in PDAC

**Fig. 1:**
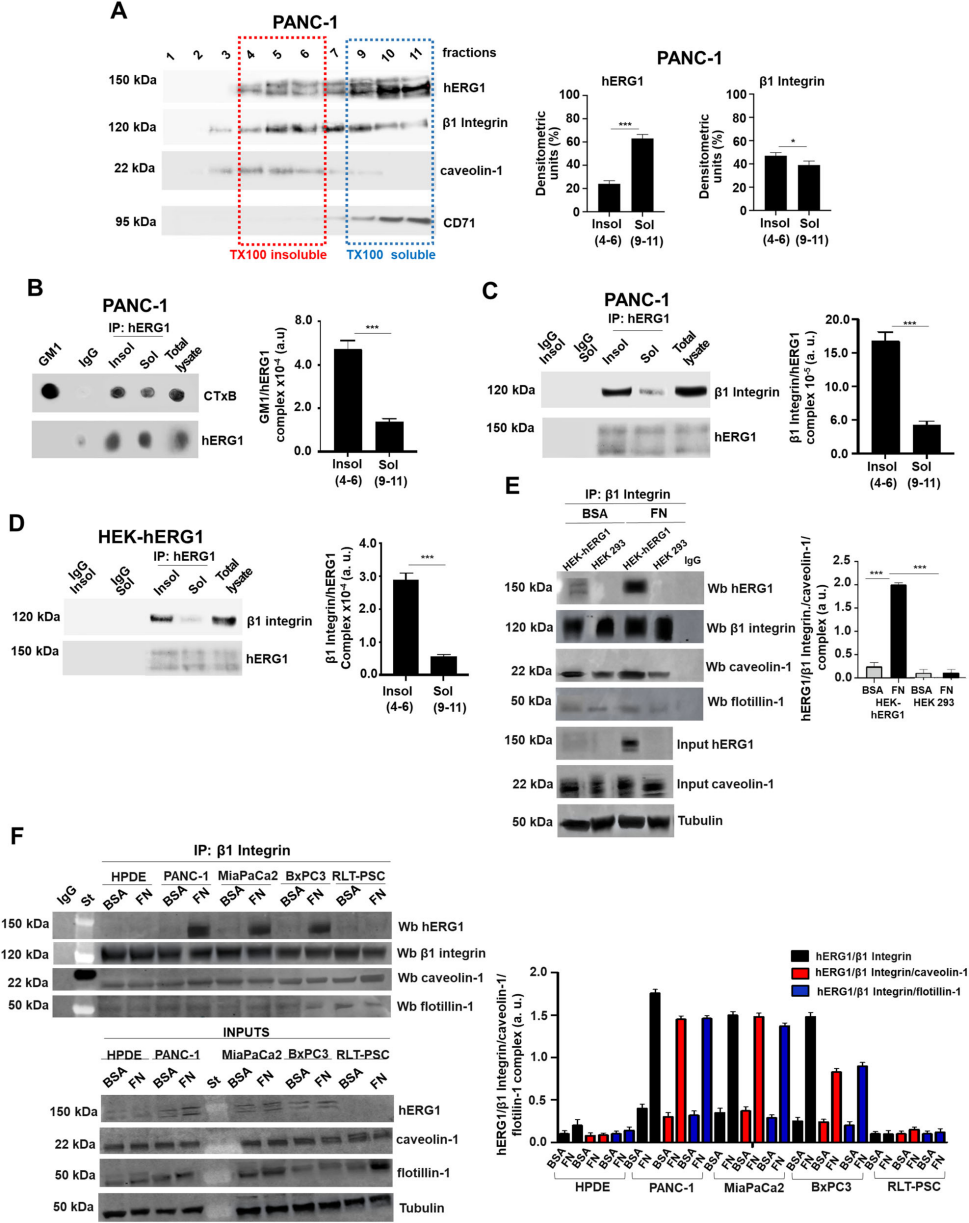
Localization of hERG1 and of the hERG1/β1 integrin complex in lipid rafts in PDAC cells after cell adhesion on Fibronectin. **A** Representative membrane of lipid rafts localization of hERG1/β1 integrin complex in PANC-1 cells. As a control, we also analyzed the distribution of two marker proteins, one of which (caveolin-1) is highly enriched in the raft fractions (4–6) and one (transferrin receptor/CD71) is mainly distributed in the Triton-soluble fractions (9–11). The panels on the right show the densitometric analysis of the fractions of the sucrose gradient. Bars indicate the percentage distribution across the gel of raft fractions 4–6 (Triton X-100 insoluble fractions) and non- rafts fractions 9–11 (Triton X-100 soluble fractions), as detected by scanning densitometric analysis; quantitation of each enriched protein was normalized over the corresponding marker, i.e caveolin-1 for raft fractions and transferrin receptor/CD71 for non-raft fractions. The density of each band in the same gel was analyzed, values were totaled, and then the percent distribution across the gel was detected and reported in the bar graphs on the right as densitometric units (%) for Triton X-100-insoluble fractions (4–6) and Triton X-100-soluble fractions (9–11). **B** Representative membrane of Triton X-100-insoluble fractions (4–6) and Triton X-100-soluble fractions (9–11) of PANC-1 cells immunoprecipitated with anti-hERG1 mAb and incubated with Cholera Toxin B Subunit Peroxidase to detect GM1 (left panel) and corresponding densiometric quantification of GM1 normalized over hERG1 immunoprecipitates(right panel). Data are representative of three independent experiments (*n* = 3). a.u.= arbitrary units. **C** Representative membrane of Triton X-100-insoluble fractions (4–6) and Triton X-100-soluble fractions (9–11) of PANC-1 cells, immunoprecipitated with mAb hERG1 and incubated with anti-Integrin β1 pAb (RM12 Ab) (left panel) and corresponding densiometric quantification of β1 Integrin over hERG1 immunoprecipitates (right panel). Data are representative of three independent experiments (*n* = 3). a.u.= arbitrary units. **D** Representative membrane of Triton X-100-insoluble fractions (4–6) and Triton X-100-soluble fractions (9–11) of HEK293-hERG1 cells, immunoprecipitated with mAb hERG1 and incubated with anti-Integrin β1 pAb (RM12 Ab) (left panel) and densiometric quantification β1 Integrin over hERG1 immunoprecipitates (right panel). Data are representative of three independent experiments (*n* = 3). a.u. = arbitrary units. **E** Representative membrane of co-IPs between β1 integrin, hERG1, caveolin-1 and flotillin-1 in HEK-hERG1 cells after cell seeding on FN for 90 min (left panel). Total cell proteins were immunoprecipitated with anti-β1 integrin mAb (TS2/16). An IgG isotypic control was employed too. Cells were seeded on BSA or FN coated dishes for 90 min. HEK 293 were used as control. Right panel: densitometric analysis. Protein lysates used for IP quantification, indicated as “inputs”, are reported in figure; protein lysates not used for IP quantification, indicated as “inputs”, are reported in Supplementary Fig. 1 (panel A). Data are representative of three independent experiments (*n* = 3). a.u. = arbitrary units. **F** Co-IP between hERG1 and β1 integrin, Co-IP between hERG1, β1 integrin, caveolin-1 and Co-IP between hERG1, β1 integrin, flotillin in normal and PDAC cells, following 90 min adhesion onto BSA or FN. Protein lysates used for IP quantification, indicated as “inputs”, are reported in figure; protein lysates not used for IP quantification, indicated as “inputs”, are reported in Supplementary Fig. 1 (panel B). Total cell proteins were immunoprecipitated with anti-β1 integrin mAb (TS2/16). An IgG isotypic control was employed. Top panel: representative WB of the co-IP; Bottom panel: densitometric analysis. Total lysates indicated as “inputs” are reported in figure. Data are representative of three independent experiments (*n* = 3). a.u. = arbitrary units. All data are presented as mean values ± s.e.m. Co-IP between hERG1 and β1 integrin: BSA vs FN: **P < 0.01: PANC-1, MiaPaca2, BxPC3; ***P < 0.001: HPDE FN vs PANC-1 FN, MiaPaca2 FN; **P < 0.01: HPDE FN vs BxPC3 FN; ***P < 0.001: RLT-PSC FN vs PANC-1 FN, MiaPaca2 FN; **P < 0.01: RLT-PSC FN FN vs BxPC3 FN. Co-IP between hERG1, β1 integrin, caveolin-1: BSA vs FN: **P < 0.01: PANC-1, MiaPaca2, BxPC3; ***P < 0.001: HPDE FN vs PANC-1 FN, MiaPaca2 FN; **P < 0.01: HPDE FN vs BxPC3 FN; ***P < 0.001: RLT-PSC FN vs PANC-1 FN, MiaPaca2 FN; **P < 0.01: RLT-PSC FN FN vs BxPC3 FN. Co-IP between hERG1, β1 integrin, flotillin: **P < 0.01: PANC-1, MiaPaca2, BxPC3; ***P < 0.001: HPDE FN vs PANC-1 FN, MiaPaca2 FN; **P < 0.01: HPDE FN vs BxPC3 FN; ***P < 0.001: RLT-PSC FN vs PANC-1 FN, MiaPaca2 FN; **P < 0.01: RLT-PSC FN FN vs BxPC3 FN. **P < 0.01, and ***P < 0.001. All data are presented as mean values ± s.e.m. *P < 0.05 and ***P < 0.001 (one-way ANOVA). Insol insoluble fraction, Sol soluble fraction, Input total lysate, BSA bovine serum albumin, FN Fibronectin, St standard.

To determine whether the hERG1/β1 integrin complex localizes in LRs in PDAC cells, we first analyzed the distribution of the two separate proteins, hERG1 and the β1 integrin in a discontinuous sucrose gradient, collecting the whole set of fractions and analyzing the proteins by Western blot (WB). PANC-1 cells, cultured in “standard conditions” (see “Materials and methods”) were used as a cellular model of PDAC. hERG1 was detectable mainly in Triton X-100 (TX100)-soluble fractions 9–11 (roughly 60%) and at a lower extent (roughly 20%) in the TX100-insoluble fractions 4–6 which correspond to LR fractions. The β1 integrin subunit was present both in TX100-insoluble and -soluble fractions, with a slightly higher presence in the former, i.e., in LRs. Caveolin-1, taken as a control marker of LRs [7], was almost restricted in detergent-insoluble fractions 4–6, as expected (Fig. 1A and the related densitometric analysis in the bar graph in the right). The hERG1 protein present in the TX100-insoluble fractions turned out to be directly bound to the lipid components of LRs, in particular to the GM1 ganglioside [7]. This is evident in the dot-blot performed on hERG1 immunoprecipitates (IPs) decorated with the cholera toxin subunit B (CTxB), which specifically labels the GM1 ganglioside [43] (Fig. 1B, and the densitometric analysis in the panel on the right). In the same IPs we verified the presence of the hERG1/β1 integrin complex decorating the blots with the anti-β1 polyclonal antibody (β1 integrin-pAb) [41]. An evident band was present almost exclusively in the hERG1 IPs from TX100-insoluble fractions, although the channel was also present to lower extent in the IPs from soluble fractions (Fig. 1C, and the densitometric analysis in the panel on the right).

Since the formation of the hERG1/β1 integrin complex is triggered by β1 integrin activation e.g., by cell adhesion to Fibronectin (FN) for 90 min (T_90_) [36, 41], we verified that the complex indeed localizes in LRs after integrin stimulation. To this purpose, we used HEK293 cells stably transfected with the hERG1 encoding gene (HEK-hERG1 cells) seeded on FN at T_90_, as a model. IPs were performed on both TX100-insoluble and TX100-soluble fractions and the WBs were decorated with the β1 integrin-pAb. Results clearly show that β1 integrins are selectively present in the hERG1 IPs from insoluble fractions (Fig. 1D and related densitometric analysis in the bar graph on the right). This indicates that the hERG1/β1 integrin complex preferentially localizes in LRs after integrin stimulation. To confirm these data we performed IPs with the β1 integrin mAb on proteins extracted from HEK-hERG1 cells seeded on either BSA (where integrins are not activated) or FN. Blots were decorated with the hERG1-polyAb as well as with antibodies directed to caveolin-1 and flotillin-1, as markers of LRs [44, 45]. hERG1, caveolin-1 and flotillin-1 were present only in the IPs obtained from HEK-hERG1 cells at T_90_ on FN, but not in HEK-hERG1 cells seeded on BSA nor in WT HEK 293 cells which do not express the channel (Fig. 1E and related densitometric analysis in the bar graph on the right). We then determined whether the hERG1/β1 integrin complex was present in LRs in different PDAC cells and whether this localization was triggered by integrin stimulation. hERG1 was present in the IPs performed with the β1 integrin mAb in PDAC cells (i.e., PANC-1, MiaPaCa2, and BxPC3) seeded on FN for 90 min, but not in those from normal Human Pancreatic Ductal Epithelial (HPDE) cells or in human pancreatic stellate cells RLT-PSC (Fig. 1F). All the β1 integrin IPs comprised caveolin-1 and flotillin-1, independently on the presence of hERG1 (Fig. 1F and relative densitometric analysis in the panel on the right). Inputs of β1 integrin relative to Fig. 1E, F are in Supplementary Fig. 1A, B.

**Fig. 2:**
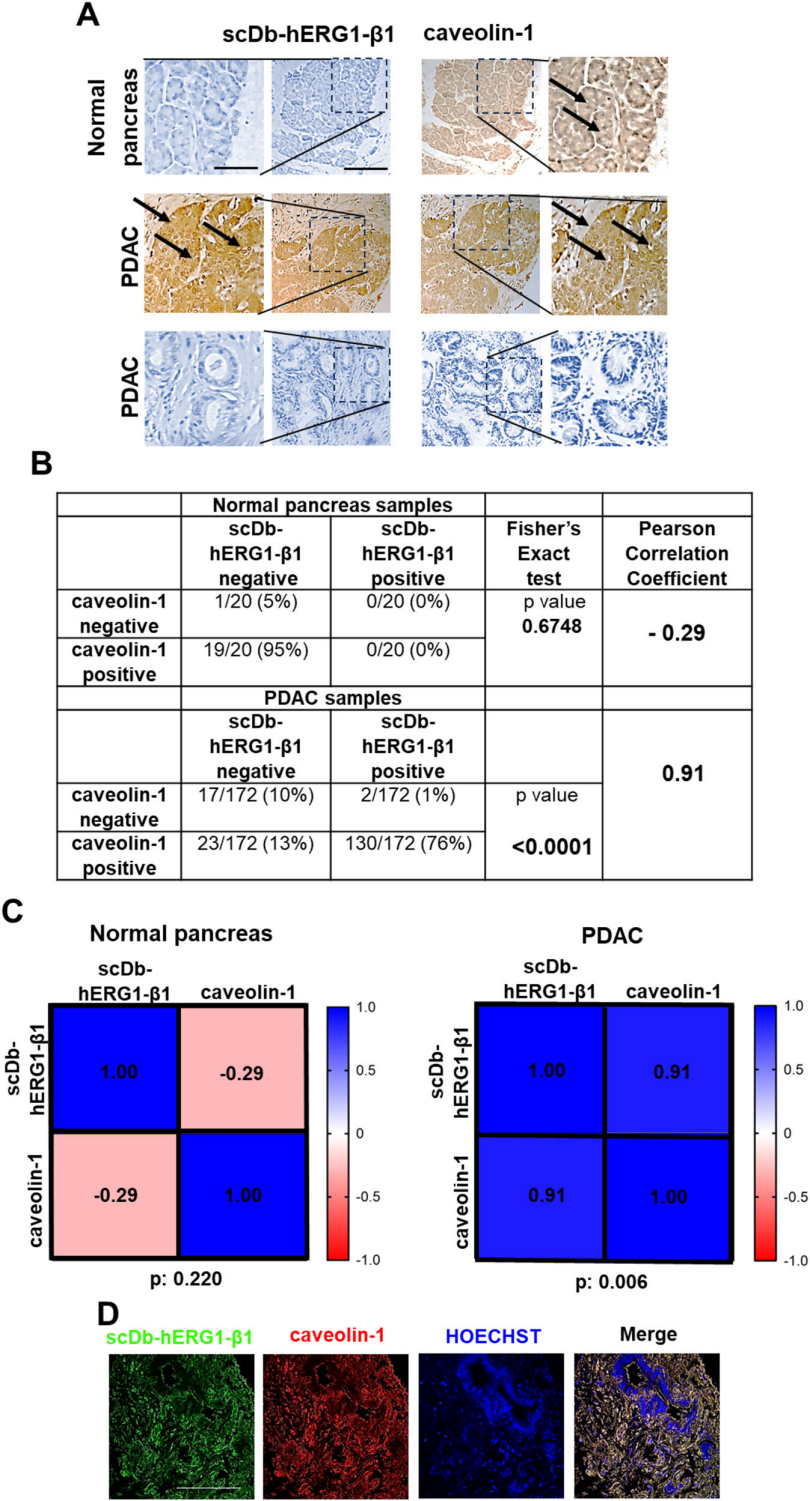
Localization of the hERG1/β1 integrin complex in lipid rafts in human primary PDAC samples. **A** Representative IHC images of scDb-hERG1-β1 and caveolin-1 staining in normal sample (top panel) and PDAC samples (bottom panels). The normal sample is positive for caveolin-1 and negative for scDb-hERG1-β1. The PDAC sample in the middle panel is positive for scDb-hERG1-β1 and caveolin-1. The PDAC sample in the bottom panel is negative for scDb-hERG1-β1 and caveolin-1. Scale bar: 100 μm. **B** Expression of hERG1-β1 complex, caveolin-1 in normal pancreas and PDAC samples. The number of negative and positive (and the corresponding percentage) samples for the two biomarkers are reported. The *p* values of Fisher’s Exact test and Pearson Correlation Coefficient are reported. **C** Heat map and Pearson correlation coefficient between scDb-hERG1-β1 and caveolin-1 in normal samples (left panel) and in PDAC samples (right panel). **D** Representative IF images showing co-localization of hERG1-β1 integrin complex and caveolin-1 in a PDAC tissue sample. Sample was stained with scDb-hERG1-β1 alexa-488 conjugated and anti-caveolin-1 antibody revealed with Alexa-546-anti-mouse secondary antibody. From left to right, scDb-hERG1-β1-alexa488, caveolin-1, Hoechst staining and Merge images are reported (scale bar: 100 µm).

We then investigated whether the hERG1/β1 integrin complex localizes in LRs in human primary PDAC samples. To this purpose, we performed an immunohistochemistry (IHC) analysis on 172 PDAC and 20 normal pancreas samples assembled in a Tissue Macro Array (TMA). Samples were stained with the single chain bispecific antibody (scDb-hERG1-β1) which specifically recognizes the hERG1/β1 integrin complex [46, 47] as well as with anti-caveolin-1 antibody, to label LRs. Figure 2A shows representative IHC pictures of the two biomarkers both in normal pancreas and PDAC. No normal pancreas samples were positive to scDb-hERG1-β1, while 19/20 (95%) were positive to caveolin-1. PDAC samples turned out to be 77% positive to scDb-hERG1-β1 (132/172) and 89% positive to caveolin-1 (153/172). A statistically significant positive correlation emerged between caveolin-1 and scDb-hERG1-β1 in PDAC (Pearson coefficient = 0.91, *p*: 0.006) and a negative correlation emerged between caveolin-1 and scDb-hERG1-β1 in healthy samples (Pearson coefficient = −0.29, *p*: 0.22) (see the Table in Fig. 2B and the heat map in Fig. 2C). The correlation between caveolin-1 and scDb-hERG1-β1 is further stressed by the co-localization between the hERG1/β1 integrin complex and caveolin-1 in a PDAC tissue sample (Fig. 2D and Supplementary Fig. 2A; hERG1/β1/caveolin-1 Manders’ overlapping coefficient (MOC) value: 0.84 ± 0.02).

We conclude that the hERG1/β1 integrin complex preferentially localizes in LRs both in PDAC cells and PDAC primary samples.

### The hERG1/β1 integrin complex in lipid rafts controls signaling pathways centered on Akt and controlling cell motility and cell cycle

**Fig. 3:**
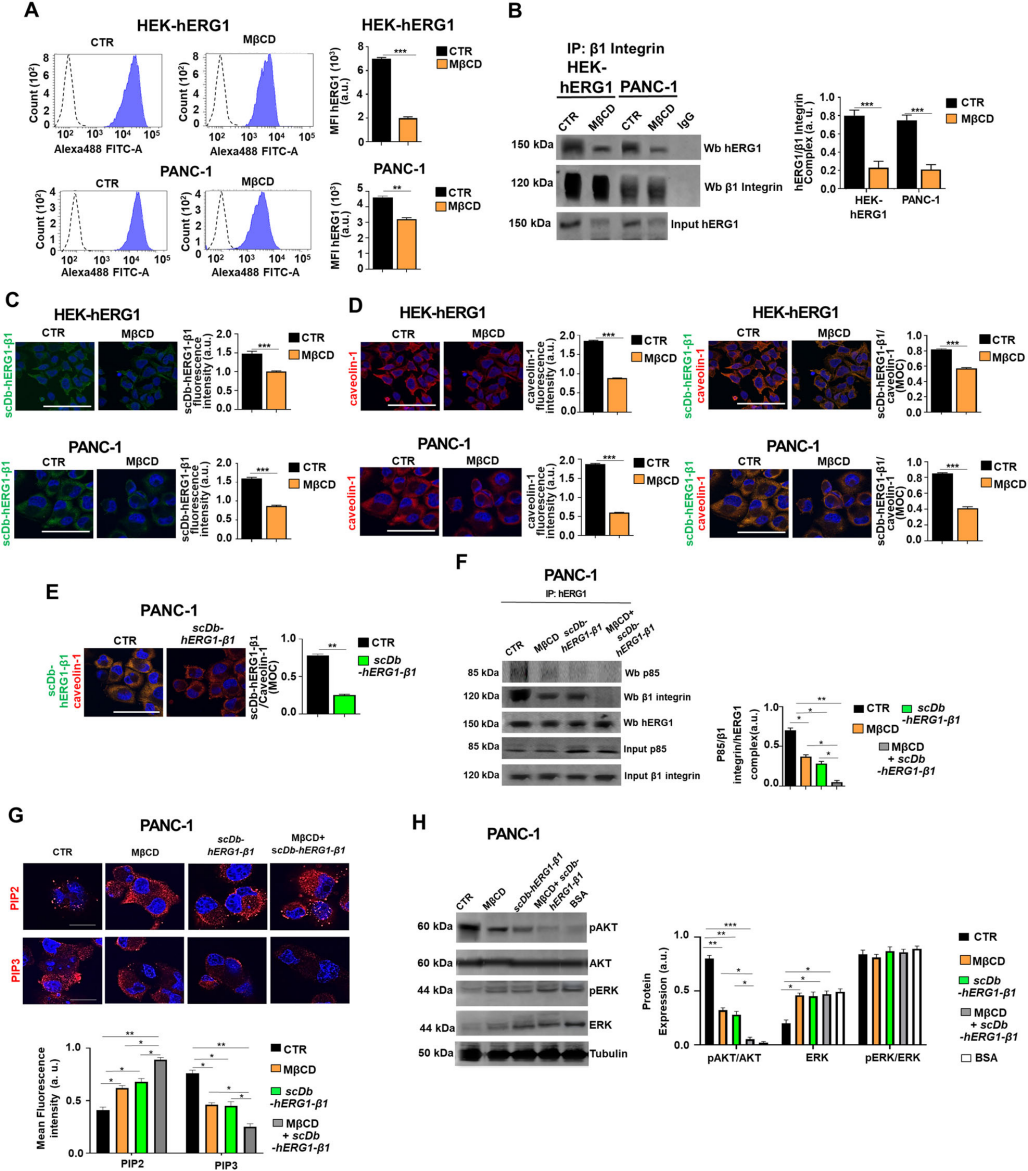
Effects of MβCD and scDb-hERG1-β1 treatment on hERG1/β1 integrin complex in PDAC cells after cell adhesion on Fibronectin. **A** Flow cytometry plots of hERG1 expression onto the plasma membrane in HEK-HERG1 and PANC-1 cells following 90 min adhesion onto FN with or without treatment with 5 mM MβCD for 20 min [48]. Values are expressed as mean fluorescence intensity of the area under the curve (MFI). Representative plots are on the left, while quantitative analyses are reported in the graphs on the right. a.u. = arbitrary units Data are mean values ± s.e.m. obtained from three independent experiments (*n* = 3). **B** Co-IP between hERG1 and β1 integrin on HEK-hERG1 and PANC-1 cells following 90 min adhesion onto FN with and without treatment with 5 mM MβCD and corresponding densitometric analysis. Total cell proteins were immunoprecipitated with anti-β1 integrin mAb (TS2/16). An IgG isotypic control was employed too. Left panel: representative WB of the co-IP; Right panel: densitometric analysis. The WBs relative to the inputs are in figure and in Supplementary Fig. 3. Data are representative of three independent experiments (*n* = 3). a.u.= arbitrary units. **C** IF performed on HEK-hERG1 and PANC-1 cells following 90 min adhesion onto FN with or without treatment with 5 mM MβCD. Representative images (scale bar: 100 μm) of scDb-hERG1-β1 staining is on the right. a.u.= arbitrary units. At least 20 cells (in 3 different fields) per condition from three independent experiments (*n* = 3) were analyzed. **D** HEK-hERG1 and PANC-1 cells following 90 min adhesion onto FN with or without treatment with 5 mM MβCD. Representative images (scale bar: 100 μm) of caveolin-1 staining and colocalization between scDb-hERG1-β1 and caveolin-1 are on the left, while quantitative analyses (fluorescent intensity and Mander’s Overlapping Coefficient, MOC) are reported in the graphs on the right. a.u.= arbitrary units. At least 20 cells (in 3 different fields) per condition from three independent experiments (*n* = 3) were analyzed. All data are presented as mean values ± s.e.m. **E** IF performed on PANC-1 cells following 90 min adhesion onto FN with or without treatment with scDb-hERG1-β1 (20 μg/ml). Representative images of colocalization between scDb-hERG1-β1 and caveolin-1 (scale bar: 100 μm) are on the left, while quantitative analyses (MOC) are reported in the graph on the right. a.u. = arbitrary units. At least 20 cells (in 3 different fields) per condition from three independent experiments (*n* = 3) were analyzed. All data are presented as mean values ± s.e.m. **F** Co-IP between hERG1 and PI3K p85 in PANC-1 cells untreated (CTR) or treated with MβCD (5 mM), scDb-hERG1-β1 (20ug/ml), and their combination, seeded on FN for 90 min. Total cell proteins were immunoprecipitated with anti-hERG1 mAb. Left panel: representative WB of the co-IP; Right panel: densitometric analysis. Total lysates indicated as “inputs” are reported in Supplementary Fig. 3. Data are representative of three independent experiments (*n* = 3). a.u. = arbitrary units. **G** IF performed on PANC-1 cells untreated (CTR) or treated with MβCD (5 mM), scDb-hERG1-β1 (20 µg/ml), and their combination, seeded on FN for 90 min. Representative images of PIP2 (top panels) and PIP3 (bottom panels) (scale bar: 50 μm) are on the top, while quantitative analyses (Mean fluorescence intensity) are reported in the graph on the bottom. At least 20 cells (in 3 different fields) per condition from three independent experiments (*n* = 3) were analyzed. All data are presented as mean values ± s.e.m. Lower magnification images are reported in Supplementary Fig. 3. **H** Representative blot (left) and densitometric analysis (right) of ERK and phospho-ERK levels in PANC-1 cells untreated (CTR) or treated with scDb-hERG1-β1 (20 µg/ml), MβCD (5 mM) and their combination, seeded on FN for 90 min and a negative control, labeled BSA. Data are presented as mean values ± s.e.m. (*n* = 3). a.u. = arbitrary units. Membranes were probed with anti-pAkt Thr308, anti-Akt Thr308, ERK1/2 (pERK1/2) (Thr202/tyr204) and anti-total ERK1/2 antibodies. CTR control, MβCD Methyl-β-cyclodextrin, MOC Mander’s Overlapping Coefficient, IP immunoprecipitation, BSA bovine serum albumin. All data are presented as mean values ± s.e.m. *P < 0.05, **P < 0.01 and ***P < 0.001 (one-way ANOVA).

**Fig. 4:**
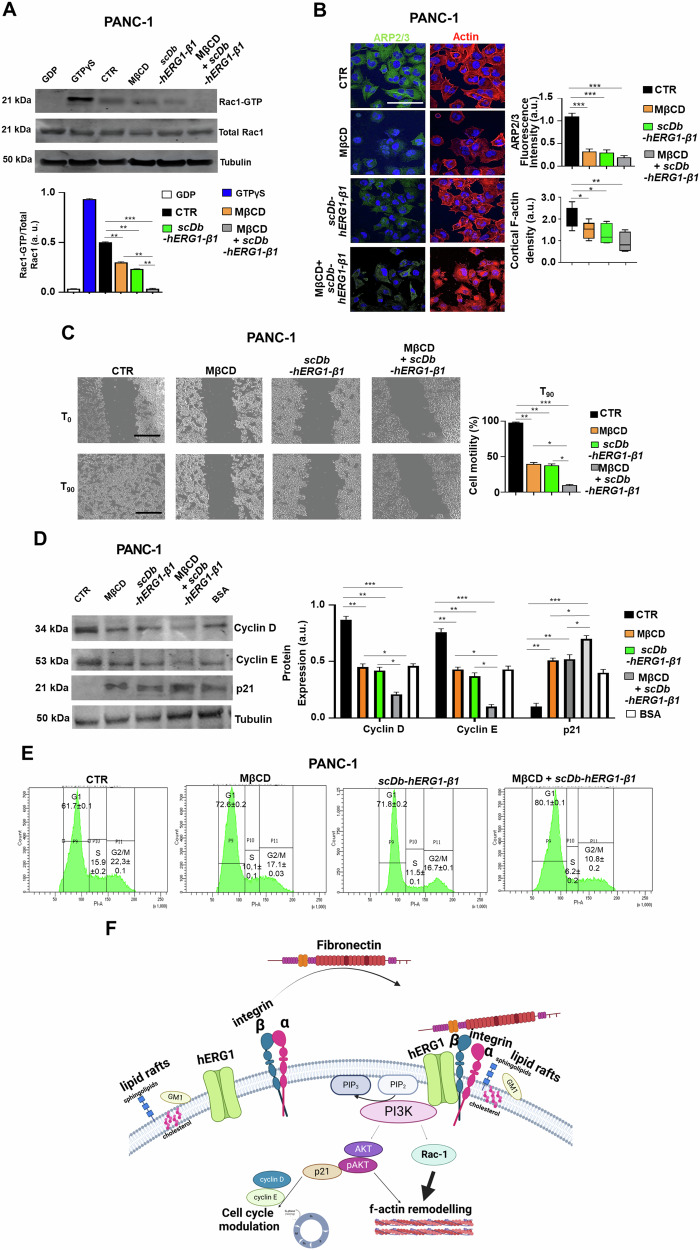
Effects of MβCD and scDb-hERG1-β1 treatment on intracellular signaling triggered by cell adhesion on Fibronectin in PDAC cells. **A** Representative blot (top) and densitometric analysis (bottom) of Rac-1 activation assay in PANC-1 cells untreated (CTR) or treated with MβCD (5 mM), scDb-hERG1-β1 (20 µg/ml) and their combination, seeded on FN for 90 min. GDP was used as negative control and GTPγS was used as positive control. Data are presented as mean values ± s.e.m. (*n* = 3). a.u. = arbitrary units. Membranes were probed with Rac-1 antibody. Inputs of total Rac-1 and tubulin are reported in the figure. **B** IF images of PANC-1 cells untreated (CTR) or treated with MβCD (5 mM), scDb-hERG1-β1 (20 µg/ml) and their combination, seeded on FN for 90 min, stained with anti-ARP2/3 antibody and Cortical F actin (left panels). Scale bar: 100 µm. At least 20 cells (in 3 different fields) per condition from three independent experiments (*n* = 3) were analyzed. Quantification graphs of ARP2/3 fluorescent intensity and cortical F-actin density were reported in the right panels. (ARP2/3 Fluorescence intensity) MβCD vs scDb-hERG1-β1: *p* = 0.02. scDb-hERG1-β1 vs MβCD+scDb-hERG1-β1: *p* = 0.01. (Cortical F-actin density) MβCD vs scDb-hERG1-β1: *p* = 0.04. scDb-hERG1-β1 vs MβCD+scDb-hERG1-β1: *p* = 0.02. **C** Lateral motility experiments onto FN were performed on PANC-1 cells treated with MβCD (5 mM), scDb-hERG1-β1 (20 μg/ml) and their combination onto FN for 90 min. Representative images are reported in the left panel. The motility is reported as graph of percentage of cell motility in the right panel. Scale bar: 100 µm. Data are presented as mean values ± s.e.m. (*n* = 3). (**D**) Representative blot (top) and densitometric analysis (bottom) of Cyclin D, Cyclin E and p21 in PANC-1 cells untreated (CTR) or treated with MβCD (5 mM), scDb-hERG1-β1 (20 µg/ml) and their combination, seeded on FN for 90 min and a negative control, labeled BSA. Data are presented as mean values ± s.e.m. (*n* = 3). a.u. = arbitrary units. Membranes were probed with anti-Cyclin D, anti-Cyclin E and anti-p21 antibodies. **E** Flow cytometry (FC) plots of cell cycle of PANC-1 cells treated with MβCD (5 mM), scDb-hERG1-β1 (20 μg/ml) and their combination for 24 h. MβCD vs CRT: *p* = 0.005 (G1); *p* = 0.042 (S); *p* = 0.041 (G2/M). scDb-hERG1-β1 vs CRT: *p* = 0.005 (G1); *p* = 0.038 (S); *p* = 0.039 (G2/M). MβCD+scDb-hERG1-β1 vs CTR: *p* = 0.0003 (G1); *p* = 0.001 (S); *p* = 0.0002 (G2/M). Data are presented as mean values ± s.e.m. (*n* = 3). **F** Integrin controlled macromolecular hubs centered on hERG1 and lipid rafts and their possible involvement in pancreatic ductal adenocarcinoma. Created with BioRender.com. *P < 0.05; **P < 0.01, and ***P < 0.001 (one-way ANOVA). GDP guanosine diphosphate, GTPγS G-protein-activating analog of guanosine triphosphate, CTR control, MβCD Methyl-β-cyclodextrin, MOC Mander’s Overlapping Coefficient, IP immunoprecipitation, BSA bovine serum albumin.

The localization of the hERG1/β1 integrin complex in LRs suggests that the signaling pathway downstream to the complex which we have described in cancer cells [41, 46] would act through LRs. We tested this hypothesis by treating the cells with methyl-β-cyclodextrin (MβCD), that drives cholesterol efflux from the membrane [48] and hence disrupts LRs. MβCD was added at 5 mM final concentration for 20 min to either HEK-hERG1 or PANC-1 cells seeded onto FN for 90 min. MβCD treatment (i) reduced hERG1 translocation to the plasma membrane, i.e., the first effect triggered by integrin stimulation [41] (Fig. 3A and related MFI values in the bar graph on the right) and (ii) decreased the formation of the hERG1/β1 integrin complex (co-IP data are in Fig. 3B and IF data are in Fig. 3C; the related densitometric analysis and MFI values are in the bar graphs on the right of each panel). MβCD treatment also reduced the localization of the hERG1/β1 integrin complex in LRs witnessed by its co-localization with caveolin-1 (Fig. 3D, right panels and related MOC values in the bar graph). All these effects were evident in PDAC cells (both PANC-1 and MiaPaCa2) and in HEK-hERG1 cells. The effects of MβCD on the formation of the hERG1/β1 integrin complex were the same as those exerted by treatment with the scDb-hERG1-β1 antibody [41]. Consistently, the antibody reduced the localization of the hERG1/β1 integrin complex in LRs, i.e., its co-localization with caveolin-1 (Fig. 3E and the related bar graphs on the right showing the MOC values). Hence, to determine whether the signaling pathway downstream to complex formation depended on its localization in LRs, we treated the cells with MβCD, the scDb-hERG1-β1 and their combination. Based on our previous results [38, 41, 49], we analyzed the signaling pathway(s) centered on Class I phosphatidyl-3-kinase (PI3K) [49]. The treatment with either MβCD or the scDb-hERG1-β1 decreased the amount of the p85 subunit of PI3K as well as of β1 integrin that co-immunoprecipitated with hERG1 (Fig. 3F and the densitometric analysis in the bar graph on the bottom) and decreased the levels of phosphatidylinositol-3,4,5-triphosphate (PIP3) while increasing phosphatidylinositol-4,5-biphosphate (PIP2) expression (Fig. 3G and related MFI values in the bar graph on the bottom). All these effects were evident in PDAC cells (both PANC-1 and MIAPaCa2) and in HEK-hERG1 cells and were improved by the combination of the two treatments (Supplementary Fig. 3). We then analyzed the effects of either treatment on the signaling pathways downstream to PI3K which are commonly triggered by integrins in cancer cells [50], focusing on Akt and the small GTPase Rac-1, which we already showed to be preferentially activated downstream to hERG1 [36]. While cell adhesion onto FN (i.e., the activation of the formation of the hERG1/β1 integrin complex [41] triggered an increase of AKT phosphorylation compared to control conditions (i.e., cell seeding onto BSA), treatments with either MβCD or scDb-hERG1-β1 prevented it (Fig. 3H and the densitometric analysis in the bar graph on the right). Interestingly, cell adhesion to FN produced a decrease in the amount of ERK, both total ERK and its phosphorylated form, compared to control (BSA) conditions (Fig. 3H and the densitometric analysis in the bar graph on the right). Accordingly, treatment with either MβCD or scDb-hERG1-β1 increased ERK phosphorylation (Fig. 3H and the densitometric analysis in the bar graph on the right). This opposite effects of the hERG1/β1 integrin complex on Akt and ERK pathway in PDAC cells agrees with what previously observed in breast cancer [38] and colorectal cancer [49] cells. We then studied the implication of Rac-1, first determining Rac-1 activity in PANC-1 cells on FN at T90 through a non-radioactive Rac1 Activation Assay Kit (see “Materials and methods” for details) [51]. Both MβCD and scDb-hERG1-β1 treatments decreased Rac-1 activity which was almost abrogated by the combined treatment (Fig. 4A and the densitometric analysis in the bar graph on the bottom). Consistently, both treatments affected f-actin organization, studied through IF experiments on PDAC cells stained with an antibody targeting the Arp2/3 complex [52] and with the CellMask^TM^ Deep Red Actin (Invitrogen) reagent to visualize f-actin. In fact, both MβCD and the scDb-hERG1-β1 decreased Arp2/3 complex staining, which almost disappeared after the combined treatment; in parallel, the f-actin stress fibers length increased while the cortical f-actin staining intensity decreased (Fig. 4B and the analyses in the bar graphs on the right, and Supplementary Fig. 4). Consistently, each treatment decreased PDAC cells motility triggered by FN at T_90_, and the combination of both produced a significantly stronger inhibitory effect (Fig. 4C and the related bar graphs on the right and Supplementary Fig. 4). Since Akt can affect cell cycle progression through a complex array of signaling molecules which converge to the modulation of cyclins [53], we then determined the effects of MβCD, the scDb-hERG1-β1 or both on signaling molecules regulating cell cycle progression on PDAC cells on FN at T_90_. Both treatments and, to a greater extent their combination, decreased the expression of both cyclin D1 and cyclin E, paralleled by an increase of p21 expression (Fig. 4D and Supplementary Fig. 4). This effect was accompanied by a mirrored increase of cells number in G1 phase and a decrease of the number of cells in S phase (Fig. 4E and Supplementary Fig. 4).

Overall, either disrupting LRs with MβCD or dissociating the hERG1/β1 integrin complex produces a similar modulation of an intracellular signaling pathway which involves PI3K, Akt and its downstream effectors: the small GTPase Rac-1 and cyclins. This pathway converges to the modulation of f-actin organization which makes PDAC cells more motile as well as to the control of cell cycle progression (Fig. 4F).

### Lowering cellular cholesterol content with statins inhibits PDAC cell proliferation and migration dissociating the hERG1/β1 integrin complex in LRs

**Fig. 5:**
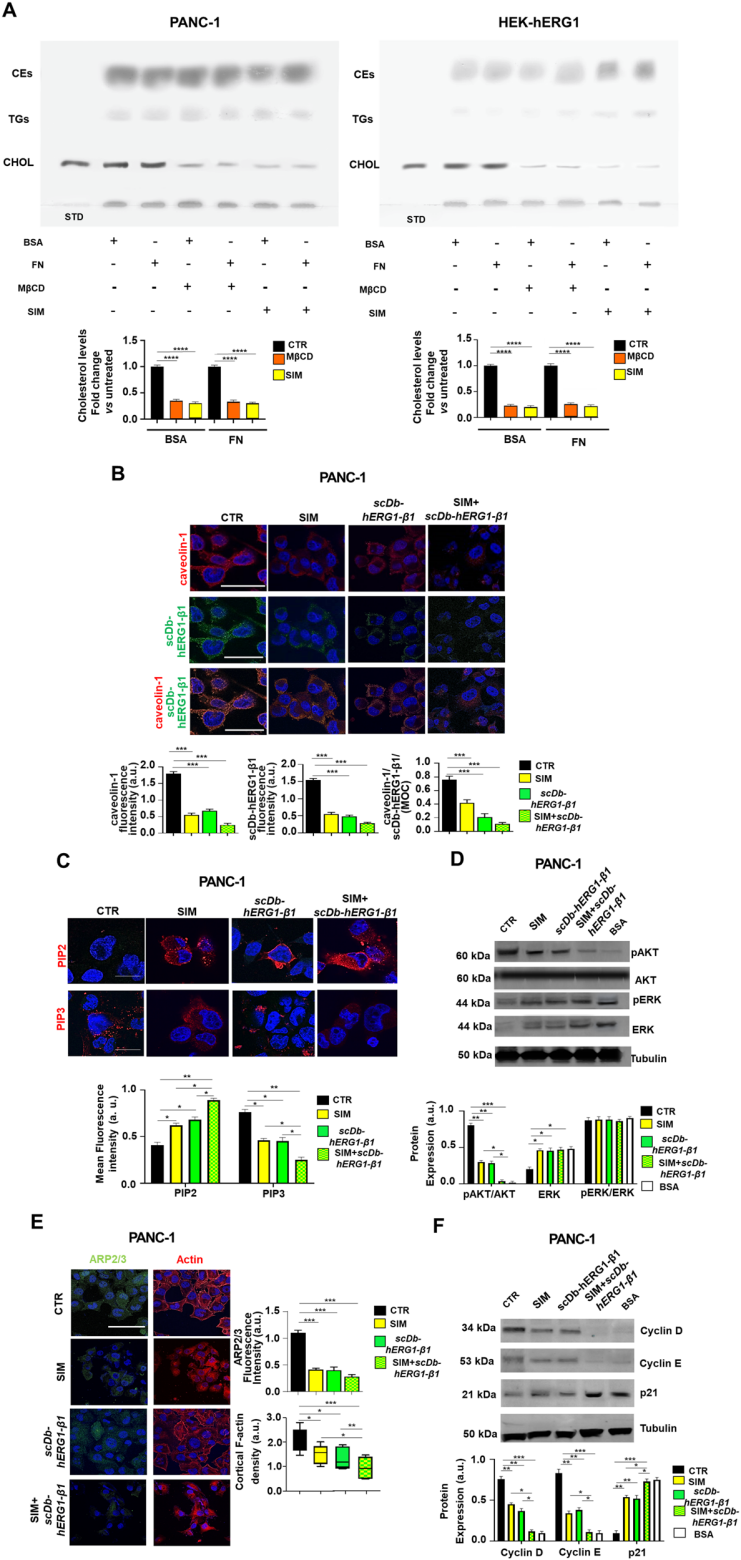
Effects of SIMVASTATIN (SIM) and of scDb-hERG1-β1 treatment on intracellular signaling triggered by cell adhesion on Fibronectin in PDAC cells. **A** PANC-1 and HEK-hERG1 cells untreated or treated with SIM or MβCD were subject to cholesterol quantification by HPTLC. Quantitative analysis of separated free cholesterol was carried out using NIH Image1.62 as software. **B** IF performed on PANC-1 cells following 90 min adhesion onto FN with or without treatment with SIM, scDb-hERG1-β1 and their combination. Representative images (scale bar: 100 μm) of caveolin-1 staining, scDb-hERG1-β1 staining and colocalization between caveolin-1 and scDb-hERG1-β1 are on the top, while quantitative analyses and MOC are reported in the graphs on the bottom. a.u.= arbitrary units. At least 20 cells (in 3 different fields) per condition from three independent experiments (*n* = 3) were analyzed. All data are presented as mean values ± s.e.m. **C** IF performed on PANC-1 cells untreated (CTR) or treated with SIM (4.7 μM), scDb-hERG1-β1 (20 µg/ml), and their combination, seeded on FN for 90 min. Representative images of PIP2 (top panels) and PIP3 (bottom panels) (scale bar: 50 μm) are on the top, while quantitative analyses (Mean fluorescence intensity) are reported in the graph on the bottom. At least 20 cells (in 3 different fields) per condition from three independent experiments (*n* = 3) were analyzed. All data are presented as mean values ± s.e.m. **D** Representative blot (top) and densitometric analysis (bottom) of phospho-Akt and phospho-ERK levels in PANC-1 cells untreated (CTR) or treated with SIM (4.7 µM), scDb-hERG1-β1 (20 µg/ml) and their combination, seeded on FN for 90 min. Data are presented as mean values ± s.e.m. (*n* = 3). a.u. = arbitrary units. Membranes were probed with anti-pAkt Thr308, anti-Akt Thr308, ERK1/2 (pERK1/2) (Thr202/tyr204) and anti-total ERK1/2 antibodies. **E** IF on PANC-1 cells stained with anti-ARP2/3 antibody and cortical F-actin (left panels) after treatment with SIM (4.7 μM), scDb-hERG1-β1 (20 µg/ml) and their combination onto FN for 90 min (scale bar: 100 μm). At least 20 cells (in 3 different fields) per condition from three independent experiments (*n* = 3) were analyzed. Quantification graphs of ARP2/3 fluorescent intensity and cortical F-actin density were reported in the right panels. Data are presented as mean values ± s.e.m. **F** Representative blot (top) and densitometric analysis (bottom) of Cyclin D, Cyclin E and p21 in PANC-1 cells untreated (CTR) or treated with scDb-hERG1-β1 (20ug/ml), SIM (4.7 µM) and their combination, seeded on FN for 90 min. Data are presented as mean values ± s.e.m. (*n* = 3). a.u. = arbitrary units. Membranes were probed with anti-Cyclin D, anti-Cyclin E and anti-p21 antibodies. *P < 0.05; **P < 0.01, and ***P < 0.001 (one-way ANOVA). CTR control, MβCD Methyl-β-cyclodextrin, SIM simvastatin, CHOL Free cholesterol, TGs triglycerides, CEs cholesterol esters, MOC Mander’s Overlapping Coefficient.

**Fig. 6:**
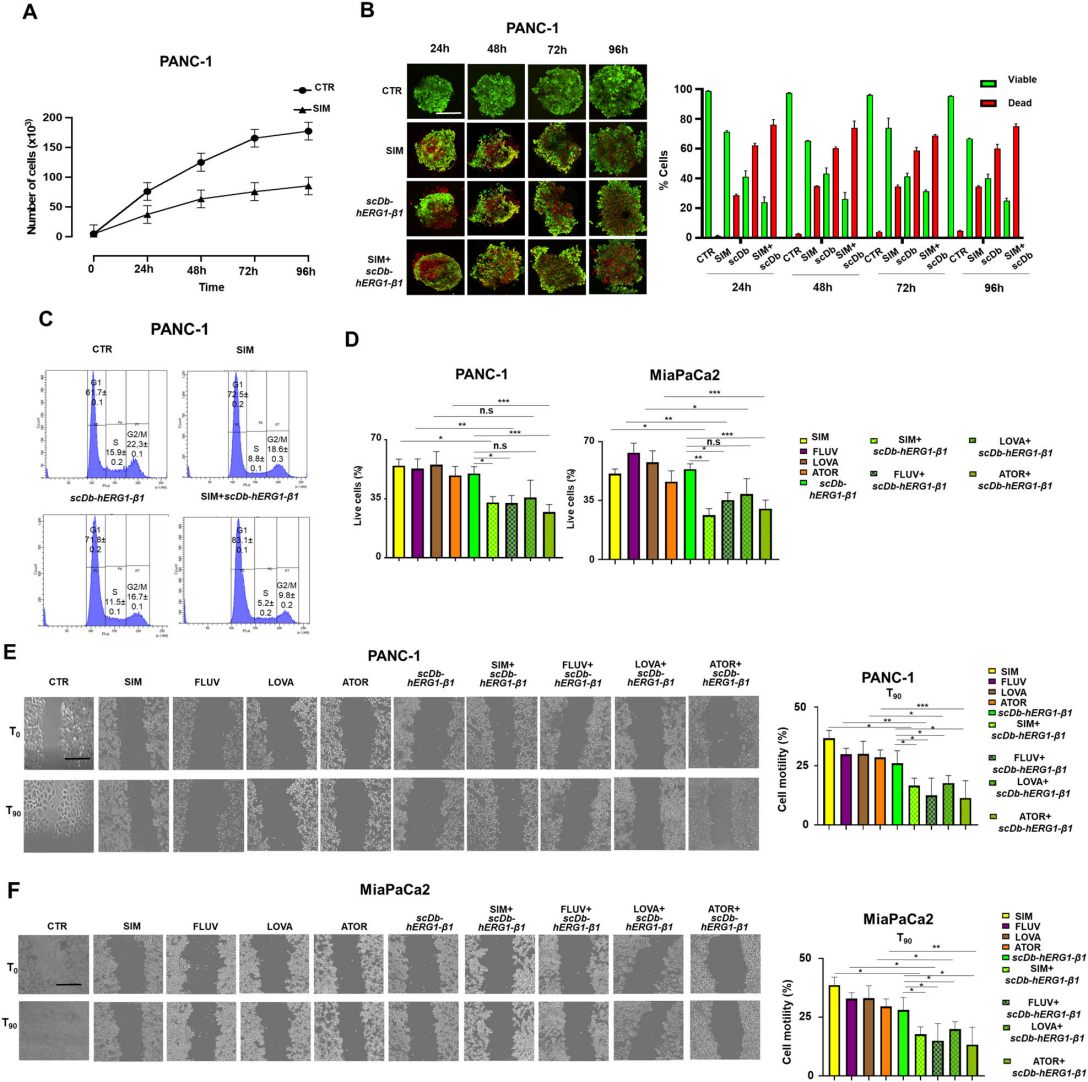
Effects of statins and scDb-hERG1-β1 treatments on PDAC cell vitality, proliferation and migration. **A** Proliferation curve of PANC-1 CTR and treated with IC_50_ of SIM for 24 h, 48 h, 72 h and 96 h. Data are presented as mean values ± s.e.m. (*n* = 3). **B** Representative Calcein/PI images of PANC-1 CTR and treated with IC_50_ of SIM, scDb-hERG1-β1 and IC_50_ of SIM + scDb-hERG1-β1 for 24 h, 48 h, 72 h and 96 h (left panel) (scale bar: 100 μm) and cell live index (%) graph (right panel). Data are presented as mean values ± s.e.m. (*n* = 3). **C** Facs plots of cell cycle of PANC-1 cells treated with with IC_50_ of SIM, scDb-hERG1-β1 and IC_50_ of SIM + scDb-hERG1-β1 for 24 h. SIM vs CRT: *p* = 0.004 (G1); *p* = 0.021 (S); *p* = 0.038 (G2/M). SIM vs scDb-hERG1-β1: *p* = 0.005 (G1); *p* = 0.038 (S); *p* = 0.039 (G2/M). SIM+ scDb-hERG1-β1 vs CRT: *p* = 0.0002 (G1); *p* = 0.001 (S); *p* = 0.0001 (G2/M). Data are presented as mean values ± s.e.m. (*n* = 3). **D** Graph of percentage live cells, PANC-1 (left panel) and MiaPaCa2 (right panel) cells, treated for 24 h with IC50 of SIM, FLUVA, LOVA, ATOR, scDb-hERG1-β1 and combination of each statin with scDb-hERG1-β1 are reported. Data are presented as mean values ± s.e.m. (*n* = 3). **E** Lateral motility experiments onto FN were performed on PANC-1 cells treated with SIM, FLUVA, LOVA, ATOR, scDb-hERG1-β1 (at IC50 values for 90 min) and combinations of each statin and scDb-hERG1-β1 (at IC50 values for 90 min). Representative images are reported in the left panel. The motility is reported as graph of percentage of cell motility in the right panel. Scale bar: 100 µm. Data are presented as mean values ± s.e.m. (*n* = 3). **F** Lateral motility experiments onto FN were performed on MiaPaCa2 cells treated with SIM, FLUVA, LOVA, ATOR, scDb-hERG1-β1 (at IC50 values for 90 min) and combinations of each statin and scDb-hERG1-β1 (at IC50 values for 90 min). Representative images are reported in the left panel. The motility is reported as graph of percentage of cell motility in the right panel. Scale bar: 100 µm. Data are presented as mean values ± s.e.m. (*n* = 3). *P < 0.05, **P < 0.01 and ***P < 0.001 (one-way ANOVA). CTR control, SIM Simvastatin, FLUVA Fluvastatin, LOVA Lovastatin, ATOR Atorvastatin.

We then argued that decreasing intracellular cholesterol content could exert the same effects obtained when using MβCD on PDAC cells. To this purpose we studied the effects of statins, that act as HMG-CoA reductase inhibitors, consequently leading to decreased cholesterol concentrations [54]. We first determined cholesterol content in PANC-1 and HEK-hERG1 cells seeded onto BSA or FN, untreated or treated with Simvastatin (SIM), as a model statin already used in PDAC [55–57] or MβCD. The HPTLC, shown in Fig. 5A, shows the effects of both SIM and MβCD on different lipids of either PANC-1 or HEK-hERG1 cells. Interestingly for the purpose of the present study, both SIM and MβCD reduced the total free cholesterol content in both PANC-1 and HEK-hERG1 cells (see the lower bands indicated by CHOL and the densitometric scanning analysis reported on the bottom of Fig. 5A). Consistently, SIM reduced the expression of caveolin-1 in PANC-1 cells (Fig. 5B, top panels), the formation of the hERG1/β1 integrin complex (Fig. 5B, middle panels) and its co-localization with caveolin-1 (Fig. 5B, bottom panels; see also the corresponding values of MFI and MOC in the bar graphs on the right). SIM also affected the signaling pathway detailed in Figs. 3 and 4, since it reduced (i) the levels of PIP3 (while increasing PIP2 expression) (Fig. 5C); (ii) Akt phosphorylation (Fig. 5D); (iii) Arp 2/3 fluorescence intensity and cortical f-actin density (Fig. 5E) and (iv) the expression of cyclins (Fig. 5F). All these effects were potentiated by the combination with the scDb-hERG1-β1 (Fig. 5B–F, pictures labeled as “SIM + scDb-hERG1-β1 Treatment” and related bar graphs). We conclude that SIM disassembles LRs in PDAC cells and dissociates the hERG1/β1 integrin complex from LRs, hence decreasing the signaling pathway triggered by the complex. These results prompted us to test the effects of SIM on vitality, proliferation (in 2D and 3D) and migration of PANC-1 cells, alone and in combination with the scDb-hERG1-β1. SIM used at its IC_50_ value (i.e., 4.5 μM, see Table 1 and Supplementary Fig. 5) reduced the proliferation of PANC-1 cells cultured either in 2D (Fig. 6A) or in 3D as spheroids (Fig. 6B, top panels and bar graph labeled as B’). The antiproliferative effect of SIM could be traced back to an effect on cell cycle progression (Fig. 6C, top panels). All these effects were potentiated by the combination with the scDb-hERG1-β1 antibody (Fig. 6B, panels on the bottom and bar graph labeled as B” and Fig. 6C, bottom panels). Different statins (Fluvastatin (FLUV), Lovastatin (LOVA) and Atorvastatin (ATOR)) whose IC_50_ values are in Table 1 and Supplementary Fig. 6, reduced cell vitality after 24 h of treatment (see the yellow, purple, brown and orange squared pattern bars in Fig. 6D) and their effects were significantly higher when combined with the scDb-hERG1-β1 (Fig. 6D). The combination of scDb-hERG1-β1 with either SIM or ATOR was synergic in both PANC-1 and MiaPaCa2 cells (Table 1). Furthermore, all the statins also significantly reduced FN-induced motility in both PANC-1 (Fig. 6E) and MiaPaCa2 cells (Fig. 6F), and these effects were significantly potentiated by the combination with scDb-hERG1-β1 (Fig. 6E, F).

**Table 1:** IC_50_ values and combination index of SIM, FLUV, LOVA, ATOR and scDb-hERG1-β1 in PANC-1 and MiaPaCa2 cells.

PANC-1	IC_50_ (μM)	Combination Index with scDb-hERG1-β1 IC_50_ (20.8 μM)	Effect
SIM	4.47 ± 0.48	0.27 ± 0.01	Synergy
FLUV	2.47 ± 1.06		
LOVA	2.79 ± 0.84		
ATOR	1.81 ± 1.12	0.61 ± 0.02	Synergy
MiaPaCa2			
SIM	4.16 ± 1.02	0.37 ± 0.03	Synergy
FLUV	3.45 ± 1.05		
LOVA	2.07 ± 1.01		
ATOR	3.13 ± 1.13	0.63 ± 0.01	Synergy

IC_50_ values were determined after 24 h of treatment by the Trypan Blue exclusion test, using the Origin Software, in PANC-1 and MiaPaCa2 cells. Combination index in PANC-1 and MiaPaCa2 cells after different treatment combinations. All the drugs were used at drug concentrations indicated in the first column. Data are means ± s.e.m. of three independent experiments, each carried out in triplicate. CI values were calculated using the Calcusyn software Version 2 (Biosoft). For statistical analysis, Student’s *t*-test was applied.

### The expression of the hERG1/β1 integrin complex on PDAC cells determines statins anticancer activity, alone and in combination with chemotherapeutic drugs

**Fig. 7:**
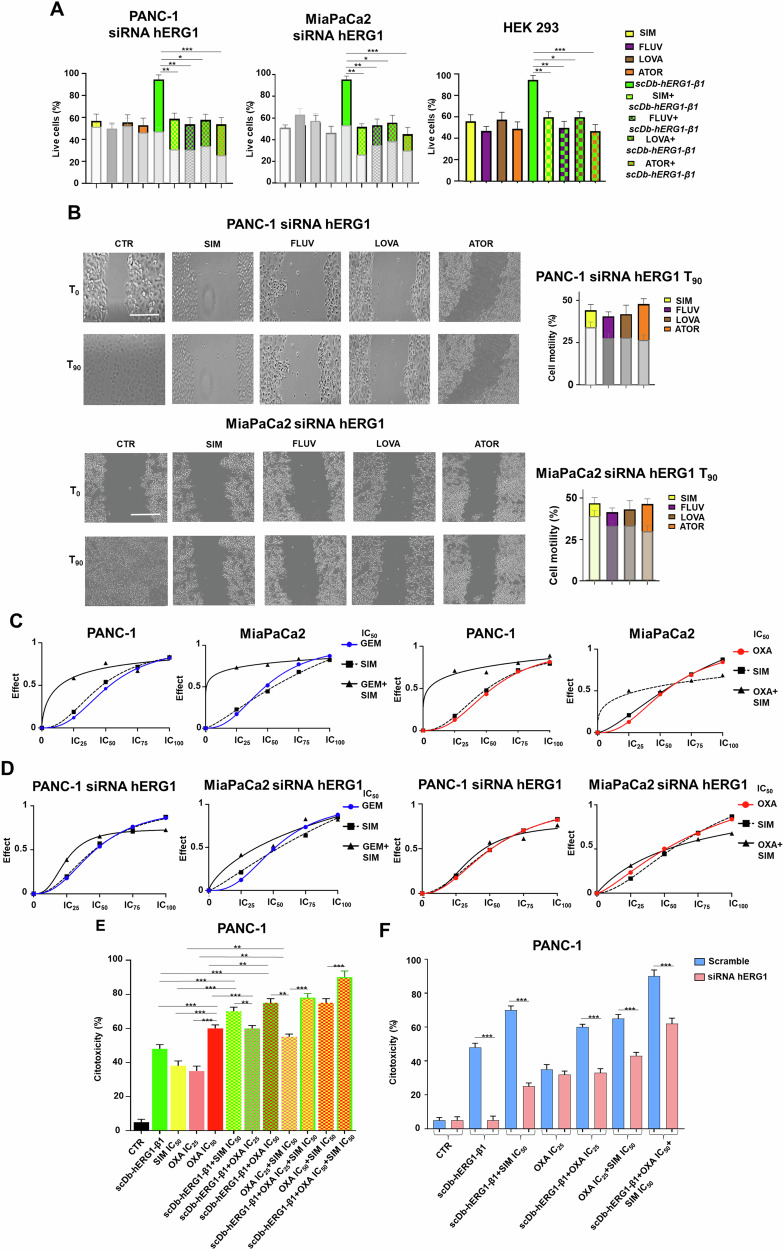
Effects of statins, gemcitabine and oxaliplatin on cell vitality and migration in PDAC cells and PDAC hERG1 silenced cells. **A** Graph of percentage live cells in hERG1 silenced PANC-1, MiaPaCa2 and HEK 293 cells treated for 24 h with IC50 of SIM, FLUVA, LOVA, ATOR, scDb-hERG1-β1 and combination of each statin with scDb-hERG1-β1 is reported. The values of no hERG1 silenced PANC-1 and MiaPaCa2 cells are reported in gray bars. Data are presented as mean values ± s.e.m. (*n* = 3). **B** Lateral motility experiments onto FN performed on hERG1 silenced PANC-1 and MiaPaCa2 cells treated with SIM, FLUVA, LOVA, ATOR (at IC50 values for 90 min). The motility is reported as graph of percentage of cell motility; the values of no hERG1 silenced PANC-1 and MiaPaCa2 cells are reported in gray bars. Scale bar: 100 µm. Data are presented as mean values ± s.e.m. (*n* = 3). **C** Combination Index curves of PANC-1 and MiaPaCa2 cells CTR and treated with statins, GEM (left panel) and OXA (right panel). We combined the IC25, IC50, IC75 and IC100 of the two chemotherapeutic drugs with the IC25, IC50, IC75 and IC100 of the four statins, deriving these concentrations from the IC50 values shown in Table 1. The Combination Index for all treatments was then calculated. Data are presented as mean values ± s.e.m. (*n* = 3). **D** Combination Index curves of PANC-1 and MiaPaCa2 hERG1 silenced cells CTR and treated with statins, GEM (left panel) and OXA (right panel). Data are presented as mean values ± s.e.m. (*n* = 3). **E** LDH assay on PANC-1 3D cells treated with SIM (IC50 value), OXA (IC50 and IC25) and scDb-hERG1-β1 (20 µg/ml) for 48 h. The effects of treatment were evaluated through the LDH assay, and the data as shown as percentage of cytotoxicity (see “Materials and methods” for details). Data are presented as mean values ± s.e.m. (*n* = 3). **F** LDH assay on hERG1 silenced PANC-1 3D cells (pink bars) and samples transfected with Lipofectamine but no siRNAs, indicated as “Scramble” (light blue bars) PANC-1 3D cells treated with (IC50 value), OXA (IC50 and IC25) and scDb-hERG1-β1 (20 µg/ml) for 48 h. Representative images of 3D cells are reported in Supplementary Fig. 7. Data are presented as mean values ± s.e.m. (*n* = 3). *P < 0.05, **P < 0.01 and ***P < 0.001 (one-way ANOVA). CTR control, IC inhibitory concentration, SIM Simvastatin, FLUVA Fluvastatin, LOVA Lovastatin, ATOR Atorvastatin, GEM gemcitabine, OXA oxaliplatin.

Given the effects of the combination of statins with the scDb-hERG1-β1, we wondered whether the expression of the complex on PDAC cells was relevant in determining statins’ effects. To this purpose, we tested either SIM or ATOR on hERG1-silenced PANC-1 and MiaPaCa2 cells (the effects of silencing are in Supplementary Fig. 7). The IC_50_ values of the two statins turned out to be significantly higher in PANC-1 and MIaPaCa2 cells silenced for hERG1 compared to cells treated with scramble siRNAs (Supplementary Fig. 7B and Table 2). Conversely, the IC_50_ values of SIM and ATOR were significantly lower in HEK-hERG1 cells compared to WT HEK-293 (Supplementary Fig. 7C and Table 2). The combination of the different statins with scDb-hERG1-β1 did not potentiate the reduction of cell vitality in hERG1-silenced cells as well as in WT HEK 293 cells (Fig. 7A). All the statins were less effective in reducing cell motility on hERG1-silenced PANC-1 and MiaPaCa2 cells compared to cells treated with scramble siRNAs (Fig. 7B).

**Table 2:** IC_50_ values of SIM and ATOR in PANC-1 and MiaPaCa2 hERG1 silenced cells, HEK293 and HEK-hERG1 cells.

PANC-1 siRNA hERG1	IC_50_ (μM)	MIaPaCa2 siRNA hERG1	IC_50_ (μM)
SIM	11.61 ± 1.12 *p*: 0.02	SIM	12.01 ± 2.14 *p*: 0.03
ATOR	12.89 ± 1.61 *p*: 0.01	ATOR	13.87 ± 1.02 *p*: 0.02
PANC-1 lipofectamine		MiaPaCa2 lipofectamine	
SIM	4.31 ± 1.13	SIM	5.76 ± 1.43
ATOR	1.85 ± 2.12	ATOR	3.77 ± 0.73
HEK 293		HEK-hERG1	
SIM	9.09 ± 0.81	SIM	3.13 ± 1.08
ATOR	14.99 ± 0.52	ATOR	2.19 ± 0.15

IC_50_ values were determined after 24 h of treatment by the Trypan Blue exclusion test, using the Origin Software in PANC-1 and MiaPaCa2 hERG1 silenced cells. Data are means ± s.e.m. of three independent experiments, each carried out in triplicate IC_50_ values were determined after 24 h of treatment by the Trypan Blue exclusion test, using the Origin Software, in PANC-1 and MiaPaCa2 hERG1 silenced cells, HEK293 and HEK-hERG1 cells.

We then tested whether statins, alone or in combination with the scDb-hERG1-β1, could increase the chemosensitivity of PDAC cells. To this purpose, we tested two drugs commonly used for PDAC treatment (Gemcitabine (GEM) and Oxaliplatin (OXA)), first calculating the IC_50_ value of OXA (Fig. 7C and Supplementary Fig. 7D) and deriving the IC_50_ value from [47] for GEM and then determining the Combination Indexes (CI) for all the treatments. Generally, the combination of the two treatments gave a stronger cytotoxic effect (Fig. 7E and Supplementary Table S1), which was synergic when considering the IC_25_ doses of the statins and for OXA. The synergistic effect of statins with either GEM or OXA was less evident in PANC-1 and MIaPaCa2 cells silenced for hERG1 (pink bars) compared to cells treated with scramble siRNAs (light blue bars, Fig. 7D, F and Supplementary Table S1). Then, we evaluated the effects of OXA (at the IC_25_ dose) in combination with SIM and scDb-hERG1-β1, both at their IC_50_ doses, on PANC-1 cells cultured in 3D as spheroids. The combined treatment produced a significantly higher cytotoxicity (Supplementary Fig. 7D and Supplementary Table S1). Even in this case the cytotoxic effect of the drug combinations was less evident in hERG1-silenced PANC-1 cells compared to cells treated with scramble siRNAs (Supplementary Fig. 7G and Supplementary Table S1).

### The treatment with the scDb-hERG1-β1 antibody enhances statins antitumor activity in vivo

**Fig. 8:**
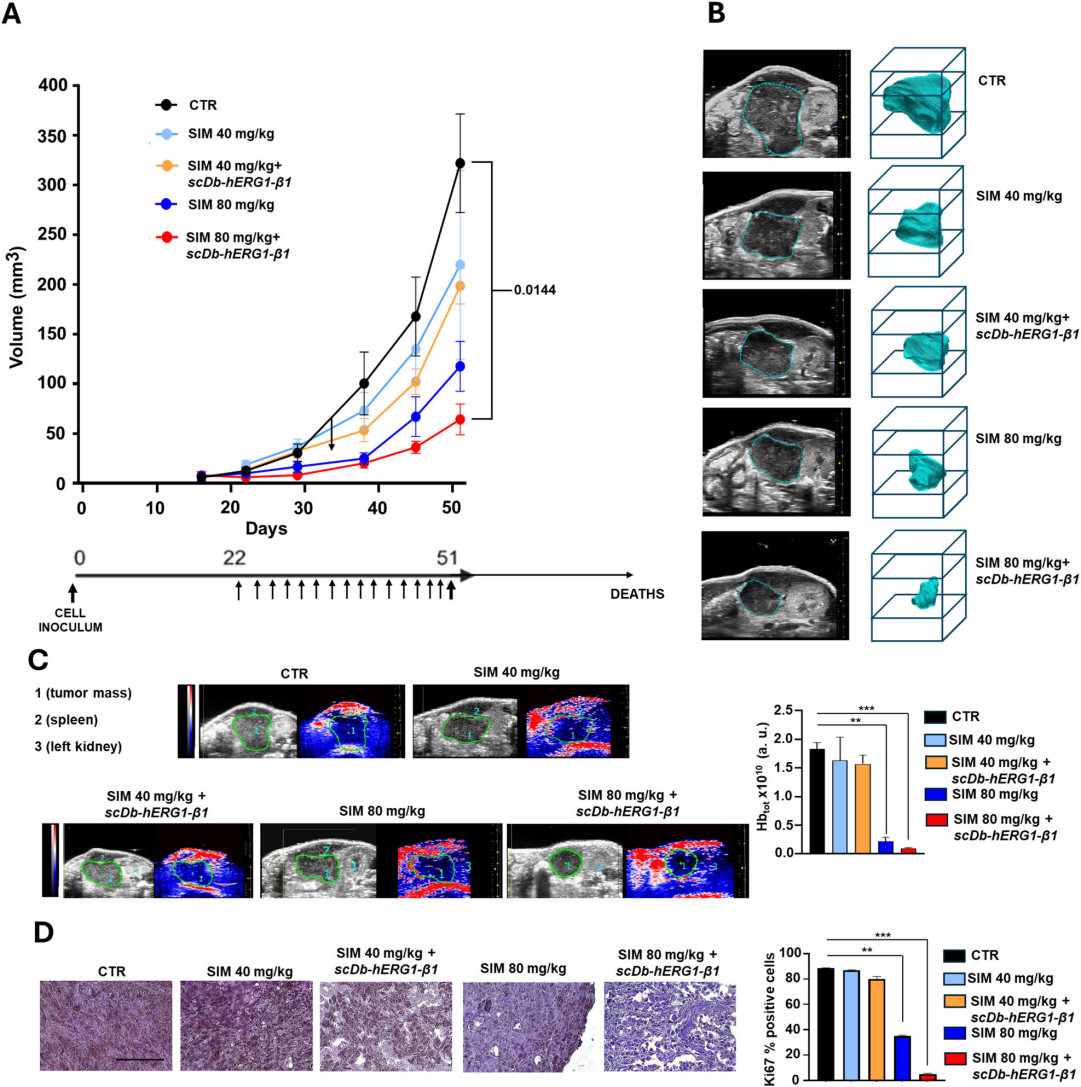
Effects of SIM and scDb-hERG1-β1 treatment in vivo in a PDAC mouse model. **A** Time course of the volumes of tumor masses of PANC-1 cells (top panel) growing in mice treated with SIM 40 mg/kg, SIM 80 mg/kg and combination with scDb-hERG1-β1 16 mg/kg. *P* Values obtained with one-way ANOVA analysis at day 51 of experiment are reported below: CTR vs. SIM 40 *p*: 0.6859; CTR vs. SIM 40+ scDb-hERG1-β1 *p*: 0.5012; CTR vs. SIM 80 *p*: 0.0415; CTR vs. SIM 80+ scDb-hERG1-β1 *p*: 0.0144; SIM 40 vs. SIM 40+ scDb-hERG1-β1 *p*: 0.9996; SIM 40 vs. SIM 80 *p*: 0.6028; SIM 40 vs. SIM 80+ scDb-hERG1-β1 *p*: 0.2637; SIM 40+ scDb-hERG1-β1 vs. SIM 80 *p*: 0.7959; SIM 40+ scDb-hERG1-β1 vs. SIM 80+ scDb-hERG1-β1 *p*: 0.4126; SIM 80 vs. SIM 80+ scDb-hERG1-β1 *p*: 0.1423. Schedule of treatment (bottom panel). **B** Representative US images of tumor masses with rendering from PANC-1 cells at day 51 of mice untreated (CTR) and treated with SIM 40 mg/kg, SIM 80 mg/kg and combination with scDb-hERG1-β1 16 mg/kg. **C** Photoacoustic images (left panels) and related bar graph of HB total levels (right panel). **D** Representative IHC images of ki67 staining of tumor masses (left panels) and corresponding bar graph (right panel) of mice untreated (CTR) and treated with SIM 40 mg/kg, SIM 80 mg/kg and combination with scDb-hERG1-β1 16 mg/kg; 100 µm scale bar. The *p* values of Log Rank Test for Survival analysis was the following: CTR vs SIM 80: 0.7428; CTR vs SIM 40: 0.9271; CTR vs SIM 40 ± scDb-hERG1-β1: 0.1612. **P < 0.01 and ***P < 0.001. CTR control, SIM simvastatin, Hb_TOT_ total hemoglobin.

We then evaluated whether treatment with statins and their combination with the scDb-hERG1-β1 antibody would have an antineoplastic effect in vivo. To this purpose we generated an orthotopic PDAC mouse model obtained by ultrasound (US)-guided injection of PANC-1 cells into the pancreas [58]. Tumor growth was monitored by US and Photo Acoustic Imaging (PAI) before and after the treatments which started 22 days after cell inoculum when the volume of tumor masses reached the value of 9.2 ± 1.2 mm^3^. At the moment of mouse death tumor masses were collected and analyzed by IHC (see the treatments schedules in Fig. 8A, bottom panel and “Materials and methods”). We tested two different doses of SIM (40 and 80 mg/kg) and their combination with scDb-hERG1-β1 (16 mg/kg). In particular, at day 22 mice were randomized into five groups: (1) control (i.e., mice treated with the vehicle, administered daily by oral gavage), (2) SIM administered daily at 40 mg/kg by gavage, (3) SIM administered daily at 40 mg/kg by gavage + scDb-hERG1-β1 administered daily i.v. at 16 mg/kg, (4) SIM administered daily at 80 mg/kg by gavage and (5) SIM administered daily at 80 mg/kg by gavage + scDb-hERG1-β1 administered daily i.v. at 16 mg/kg. Mice were treated for further 21 days and the tumor growth was monitored weekly by US. SIM 40, alone or in combination with scDb-hERG1-β1 16 mg/kg only slightly reduced tumor volume, whereas SIM 80 mg/kg and SIM 80 mg/kg + scDb-hERG1-β1 16 mg/kg induced a significant reduction (*p*: 0.04 and *p*: 0.01, respectively) of volume compared to controls (Fig. 8A top panel and Fig. 8B). The treatment with SIM 80 and even more with SIM 80+scDb-hERG1-β1 produced a strong reduction of the total hemoglobin (Hb_TOT_) which mirrors the microvasculature within the tumor mass (Fig. 8C). Finally, SIM 80 mg/kg and even more its combination with scDb-hERG1-β1 significantly reduced the percentage of ki67 expressing cells (from 87% ± 1.55 s.e.m. in controls to 36% ± 1.87 s.e.m. in tumor masses from mice treated with SIM 80 (*p* = 0.002) and 5.4% ± 1.29 s.e.m. in in tumor masses from mice treated with SIM 80 +scDb-hERG1-β1 (*p* = 0.0006)) (Fig. 8D). Finally, the combined treatment with SIM 80+scDb-hERG1-β1 improved mice survival from 88.50 ± 3.2 s.e.m days to 107 ± 6.4 s.e.m. days (Log Rank Test *p* value 0.0676).

## Discussion

In the present study we show that the macromolecular complex that hERG1 channels form with β1 integrins in cancer cells [41]—the hERG1/β1 integrin complex—preferentially localizes in LRs in PDAC cells and primary sample. Such localization is relevant to drive a signal transduction pathway triggered by cell adhesion to the ECM and centered on activated β1 integrins and the complexed hERG1 channels, which involves PI3K and its downstream lipid mediators PIP2 and PIP3 and converge on the activation of Protein Kinase B/Akt. This pathway ultimately controls (i) Rac-1 activity and hence modifies the organization of f-actin to trigger cell motility and (ii) the expression of cyclins D1/E and p21 and hence controls cell cycle progression. Notably, the signaling pathway triggered by the hERG1/β1 integrin complex can be inhibited either disrupting LRs or inhibiting cholesterol synthesis by statins or specifically dissociating the hERG1/β1 integrin complex with a single chain bispecific antibody, scDb-hERG1-β1. The combined treatment with statins and the scDb-hERG1-β1 synergize to produce a strong inhibition of PDAC cell viability/proliferation and cell migration, both in vitro and in vivo. Such inhibitory effects depend on the presence of the hERG1/β1 integrin complex on the plasma membrane of PDAC cells. Particularly relevant from the therapeutic standpoint is the demonstration that statins and scDb-hERG1-β1 also sensitize PDAC cells to two chemotherapeutic drugs commonly used to treat PDAC patients: Oxaliplatin and Gemcitabine.

Lipid Rafts are “lipid-ordered platforms where proteins can segregate” [9] to trigger and regulate signaling pathways which are essential to cellular function in a spatially-controlled manner [11]. The spatial compartmentalization of signaling components generally defines the specificity and enhances the efficiency of signal transduction [21]. We show here that LRs control a signaling pathway which is triggered by integrin activation by the ECM and is centered on the recruitment of hERG1 potassium channels in cholesterol-rich membrane microdomains. Although we do not know the dynamics of hERG1 localization in LRs and whether this is only due to a driving effect of integrins, we confirm here that hERG1 localization in LRs strongly contributes to switching its function from an ion conducting channe [59] l to a signaling hub. Indeed, hERG1 in LRs recruits the p85 subunit of PI3K and specifically activates Akt and its downstream signaling. Interestingly, ERK signaling is not involved in this pathway, being ERK negatively regulated by caveolin-1 and its localization in rafts microdomains [60–63]. The localization in LRs was previously shown to affect the function of ion channels because (i) specific lipids can directly affect the functionality of e.g., voltage-dependent K^+^ channels (K_V_) including hERG1 [27, 64, 65] or (ii) LRs concentrate those protein kinases, e.g., Src kinase, that modulate K_V_ activity [64] through the involvement of caveolin-1. Although Src does not appear to be involved in the signaling pathway here presented [66], other signaling molecules could be concentrated in Lipid Rafts along with hERG1 and integrins, such as the non-receptor GEF Girdin and Gαi3 [41]. Despite speculations, data here provided clearly show that the hERG1/β1 integrin complex behave as a molecular device in cancer cells to regulate cell proliferation and migration Indeed, inhibiting the integrity and/or formation of lipid rafts, with either MβCD and combining these treatments with the bispecific antibody which harnesses and dissociates the hERG1/β1 integrin complex [41, 46] we obtained a strong reduction of both PDAC cell proliferation and migration. Interestingly, we obtained the same effects inhibiting 3-hydroxy-3-methylglutaryl-CoA reductase (HMGCR) through statins. The mechanism underlying these effects could be related to the effects of cholesterol on PIP which is known to stabilize the open state of the channel [67, 68] which in turn impairs the formation of the hERG1/β1 integrin complex [41]. Consistently, the combination of statins with the scDb-hERG1-β1 produces the strongest inhibitory effect on both cell vitality and migration of PDAC cells, while does not affect normal epithelial cells.

The pharmacological effect of statins which we show in the present paper merits great attention. Indeed, increasing evidence shows that statins can exert a relevant antineoplastic activity in various types of cancer, including PDAC, both in preclinical and clinical studies [69]. The anticancer activity of statins has been traced back to a series of cellular mechanisms, including the metabolic reprogramming [57, 70, 71] and their effects on Akt-centered signaling pathways [72, 73]. We here provide evidence that the latter effect can occur because statins alter the structure of LRs in cancer cells [56, 57] and in turn inhibit the signaling function of integrin-centered macromolecular complexes which involve the ion channel hERG1. Consistently, and particularly relevant from the therapeutic point of view, the combined treatment with statins and the bispecific antibody scDb-hERG1-β1- which harnesses the hERG1/β1 integrin complex has a strong antineoplastic effect in an orthotopic preclinical mouse model of PDAC, decreasing tumor volume and proliferative activity and strongly improving mice survival, compared to treatment involving chemotherapeutic drugs like Gemcitabine [47]. Notably, drug repurposing represents a novel frontier for antineoplastic treatment and hERG1 and its complexes may represent a novel target, as already demonstrated by the antineoplastic effects of the antibiotic Clarithromycin in colorectal cancer.

Data reported in the present paper indicates the repurposing of statins for antineoplastic therapy, in combination with a novel antibody scDb-hERG1-β1, for treating one of the most aggressive cancers, the Pancreatic Ductal Adenocarcinoma. Moreover, statins can be combined with chemotherapeutic agents, such as Gemcitabine or Oxaliplatin, allowing to decrease their doses and hence to reduce their toxicity. Overall, data shown in the present manuscript pave the way for a clinical protocol in which statins could be included in the treatment protocols for those PDAC patients whose cancer expresses the hERG1-β1 integrin complex.

## Materials and methods

### Antibodies and reagents

The following primary antibodies were used: anti-phospho AKT (Thr 308) (pAKT) r-pAb (Cell Signaling Technology, Massachusetts, Danvers, USA, cat. BK9275S) or anti-total-AKT (t-AKT) r-pAb (Cell Signaling Technology Massachusetts, Danvers, USA, cat. BK9272S) at final dilution 1:500 for WB; anti-phospho ERK1/2 (p-ERK1/2) (Thr202/tyr 204) r-mAb (Cell Signaling Technology Massachusetts, Danvers, USA, cat. 4370) at final dilution 1:1000 for WB; anti-total ERK1/2 (t-ERK1/2) pAb (Santa Cruz Biotechnology, Santa Cruz, CA, cat. (1-C16)sc93) at final dilution 1:200 for WB; r-pAb anti β1-integrin, RM-12 (Immunological Science, Rome, Italy) at final dilution 1:1000 for WB; r-pAb anti-hERG1, C54 (MCK Therapeutics Srl, Pistoia, Italy) at a final dilution 1:1000 for WB; m-mAb hERG1 (MCK Therapeutics Srl, Pistoia, Italy) 5 µg antibody/mg protein for Co-IP; the Alexa 488 conjugated mAb hERG1 was used at 1 μg/ml for FACS experiments (MCK Therapeutics Srl, Pistoia, Italy); m-mAb β1 TS2/16 (Ultra-LEAF™ Purified anti-human CD29 Antibody, Bio Legend, cat. 303035) 5 µg antibody/mg protein for Co-IP, 1:500 for IF; scDb-hERG1/β1 (Single chain diabody, MCK Therapeutics Srl, Pistoia, Italy), 20 μg/ml for cell treatment and for immunohistochemistry (IHC); scDb-hERG1/β1-alexa-488 20 μg/ml for IF; m-mAb Anti-α-Tubulin (Sigma-Aldrich cat. T9026) at 1:500 dilution for WB; anti-PIP2 (phosphatidylinositol 4,5-bisphophate) 1:200 for IF (MA3-500 Invitrogen, Waltham, MA, USA); anti-PIP3 (phosphatidylinositol 3,4,5-bisphophate) 1:100 for IF (A21328 Invitrogen, Waltham, MA, USA); anti-caveolin-1 pAb (Abcam, Cambridge, UK) at 1:1000 for WB. m-mAb Anti-caveolin-1 (610406, BD Biosciences, Franklin Lakes, NJ) at 1:1000 for WB and 1:50 for IF; m-mAb Anti-Flotillin-1 (sc-25506, Santa Cruz Biotechnology, Santa Cruz, CA) at 1:1000 for WB and 1:50 for IF, Anti-ARP2/3 complex (BS-12524R, Thermo Fisher Scientific, Waltham, MA) at 1:100 for IF. Anti-p21 (sc-397, Santa Cruz Biotechnology, Santa Cruz, CA) at 1:500 for WB. Anti-Cyclin E (sc-1987, Santa Cruz Biotechnology, Santa Cruz, CA) at 1:500 for WB. Anti-Cyclin D (2978, Cell signaling technology, Danvers, MA, USA) at 1:1000 for WB. Anti-Rat Ki-67 Antigen Clone MIB-5 (M7248, Dako, Glostrup, Denmark) at 1:50 for IHC. CellMask^TM^ Deep Red Actin staining reagent was (A57245, Invitrogen, Waltham, MA, USA) used according to manufacturer’s instructions. Secondary antibodies used for WB were: anti-rabbit immunoglobulin G (IgG) peroxidase antibody (1:10,000; whole molecule, A0545), anti-mouse IgG peroxidase antibody (1:5000; whole molecule, A4416) (Cell Signaling Technology, Danvers, MA, USA), IRDYe 800 CW anti-mouse (1:20,000, LI-COR Biosciences, Nebraska, USA); and IRDye 800CW anti-rabbit (1:20,000, LI-COR Biosciences, Nebraska, USA) and anti-goat (1:20,000, LI-COR Biosciences, Nebraska, USA) secondary antibodies. Alexa Fluor 546 goat anti-mouse, and CY2 goat anti-rabbit antibodies (Thermo Fisher Scientific, Waltham, MA) were used 1:500 and anti-6xHis antibody (Abcam, Cambridge, UK) was used 1:250 for IHC. Proteinase K (5 μg/ml final concentration, Sigma-Aldrich, Darmstadt, Germany) was used for antigen retrieval in IHC. A commercial kit was used for IHC: ImmPRESS^®^ Universal PLUS Polymer Kit (Vector Laboratories Inc., Newark, CA, USA). RAC 1 ACTIVATION KIT (17-283 Sigma Merck). Hoechst was used for staining nuclei in IF experiments (1:1000 in PBS, 45 min; Merck Sigma, Burlington, MA). Geneticin (0.8 mg/ml final concentration; G418, Thermo Fisher Scientific, Waltham, MA). Protein A/G Plus-Agarose for immunoprecipitation was purchased from Santa Cruz Biotechnology (sc-2003). Fibronectin (FN) was purchased from Sigma-Aldrich, Darmstadt, Germany, human plasma. Methylβ-cyclodextrin (MβCD) and Cholera Toxin subunit B (CTxB) were purchased from Sigma-Aldrich. Rhodamine-conjugated phalloidin was purchased from Invitrogen (Waltham, MA, USA) and used according to manufacturer’s instructions. SIM and FLUV were purchased from Merck Millipore. Both statins were dissolved in DMSO at 12 mM concentration (SIM) and 250 mM concentration (FLUV). Dulbecco’s Modified Eagle’s Medium High Glucose w/o Sodium Pyruvate w/o L-Glutamine (DMEM, Euroclone, Milan, Italy). L-glutamine (Euroclone, Milan, Italy). Fetal bovine serum (FBS, Fetal Bovine Serum EU Approved, Euroclone, Pero, Italy). RPMI (Euroclone, Milan, Italy). DMEM F-12 (Euroclone, Milan, Italy). RPMI 1640 (Life Technologies, Carlsbad, CA, USA), Keratinocyte medium—SFM (Life Technologies, Carlsbad, CA, USA). MEM Non-Essential Amino Acids 100X (Life Technologies, Carlsbad, CA, USA). Penicillin/Streptomicin 100X (Pen/Strep, Euroclone, Milan, Italy), Hepes (Life Technologies, Carlsbad, CA, USA), bovine pituitary extract (Life Technologies, Carlsbad, CA, USA), human recombinant EGF (Life Technologies, Carlsbad, CA, USA). Dulbecco’s Phosphate Buffer Saline w/o Ca w/o Mg (PBS, Euroclone, Milan, Italy). Trypsin-EDTA 1X in PBS w/o Ca w/o Mg w/o Ph Red (Euroclone, Milan, Italy). Trypan blue (Sigma, Darmstad, Germany). Bovine Serum Albumin (BSA, Sigma-Aldrich, Darmstadt, Germany). Hoechst (1:1000 final dilution; Merck Sigma, Burlington, MA). Prolong Diamond antifade mountant (Invitrogen, Waltham, Massachusetts, USA). Pierce 16% Formaldehyde Solution (w/v) Methanol-Free (PFA, 4% final concentration, Thermo scientific, Rockford, IL, USA). Triton X-100 (Sigma-Aldrich, Darmstadt, Germany). ECL Western detection system (Amersham, Buckinghamshire, UK). Protease inhibitors (Roche Complete Mini; Roche Diagnostics, Mannheim, Germany). Bradford protein assay (Bio-Rad, Hercules, CA). 10X Tris Buffered saline (TBS, Bio-Rad Laboratories S.r.l., Segrate, Milano, Italy). 10X Tween 20 (T, Bio-Rad Laboratories S.r.l., Segrate, Milano, Italy).

### Cells and culture

The human PANC-1, MiaPaCa2, BxPC3, HEK293 cell line were obtained from the American Type Culture Collection (ATCC). HEK293 cells expressing the hERG1 construct (HEK-hERG1) were prepared as previously described in [38] and maintained in complete culture medium supplemented with 0.8 mg/ml of Geneticin. HPDE were kindly gifted by Prof. I. Szabò (University of Padua, Italy); RLT-PSC cells were kindly gifted by Prof. F. Alves (UMG, Department of Hematology and Medical Oncology and the Institute for Diagnostic and Interventional Radiology, Goettingen, Germany). Cells were routinely cultured at 37 °C with 5% CO_2_ in a humified atmosphere. PANC-1, MiaPaCa2 and HEK293 were cultured in DMEM supplemented with 4 mM of L-glutamine and 10% FBS. BxPc-3 was cultured in RPMI supplemented with 2 mM of L-glutamine and 10% FBS. RLT-PSC were cultured in DMEM F-12 supplemented with 2 mM of L-glutamine and 10% FBS. HPDE were cultured in 50% RPMI 1640, 50% Keratinocyte medium—SFM supplemented with FBS 10% heat inactivated, MEM Non-Essential Amino Acids 1X, Pen/Strep 1X, Hepes 10 mM, bovine pituitary extract 0.025% and EGF human recombinant 2.5 μg/L. We certify that all the cell lines used in the present study were routinely screened for Mycoplasma contamination, and only Mycoplasma negative cells were used. Silencing of *hERG1* in PANC-1 and MiaPaCa2 cells, was carried out with siRNAs as previously described in [73].

### Cell treatments

Vitality and cell cycle FACS analysis were performed on cells after 24 h of treatments in culture medium with 10% of FBS. All other experiments were performed on cells seeded on Fibronectin (FN). The coating with FN was performed following the standard protocol provided with the product. FN was diluted in sterile PBS at 5 µg/cm^2^ concentration. The culture surface was coated with a minimal volume. The dishes were left air-drying for 1 h at room temperature before introducing cells and medium. Cells were starved overnight prior to seeding onto fibronectin coatings and the kept in serum-free medium for the entire duration of the experiments. *Treatment with methyl β-cyclodextrin*: The treatment with methylβ-cyclodextrin (MβCD) was performed for FACS, IF, co-immunoprecipitation, western blot, and motility experiments at 5 mM for 20 min at 37 °C [48]; this treatment is in accord with [70], in fact in this paper they used MβCD at 0.67 mM for 240 min. After treatment, cells were collected and prepared for experimental procedures. *Treatment with scDb-hERG1-β1 antibody:* scDb-hERG1-β1 was added in the serum-free-BSA medium at the final concentration of 20 μg/ml for 90 min for IF and motility experiments. For viability experiments was add at IC50: HEK293: 200 µM; MiaPaCa2: 20.40 µM; PANC-1: 18.13 µM for 24 h. *Treatment with statins*: Following the procedure in [56], cells were pretreated overnight with statins (20 μM) the day before the experiments. The treatment with SIM was performed at IC50 concentration for the different cell lines for viability experiments for 24 h. The treatment with SIM was performed at IC50 concentration for the different cell lines for IF and motility experiments for 90 min in the serum-free-BSA medium. HEK293: 7.57 µM; MiaPaCa2: 4.16 µM; PANC-1: 4.47 µM. The treatment with SIM was performed at 20 µM for cholesterol levels for 90 min in the serum-free-BSA medium. The treatment with FLUV was performed at IC50 concentration for the different cell lines for viability experiments for 24 h. The treatment with FLUV was performed at IC50 concentration for motility experiments for 90 min in the serum-free-BSA medium HEK293: 3.45 µM; MiaPaCa2: 3.45 µM; PANC-1: 2.47 µM. The treatment with LOVA was performed at IC50 concentration for the different cell lines for viability experiments for 24 h. The treatment with LOVA was performed at IC50 concentration for motility experiments for 90 min in the serum-free-BSA medium HEK293: 3.26 µM; MiaPaCa2: 2.07 µM; PANC-1: 2.78 µM. The treatment with ATOR was performed at IC50 concentration for the different cell lines for viability experiments for 24 h. The treatment with ATOR was performed at IC50 concentration for motility experiments for 90 min in the serum-free-BSA medium HEK293: 2.09 µM; MiaPaCa2: 3.13 µM; PANC-1: 1.80 µM.

### Cell viability assay

Cell viability was measured by the trypan blue exclusion test as in [49]. After incubation with the drug and the scDb-hERG1-β1 antibody, the trypan blue dye was added to the cells and live cells were counted using LUNA-II™ Automated Cell Counter (Logos Biosystems, Villeneuve d’Ascq, France). The 50% inhibitory concentration (IC_50_) was calculated using the equation Y = Min + Max − min1 − (XIC50) Hill coefficient, as in [49]. Combination Index (CI) calculation was performed as previously described in [74].

### Rac1 activation assay

Rac Activation was measured by Rac1 activation kit. After treatment 1X Assay/Lysis Buffer was added to the cells (0.5–1 mL per 100 mm tissue culture plate). The culture plates were put on ice for 10–20 min and the cells were detached from the plates by scraping with a cell scraper. The lysates were cleared by centrifugation for 10 min (14,000 × *g* at 4 °C). The supernatant was collected. Positive and negative controls were produced: 10 µL of 100X GTPγS was added to one tube of lysate to generate positive control and 10 µL of 100X GDP to the other tube was added to produce negative control; the tubes were incubated for 30 min at 30 °C with agitation and the reaction was stopped by adding 65 µL of 1 M MgCl_2_ to each tube. For samples: the volume was adjusted to 1 mL with 1X Assay Lysis Buffer and resuspend the PAK PBD Agarose bead slurry by vortexing. Quickly add 40 µL of resuspended bead slurry to each tube (including GTPγS/GDP controls), the tubes were incubated at 4 °C for 1 h with gentle agitation. The beads were pelleted by centrifugation for 10 s at 14,000 × *g*. The supernatant was discarded, making sure not to disturb/remove the bead pellet. The beads were washed 3 times with 0.5 mL of 1X Assay Buffer, centrifuging and aspirating each time. After the last wash, the beads were pelleted and carefully all the supernatant was discarded. The bead pellet was resuspended in 40 µL of 2X reducing SDS-PAGE sample buffer. Each sample was boiled for 5 min and then centrifuged for 10 s at 14,000 × *g*. 20 µL/well of pull-down supernatant was loaded to a polyacrylamide gel. Western Blotting was performed as described below.

### Lateral motility assay

Lateral motility was determined using 35 mm dishes and drawing 15 horizontal lines and 3 perpendicular lines on the dish bottom to generate a grid system. Plates were coated with FN and 5 × 10^5^ cells were seeded and allowed to attach for 5–10 min. Then, a manual scratch was carried out and the width of the wound was determined (W0). At this time, different treatments were added. Then, dishes were incubated for further 90 min except for MβCD which was incubated in the last 20 min of seeding. At the end of incubation, the width of the wounds (Wt) was determined on unfixed cells. We took care to do these measurements within not more than 30 min overall. Three wounds were drawn following the 3 horizontal lines. Then, the distances between cells were measured at each mark point (where the 3 horizontal lines crossed the 15 vertical lines) using a light microscope. The widths measured at time 0 correspond to the W0 parameter. These different 45 points were measured again after 90′. Motility Index (MI) was assessed using the following formula: MI = 1 – Wt/W0, where Wt is the width of the wounds after 90 min and then converted in percentage.

### Sucrose-gradient fractionation and immunoblotting analysis

Lipid raft fractions were isolated as previously described in [48]. Briefly, 7 × 10^6^ PANC-1 or HEK-hERG1 cells were suspended in 1 mL of lysis buffer, containing 1% Triton X-100 10 mM Tris-HCl (pH 7.5), 150 mM NaCl, 5 mM EDTA, 1 mM Na_3_VO_4_ and 75 U of aprotinin and allowed to stand for 20 min. The lysate was first mechanically homogenized (10 strokes) and then centrifuged for 5 min at 1300 × *g* to remove nuclei. The supernatant fraction was first mixed with an equal volume of 85% (w/v) sucrose-containing lysis buffer (10 mM Tris-HCl, pH 7.5, 150 mM NaCl, 5 mM EDTA) and then placed at the bottom of a linear sucrose gradient (5–30%). The sucrose gradient was centrifugated at 200,000 × *g* for 18 h at 4 °C using a SW41 rotor (Beckman Institute, Palo Alto, CA, USA) and eleven fractions (1-ml each) were collected from the top to the bottom of the gradient tube. The fractions 4–6 (Triton X-100-insoluble fractions) and the fractions 9–11 (Triton X-100-soluble fractions) of the sucrose gradient were considered as raft fractions and non-raft fractions, respectively, as determined by Western blotting using anti-caveolin-1 pAb. The fraction samples were loaded by volume and subjected to sodium dodecyl sulfate-polyacrylamide gel electrophoresis (SDS-PAGE) (7.5%). The proteins were electrophoretically transferred onto polyvinylidene difluoride (PVDF) membranes (Bio-Rad) blocked with 5% bovine serum albumin (BSA) in TBS containing 0.05% Tween 20 (TBS/T) and washed with TBS/T. PVDF were probed with anti-hERG1 (C54) pAb or anti-β1 integrin (RM12) pAb or anti-caveolin-1 pAb. After washing with TBS/T, bound antibodies were visualized with HRP-conjugated anti-rabbit IgG and immunoreactivity was assessed by chemiluminescence reaction, using the ECL. Densitometric scanning analysis was performed by Mac OS X (Apple Computer International), using NIH Image 1.62 software. The density of each band in the same gel was analyzed, values were totaled, and then the percentage distribution across the gel was detected.

### Protein extraction, co-immunoprecipitation, dot-blot and western blotting

*Protein extraction*: adherent cells were first washed with ice-cold PBS and then collected by scraping. Pellets were obtained by centrifugation at 1200 rpm, washed twice in PBS and then immediately incubated for 20 min in 1% NP-40 lysis buffer (1% NP-40, 150 mM NaCl, 50 mM Tris-HCl, pH 8, 5 mM EDTA, 10 mM Na_4_P_2_O_7_) supplemented with a tablet of a complete mix of protease inhibitors. All the procedures were performed maintaining samples on ice. Lysates were centrifuged at 13,000 × *g* for 10 min at 4 °C. Supernatants were then collected and assayed for protein concentration using Bradford protein assay following manufacturer instructions. *Co-immunoprecipitation*: pooled Triton X-100-insoluble fractions (4–5–6) or pooled Triton X-100-soluble fractions (9–10–11) or samples (1.5 mg of protein) were subjected to a pre-clearing step, consisting of a 2 h incubation at 4 °C under rotation with Protein A/G Plus-Agarose beads following manufacturer’s instructions. Thereafter, cell lysates were immunoprecipitated with anti-hERG1 mAb or TS2/16. Immunoprecipitation was also performed with an irrelevant polyclonal IgG, as a negative control. The immunoprecipitates (IPs) were split into two aliquots. The first one was subjected to Western blot analysis for hERG1 or integrin β1 detection; the second one was checked by dot blot for GM1 detection.

#### Dot-blot analysis of immunoprecipitation

Briefly, aliquots of hERG1 IPs, prepared as described above, were spotted onto nitrocellulose strips. The strips were blocked for 1 h with 5% BSA in TBS/T (Bio-Rad) to block the residual binding sites on the paper. The strips were rinsed for 10 min in TBS/T and then incubated with Cholera Toxin B Subunit-Peroxidase from Vibrio Cholerae for 1 h at 25 °C, or with anti-hERG1 pAb or with anti-integrin β1 pAb and further incubated for 1 h at 37 °C with HRP-conjugated anti-rabbit IgG. Immunoreactivity was assessed by chemiluminescence reaction, using the ECL Western detection system.

Samples were denatured in 4X Laemmli buffer at 95 °C for 5 min and then run by Sodium dodecyl Sulfate-Poly-Acrylamide Gel Electrophoresis (SDS-PAGE). 7.5% polyacrylamide gels were used for hERG1, β1-Integrin, p85, pAKT, AKT, pERK, ERK, while 10% gels were used for pAkt/Akt and pERK/ERK analysis. 4–20% gradient polyacrylamide gels were used for Cyclin D, Cyclin E, p21. Blotting was performed using the TurboBlotTM (Bio-Rad, Hercules, CA) “MIXED MW” program (1.3 A, 25 V for 7 min) for pAkt/Akt, while the “HIGH MW” program was selected for hERG1 and β1 integrin. WB was performed using primary and secondary antibodies diluted in T-phosphate-BSA at the concentrations indicated in “Chemicals and antibodies”. Membranes blocking and washing was performed as described for co-immunoprecipitation experiments. Immunoreactivity was revealed by using IRDYe 800 CW anti-mouse and anti-rabbit secondary antibody (Concentrations reported in “Chemicals and antibodies”) and the LI-COR Odyssey Scanner apparatus (LI-COR Biosciences, Lincoln, NE).

Original membranes are reported in the Supplemental Material File.

### Densitometric analysis

Densitometric analysis was performed, as reported in [38], using ImageJ software (ImageJ 1.38, U.S. National Institutes of Health) after background subtraction. When quantifying protein expressions in insoluble vs soluble membrane fractions, the expression of the co-immunoprecipitated protein (either GM1 or the β1 integrin) was first divided by the signal of the protein used to immunoprecipitate and then normalized over total hERG1 present in the total lysate. When quantifying hERG1/β1 integrin complex, the signal for the co-immunoprecipitated protein hERG1 was first divided by the signal of the protein used for immunoprecipitation (β1 integrin) and then normalized to the signal of the corresponding protein in the total lysate (input hERG1). To evaluate the presence of a third protein (i. e. caveolin-1 or flotillin) associated with the hERG1/β1 integrin complex the specific protein was immunoprecipitated using the same anti-integrin β1 mAb. When quantifying hERG1/β1 integrin/caveolin-1 or flotillin complex, the signal of caveolin-1 or flotillin-1 was first divided by the signal of the β1 integrin protein and then normalized to the signal of the corresponding protein in the total lysate (input caveolin-1 or flotillin-1).

The hERG1 protein appears in WB membranes as a 135 kDa band (the core glycosylated protein, present in the ER) and two 150–155 kDa bands. These correspond to the fully glycosylated hERG1 protein, which is found in both the plasma membrane and intracellular compartments (i.e., the Golgi apparatus, during hERG1 trafficking towards the plasma membrane, and endosomes, during channel degradation). This has been previously described in [41, 75, 76].

### Immunohistochemistry

172 formalin-fixed, paraffin-embedded PDAC samples were analyzed for the expression of the hERG1/β1 Integrin complex (commercial tissue microarray number: PA2082a, BioMax). After dewaxing and rehydrating the sections, endogenous peroxidases were blocked with a 1% H_2_O_2_ solution in PBS. Subsequently, antigen retrieval was performed by treatment with proteinase K (5 μg/ml) in PBS at 37 °C for 5 min for scDb-hERG1/β1 (20 μg/ml) and anti-caveolin (1:50, see “Reagents and antibodies” section). Incubation with the primary antibodies was carried out overnight at 4 °C. For scDb-hERG1-β1, sections were incubated with anti-6xHis antibody for 90 min at RT (1:250 final dilution). Immunostaining was performed with a commercially available kit, according to the manufacturer’s instructions. Tumor masses of mice were analyzed for ki67 expression. After dewaxing and rehydrating the sections, endogenous peroxidases were blocked with a 1% H_2_O_2_ solution in PBS. Subsequently, antigen retrieval was performed by treatment with Citrate buffer pH 6 at 700 for 12 min. The sections were stained with anti-ki67 antibody (1:50, see “Reagents and antibodies” section). Incubation with the primary antibody was carried out overnight at 4 °C. Immunostaining was performed with a commercially available kit, according to the manufacturer’s instructions.

### Flow cytometry

Flow cytometry was performed to assess hERG1 and β1 expression pretreating cells 20 min with FN or TS2/16 in order to stimulate hERG1/β1 complex formation. Cells were then revealed using TS2/16 and mAb hERG1-Alexa 488 (see “Reagents and antibodies” section) using BD FACSCanto Flow Cytometer as in [41]. Cyclodextrin treatment was performed the day before the experiments. Acquisition and analysis were performed using FACS Diva software (BD Biosciences). Values are expressed as mean fluorescence intensity of the area under the curve, indicated as MFI. Cell cycle distribution was assessed by flow cytometry after staining the cells with propidium iodide (PI).

### Immunofluorescence (IF)

*Staining for hERG1/β1 integrin complex* on cells was performed following the protocol previously described in [41, 46]. After 2 h of blocking in PBS with 10% BSA, slides were incubated for further 2 h with scDb-hERG1/β1-alexa 488 (20 µg/ml final concentrations). Nuclei were stained with Hoechst and slides were mounted using Prolong Diamond antifade mountant (see “Reagents and antibodies” section). *Staining with anti-caveolin monoclonal antibody* (1:50) was followed by secondary Alexa-546-anti-mouse antibody (1:500) (see “Reagents and antibodies” section). *Staining with anti-PIP2 (1:200) and PIP3 (1:100) monoclonal antibodies* was followed by secondary Alexa-647-anti-IgG mouse antibody (1:500) (see “Reagents and antibodies” section). *Staining with anti-Arp2/3 complex antibody*. Cells were fixed using 4% PFA, followed by permeabilization with 0.1% Triton-X (see “Reagents and antibodies” section) and blocking with 10% bovine serum albumin (BSA) (see “Reagents and antibodies” section) in PBS. Staining with anti-Arp2/3 complex (1;100 in 5% BSA) (see “Reagents and antibodies” section) was performed O/N at 4 °C and followed by incubation with secondary goat anti-rabbit CY2 antibody (1:500 in PBS) for 60 min in the dark at room temperature. CellMask^TM^ Deep Red Actin (1:1) staining was then performed for 15 min in the dark at room temperature. For all IF experiments, nuclei were stained with Hoechst (1:1000 in PBS, 5 min) and slides were mounted using Prolong Diamond antifade mountant. IF on primary samples. IF on a small PDAC subset, examined from the residual samples of [47], was performed using scDb-hERG1/β1-alexa 488 and Caveolin-1. Paraffin embedded tissues have been processed. After dewaxing (three passages of 20 min each in xylene) and rehydrating the sections, antigen retrieval was performed by treatment with proteinase K (5 μg/ml) in PBS at 37 °C for 5 min. Blocking was performed with BSA 1% for 30 min. Firstly, we have incubated with Caveolin-1 (1:50) O/N at 4 °C. The following day samples were incubated for 1 h with secondary Alexa-546-anti-mouse antibody (1:500) (see “Reagents and antibodies” section). Afterwards, scDb-hERG1/β1-alexa 488 (20 μg/ml final concentration) was incubated for 2 h. Nuclei were stained with Hoechst (1:1000 in PBS, 30 min).

All images were captured using confocal microscope, Nikon Eclipse TE2000-U (Nikon, Tokyo, Japan) and analyzed using ImageJ software. Cortical actin density was quantified using the dedicated PlasMACC Fiji Plugin [77], while actin stress fibers length was quantified following the method described in [41]. The fluorescence relative to at least 20 cells taken from 3 different microscopic fields was determined using ImageJ Software. The same procedure was repeated in 3 separate experiments, per each experimental condition. The fluorescence intensity was measured for each cell and divided by the cell area, to obtain a final “normalized fluorescence”. The means of the ”normalized fluorescence” values obtained in each replicate was then used to calculate the “mean fluorescence intensity” values ± standard error means in the different experiments. Across different experiments, images were acquired during the same confocal microscopy session. Moreover, for each acquisition the same script, regarding microscopy settings, was maintained.

### High-performance thin layer chromatography (HPTLC) analysis of cholesterol

Cells in the presence or in the absence of MβCD or SIM were lysed in lysis buffer containing 1% Triton X-100 for 20 min at 4 °C. After evaluation of the protein concentration the lysate was subjected to cholesterol analysis. Neutral lipid extracts were separated by HPTLC, using a solvent system of hexane/diethyl ether/acetic acid (70:30:1, v/v/v) and were detected by staining with 2% copper acetate solution in 8%phosphoric acid and subsequent heating at 120 °C for 15 min. Quantitative analysis was performed using NIH Image1.62 (Mac OS X, Apple Computer International).

### In vivo experiments

*PDAC Xenograft.* Panc-1 cells were resuspended in PBS and injected subcutaneously (1 × 10^6^ cells/injection) into both lateral flanks of Athymic Nude-Foxn1^nu/nu^ (Envigo, Indianapolis, ID, USA). Once tumors reached an average size of mm^3^, mice were randomly assigned to four groups of treatments and administered by oral gavage with SIM (80 mg/kg daily and 40 mg/kg daily), intravenously (iv) with scDb (16 mg/kg daily), combination of SIM and scDb, or vehicle for 30 days. SIM was dissolved in 0.5% methyl cellulose (PRODOTTI GIANNI Srl) freshly prepared every week. Mice belonging to the vehicle group were treated with methyl cellulose by gavage daily. Mice were monitored until spontaneous death, and the tumors were harvested for immunohistochemical analysis. The investigators were blinded throughout the study. *Ultrasound Imaging.* After anesthesia with isoflurane (induction dosage of 4% and a maintenance dose of 2%), mice were placed in a prone position on a heated pad at 37 °C, and body temperature, respiration rate, and ECG were continuously monityored. Ultrasonic transmission gel was used on the mice skin. The VevoLAZR-X imaging platform (Fujifilm VisualSonics) was used for ultrasonic imaging on B-mode modality and axial 3D acquisition of the tumor masses were carried out with a 55-MHz transducer. The Vevo LAB software was then used to define the regions of interest (ROI) for each frame, allowing to rendering 3D the tumors and measuring the volumes. 3D PA imaging with OxyHemo-Mode was used to determine oxygen saturation (sO_2%_) and blood hemoglobin (Hb) comparing the PA signal at 750 and 850 nm. Data obtained during the imaging were post-processed using Vevo LAB software (FUJIFILM VisualSonics). For studying tumor oxygenation sO_2_ (%) was measured in all the tumors. Moreover, to determine the presence of blood inside the tumors, total blood hemoglobin (Hb_TOT_), that represents the amount of hemoglobin in the mass, was measured with PA. *Survival analysis.* We performed Log Rank test to show the fraction of mice living for a certain amount of time after treatment*.*

### LDH assay

Samples of the culture medium were collected at the desired experimental time points by removing 2–5 μl of medium and diluting into 48–95 μl LDH Storage Buffer (Promega).

Optional: If a Maximum LDH Release Control is required, it’s possible to add 2 μl of 10% Triton X-100 (per 100 μl original volume) to the vehicle-only wells, mix and incubate for at least 10–15 min before sample removal. After collecting and diluting all samples 50 μl of diluted sample were transferred into a 96-well opaque-walled, non-transparent assay plate (with clear or opaque bottom). 50 μl of LDH Detection Reagent (Promega) were added to each well. Plates were then incubated for 60 min at room temperature. Luminescence was recorded after 30–60 min after adding LDH Detection Reagent. ${\rm{Cytotoxicity}}( \% )=100x\frac{({\rm{Experimental\; LDH\; Release}}-{\rm{Medium\; Background}})}{({\rm{Maximum\; LDH\; Release\; Control}}-{\rm{Medium\; Background}})}$

### Statistical analysis

For western blot analysis the statistical procedures were performed by GraphPad Prism software Inc. (San Diego, CA, USA). D’Agostino-Pearson omnibus normality test was used to assess the normal distribution of the data. Normally distributed variables were summarized using the mean ± standard deviation (SD). Differences between numerical variables were tested using Paired t-test. The log-rank test was performed to assess significance between mean survival in Kaplan–Meier curves, computed by GraphPad Prism software Inc. (San Diego, CA, USA). *p ≤ 0.05, **p ≤ 0.005, ***p ≤ 0.001, ****p ≤ 0.0001.

## Supplementary information

Supplementary figures and legends

Supplemental material file with original uncropped membranes

Supplementary Table S1
