# Supplementary material for: Targeting the hERG1/β1 integrin complex in lipid rafts potentiates statins anti-cancer activity in pancreatic cancer
Source: Cell Death Discov. 2025 Feb 3;11:39. doi: 10.1038/s41420-025-02321-2 (PMC11790905; doi:10.1038/s41420-025-02321-2)
Supplement: Supplementary file 1 — Supplementary figures and legends [file 41420_2025_2321_MOESM1_ESM.docx]

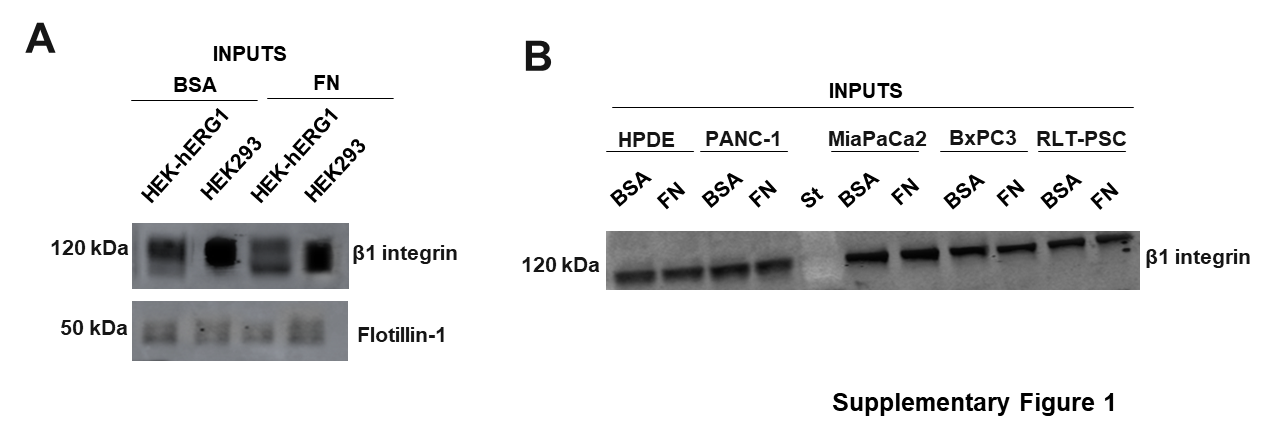


**Supplementary Figure 1. (A)** Representative membrane of inputs of β1 integrin and flotillin-1 in HEK-hERG1 cells after cell seeding on FN for 90 min. HEK-293 were used as control. **(B)** Representative membrane of inputs of β1 integrin in normal and PDAC cells, following 90 min adhesion onto BSA or FN. BSA: bovine serum albumin. FN: fibronectin. ST: standard.


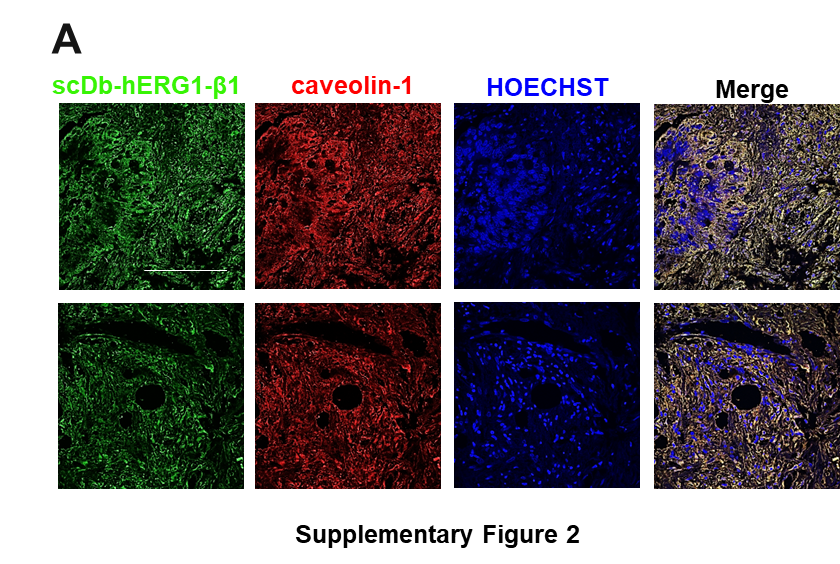


**Supplementary Figure 2.** **(A)** Representative IF images showing co-localization of hERG1-β1 integrin complex and caveolin-1 in a PDAC tissue sample. Sample was stained with scDb-hERG1-β1 alexa-488 conjugated and anti-caveolin-1 antibody revealed with Alexa-546-anti-mouse secondary antibody. From left to right, scDb-hERG1-β1-alexa488, caveolin-1, Hoechst staining and Merge images are reported (scale bar: 100 µm).


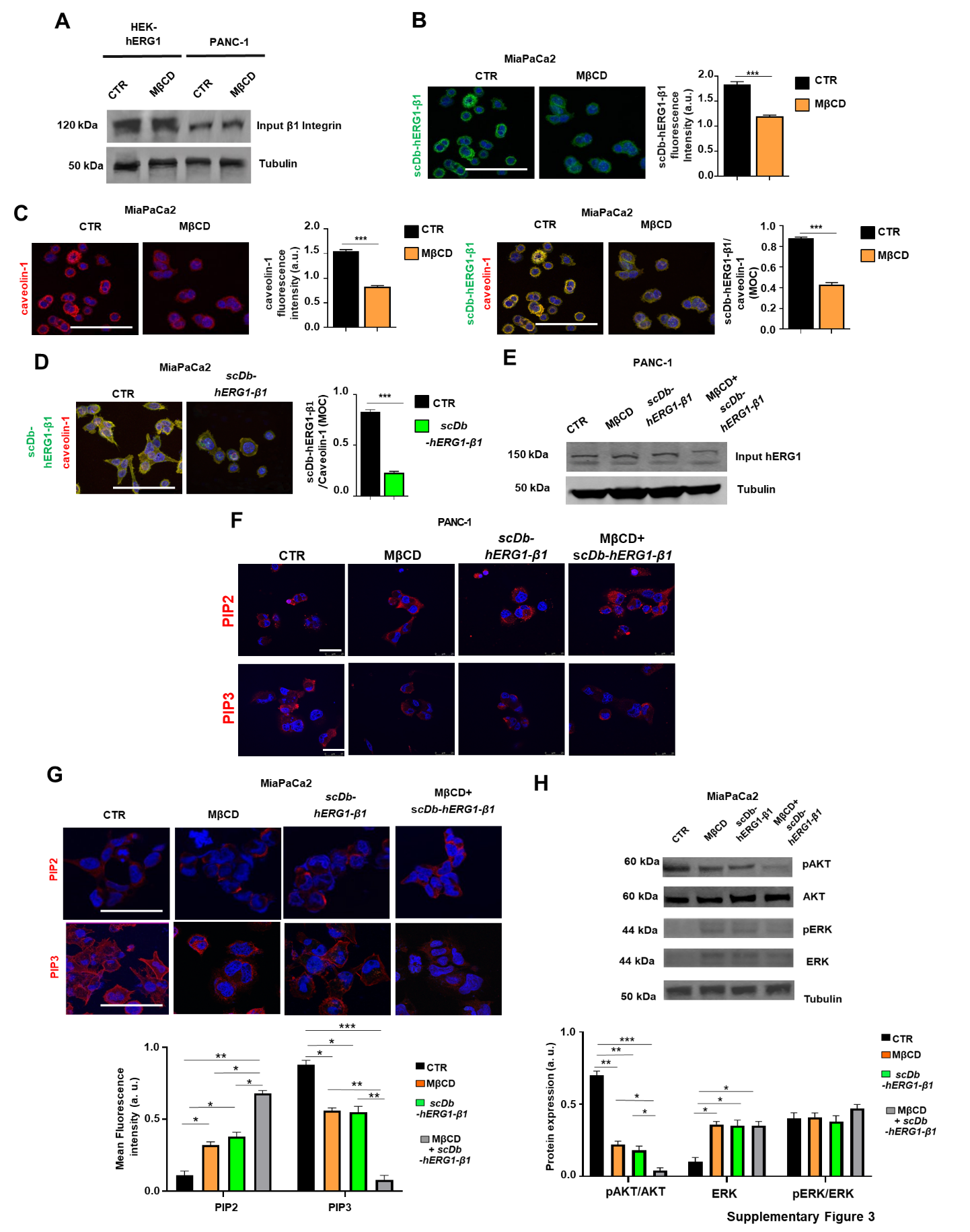


**Supplementary Figure 3. (A)** Representative membrane of inputs of β1 integrin and Tubulin in HEK-hERG1 and PANC-1 cells (CTR) and treated with MβCD (5mM), seeded on FN for 90 min. (**B)** IF performed on MiaPaCa2 cells following 90 min adhesion onto FN with or without treatment with 5 mM MβCD. Representative images (scale bar: 100 μm) of scDb-hERG1-β1 staining staining is on the right. a.u.= arbitrary units. At least a total of 20 cells per condition from three independent experiments (*n*=3) were analyzed. **(C)** IF performed on MiaPaCa2 cells following 90 min adhesion onto FN with or without treatment with 5 mM MβCD. Representative images (scale bar: 100 μm) of caveolin-1 staining and colocalization between scDb-hERG1-β1 and caveolin-1 are on the left, while quantitative analyses (fluorescent intensity and Mander’s Overlapping Coefficient, MOC) are reported in the graphs on the right. a.u.= arbitrary units. At least 20 cells (in 3 different fields) per condition from three independent experiments (*n*=3) were analyzed. All data are presented as mean values ± s.e.m. **(D)** IF performed on MiaPaCa2 cells following 90 min adhesion onto FN with or without treatment with scDb-hERG1-β1 (20μg/ml). Representative images of colocalization between scDb-hERG1-β1 and caveolin-1 (scale bar: 100 μm) are on the left, while quantitative analyses (MOC) are reported in the graph on the right. a.u.= arbitrary units. At least 20 cells (in 3 different fields) per condition from three independent experiments (*n*=3) were analyzed. All data are presented as mean values ± s.e.m. **(E)** Representative membrane of inputs of hERG1 and Tubulin in PANC-1 cells (CTR) and treated with MβCD (5mM), scDb-hERG1-β1 (20μg/ml) and their combination, seeded on FN for 90 min. **(F)** Lower magnification images of PIP2 (top panels) and PIP3 (bottom panels) of IF (reported in figure 3G) performed on PANC-1 cells untreated (CTR) or treated with MβCD (5mM), scDb-hERG1-β1 (20ug/ml), and their combination, seeded on FN for 90 min. (scale bar: 25 μm). At least 20 cells (in 3 different fields) per condition from three independent experiments (*n*=3) were analyzed. All data are presented as mean values ± s.e.m. **(G)** IF performed on MiaPaCa2 cells untreated (CTR) or treated with MβCD (5mM), scDb-hERG1-β1 (20ug/ml) and their combination, seeded on FN for 90 min. Representative images of PIP2 (top panels) and PIP3 (bottom panels) (scale bar: 100 μm) are on the top, while quantitative analyses (Mean fluorescence intensity) are reported in the graph on the bottom. At least 20 cells (in 3 different fields) per condition from three independent experiments (*n*=3) were analyzed. All data are presented as mean values ± s.e.m. **(H)** Representative blot (top) and densitometric analysis (bottom) of phospho-Akt, ERK and phospho-ERK levels in MiaPaCa2 cells untreated (CTR) or treated with MβCD (5mM), scDb-hERG1-β1 (20µg/ml) and their combination, seeded on FN for 90 min. Data are presented as mean values ± s.e.m. (*n*=3). a.u. = arbitrary units. Membranes were probed with anti-pAkt Thr308, anti-Akt Thr308, ERK1/2 (pERK1/2) (Thr202/tyr204) and anti-total ERK1/2 antibodies. *P < 0.05; **P < 0.01, and ***P < 0.001 (One-Way ANOVA). CTR: control; MβCD: Methyl-β-cyclodextrin; MOC: Mander’s Overlapping Coefficient.


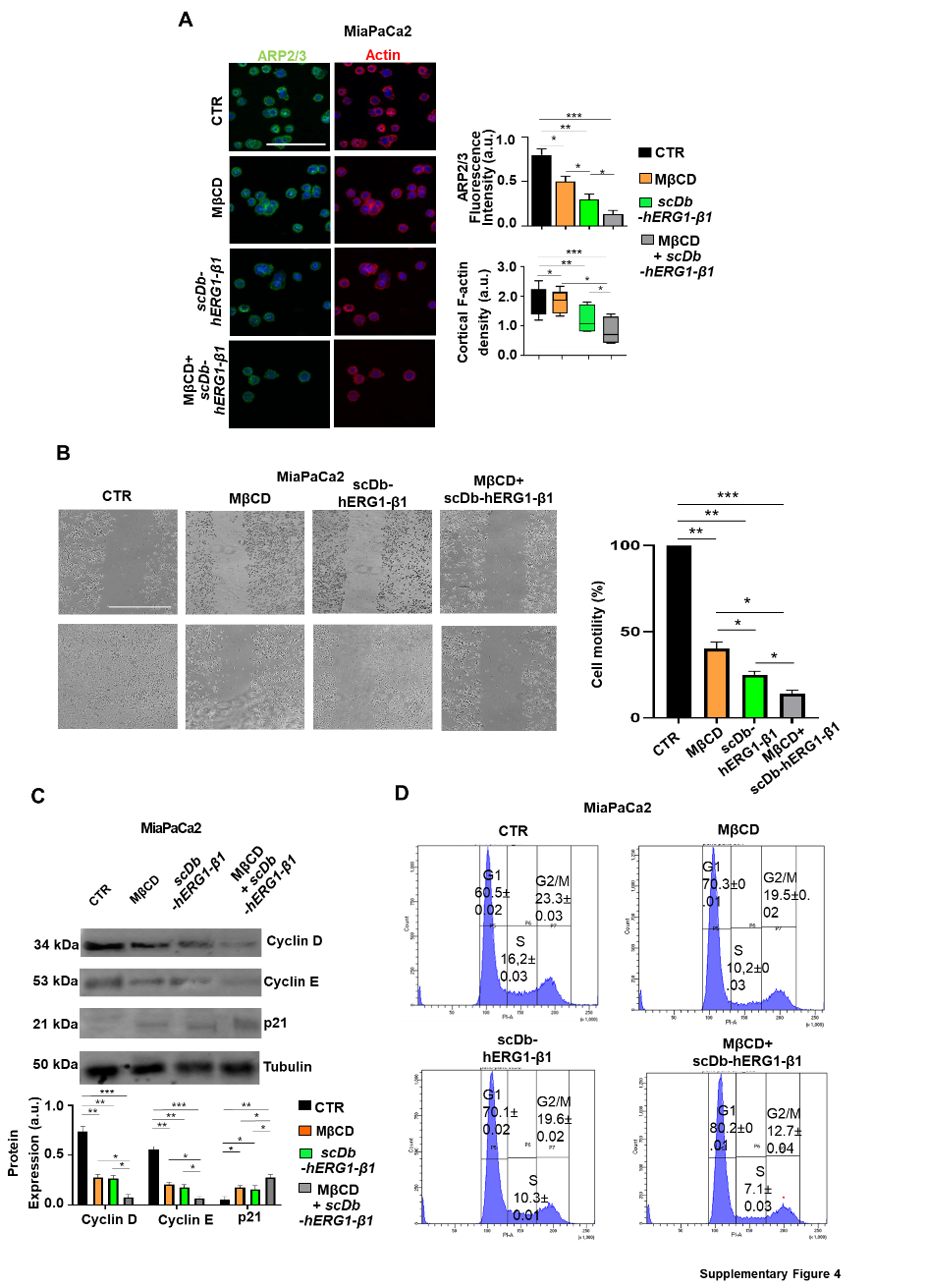


**Supplementary Figure 4. (A)** IF images of MiaPaCa2 cells untreated (CTR) or treated with MβCD (5mM), scDb-hERG1-β1 (20µg/ml) and their combination, seeded on FN for 90 min, stained with anti-ARP2/3 antibody and cortical F-actin (left panels). Scale bar: 100 µm. At least 20 cells (in 3 different fields) per condition from three independent experiments (*n*=3) were analyzed. Quantification graphs of ARP2/3 fluorescent intensity and cortical F-actin density were reported in the right panels. Data are presented as mean values ± s.e.m. **(B)** Lateral motility experiments onto FN were performed on MiaPaCa2 cells treated with MβCD (5mM), scDb-hERG1-β1 (20μg/ml) and their combination onto FN for 90 mins. Representative images are reported in the left panel. Scale bar: 100 µm. The motility is reported as graph of percentage of cell motility in the right panel. Data are presented as mean values ± s.e.m. (*n*=3). **(C)** Representative blot (top) and densitometric analysis (bottom) of Cyclin D, Cyclin E and p21 in MiaPaCa2 cells untreated (CTR) or treated with MβCD (5mM), scDb-hERG1-β1 (20μg/ml) and their combination, seeded on FN for 90 min. Data are presented as mean values ± s.e.m. (*n*=3). a.u. = arbitrary units. Membranes were probed with anti-Cyclin D, anti-Cyclin E and anti-p21 antibodies. **(D)** Flow cytometry (FC) plots of cell cycle of MiaPaCa2 cells treated with MβCD (5mM), scDb-hERG1-β1 (20μg/ml) and their combination for 24 hours. Data are presented as mean values ± s.e.m. (*n*=3). MβCD vs CRT: p=0.007 (G1); p=0.049 (S); p=0.048 (G2/M). scDb-hERG1-β1 vs CRT: p=0.008 (G1); p=0.042 (S); p=0.038 (G2/M). MβCD+scDb-hERG1-β1 vs CTR: p=0.0005 (G1); p=0.004 (S); p=0.0004 (G2/M). Data are presented as mean values ± s.e.m. (*n*=3). P < 0.05; **P < 0.01, and ***P < 0.001 (One-Way ANOVA). CTR: control; MβCD: Methyl-β-cyclodextrin. MOC: Mander’s Overlapping Coefficient.


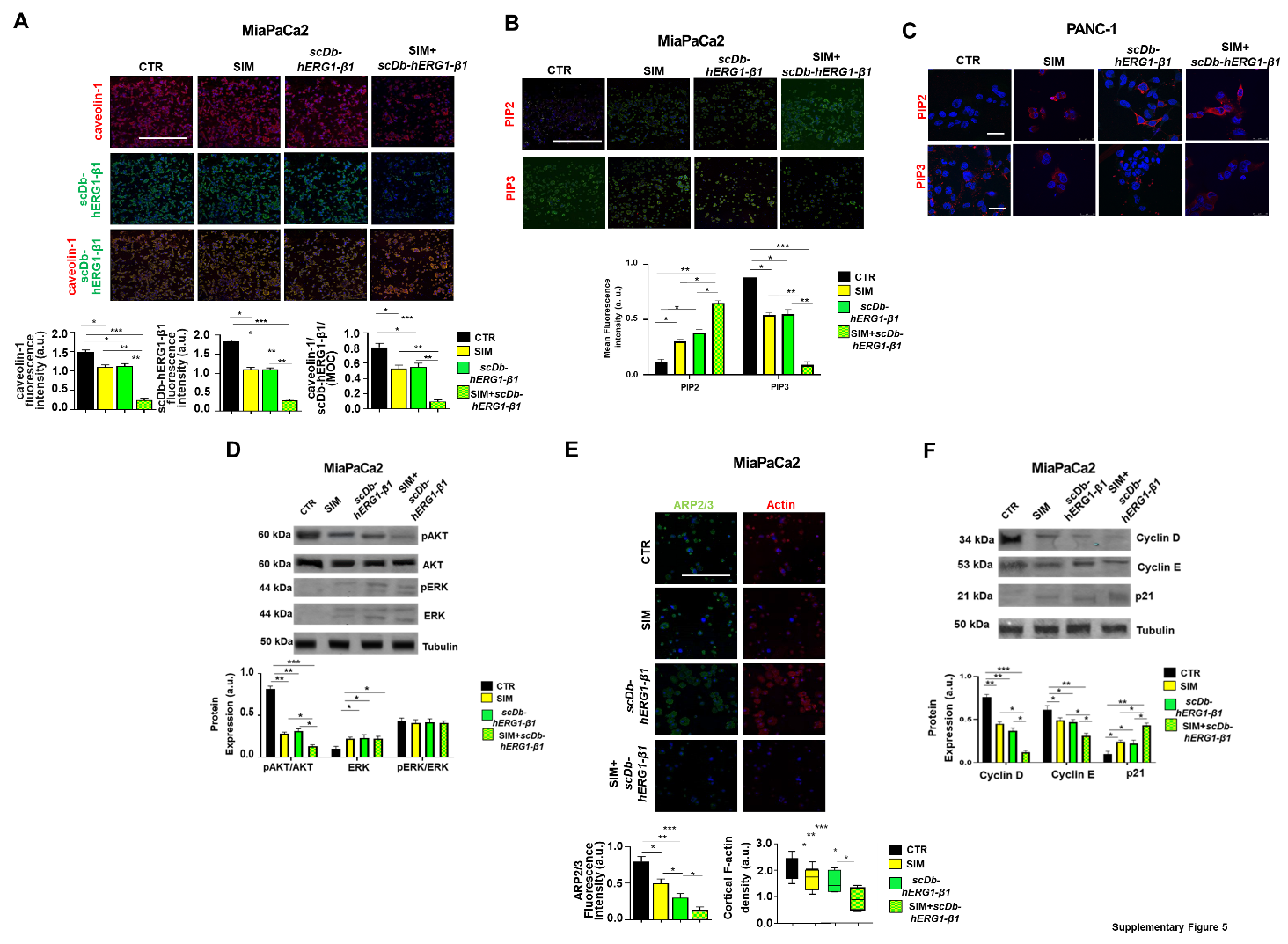


**Supplementary Figure 5. (A)** IF performed on MiaPaCa2 cells following 90 min adhesion onto FN with or without treatment with SIM (4.7μM), scDb-hERG1-β1 (20µg/ml) and their combination. Representative images (scale bar: 100 μm) of caveolin-1 staining, scDb-hERG1-β1 staining and colocalization between caveolin-1 and scDb-hERG1-β1 are on the top, while quantitative analyses and MOC are reported in the graphs on the bottom. a.u.= arbitrary units. At least 20 cells (in 3 different fields) per condition from three independent experiments (*n*=3) were analyzed. All data are presented as mean values ± s.e.m. **(B)** IF performed on MiaPaCa2 cells untreated (CTR) or treated with SIM (4.7μM), scDb-hERG1-β1 (20µg/ml) and their combination, seeded on FN for 90 min. Representative images of PIP2 (top panels) and PIP3 (bottom panels) (scale bar: 100 μm) are on the top, while quantitative analyses (Mean fluorescence intensity) are reported in the graph on the bottom. At least 20 cells (in 3 different fields) per condition from three independent experiments (*n*=3) were analyzed. All data are presented as mean values ± s.e.m. **(C)** Lower magnification images of PIP2 (top panels) and PIP3 (bottom panels) of IF (reported in figure 5C) performed on PANC-1 cells untreated (CTR) or treated with SIM (4.7μM), scDb-hERG1-β1 (20µg/ml) and their combination, seeded on FN for 90 min. (scale bar: 25 μm). At least 20 cells (in 3 different fields) per condition from three independent experiments (*n*=3) were analyzed. All data are presented as mean values ± s.e.m. **(D)** Representative blot (top) and densitometric analysis (bottom) of phospho-Akt and phospho-ERK levels in MiaPaCa2 cells untreated (CTR) or treated with SIM (4.7µM), scDb-hERG1-β1 (20µg/ml) and their combination, seeded on FN for 90 min. Data are presented as mean values ± s.e.m. (*n*=3). a.u. = arbitrary units. Membranes were probed with anti-pAkt Thr308, anti-Akt Thr308, ERK1/2 (pERK1/2) (Thr202/tyr204) and anti-total ERK1/2 antibodies. **(E)** IF on MiaPaCa2 cells stained with anti-ARP2/3 antibody and cortical F-actin after treatment with SIM (4.7μM), scDb-hERG1-β1 (20µg/ml) and their combination onto FN for 90 mins. At least 20 cells (in 3 different fields) per condition from three independent experiments (*n*=3) were analyzed. (scale bar: 100 μm). Quantification graphs of ARP2/3 fluorescent intensity and cortical F-actin density were reported in the bottom panels. Data are presented as mean values ± s.e.m. **(F)** Representative blot (top) and densitometric analysis (bottom) of Cyclin D, Cyclin E and p21 in MiaPaCa2 cells untreated (CTR) or treated with scDb-hERG1-β1 (20ug/ml), SIM (4.7µM) and their combination, seeded on FN for 90 min. Data are presented as mean values ± s.e.m. (*n*=3). a.u. = arbitrary units. Membranes were probed with anti-Cyclin D, anti-Cyclin E and anti-p21 antibodies. *P < 0.05; **P < 0.01, and ***P < 0.001 (One-Way ANOVA). CTR: control; MβCD: Methyl-β-cyclodextrin; SIM: simvastatin; MOC: Mander’s Overlapping Coefficient.


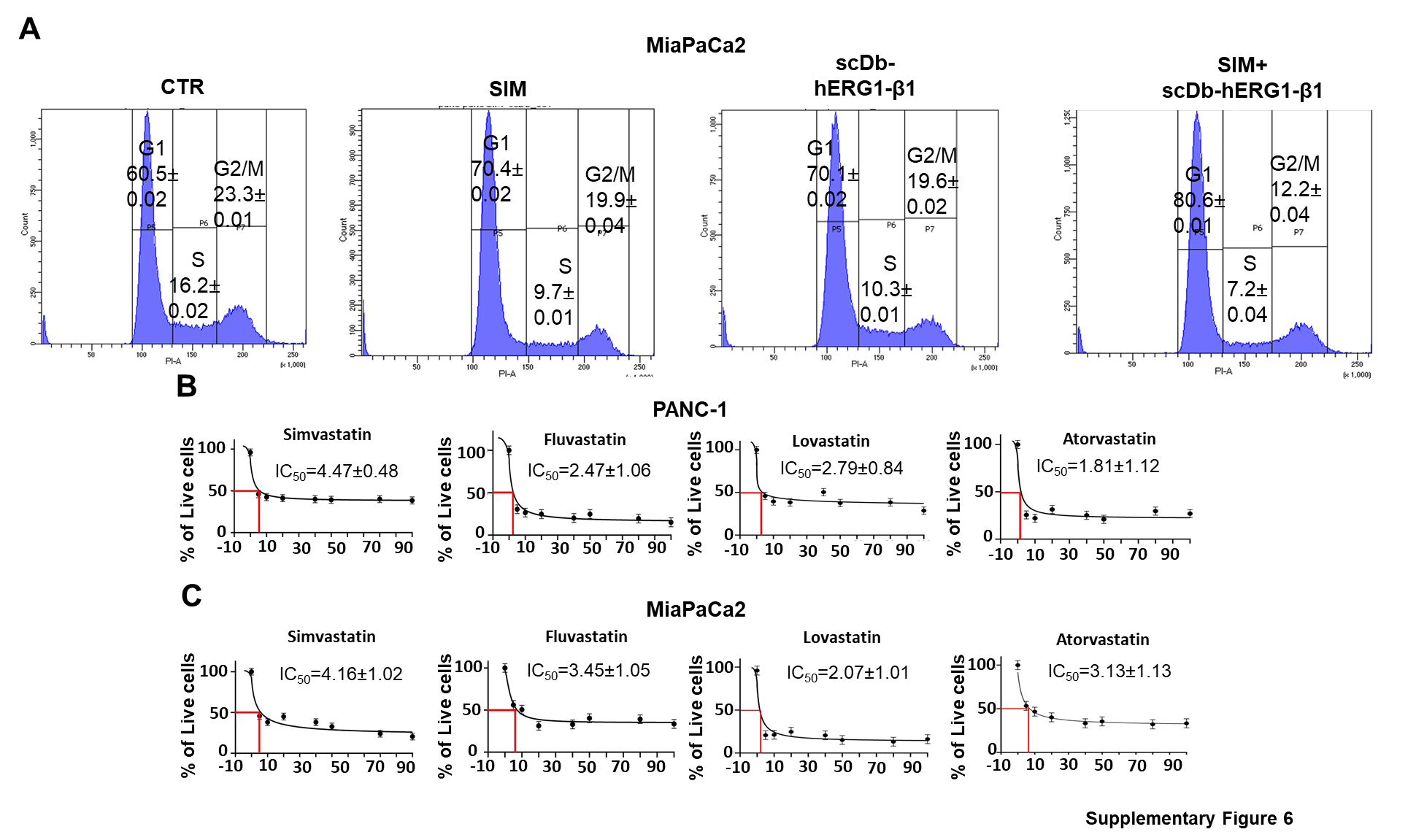


**Supplementary Figure 6. (A)** Facs plots of cell cycle of MiaPaCa2 cells treated with with IC_50_ of SIM, scDb-hERG1-β1 and IC_50_ of SIM + scDb-hERG1-β1 for 24h. SIM vs CRT: p=0.005 (G1); p=0.032 (S); p=0.037 (G2/M). scDb-hERG1-β1 vs CTR: p=0.004 (G1); p=0.038 (S); p=0.039 (G2/M). SIM+ scDb-hERG1-β1 vs CRT: p=0.0005 (G1); p=0.001 (S); p=0.0002 (G2/M). Data are presented as mean values ± s.e.m. (*n*=3). **(B)** IC_50_ values and curves of SIM, FLUVA, LOVA, ATOR of PANC-1 cells. Data are presented as mean values ± s.e.m. (*n*=3). **(C)** IC_50_ values and curves of SIM, FLUVA, LOVA, ATOR of MiaPaCa2 cells. Data are presented as mean values ± s.e.m. (*n*=3). CTR: control; SIM: Simvastatin; FLUVA: Fluvastatin; LOVA: Lovastatin; ATOR: Atorvastatin.


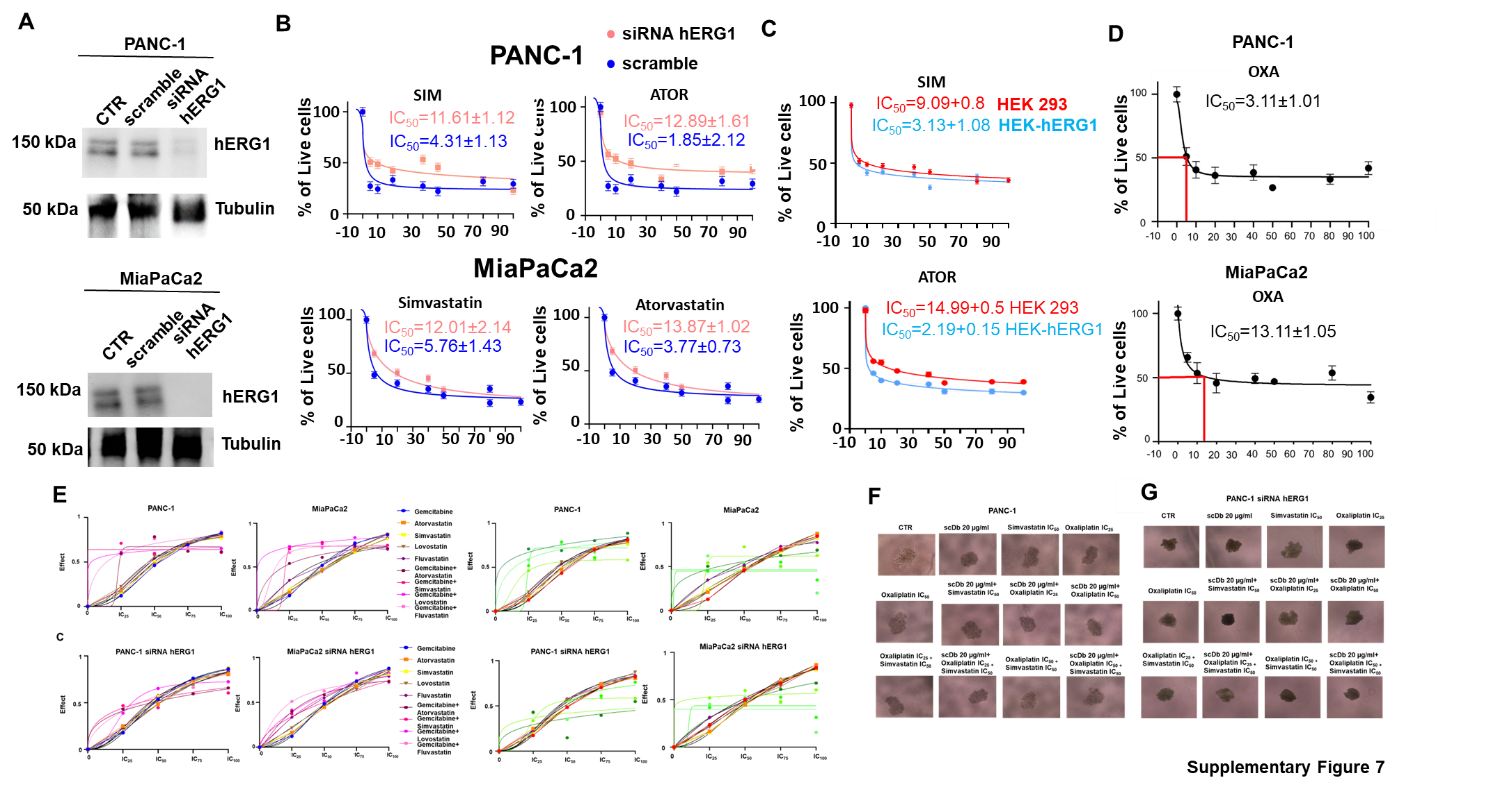


**Supplementary Figure 7. (A)** Representative membrane of hERG1 and Tubulin in hERG1 silenced PANC-1 (top panel) and MiaPaCa2 (bottom panel) cells. **(B)** IC_50_ values and curves of SIM and ATOR of hERG1 silenced PANC-1 (top panel) and MiaPaCa2 (bottom panel) cells. Data are presented as mean values ± s.e.m. (*n*=3). **(C)** IC_50_ values and curves of SIM (top panel) and ATOR (bottom panel) of HEK 293 and HEK-hERG1 cells. Data are presented as mean values ± s.e.m. (*n*=3). **(D)** IC_50_ values and curves of Oxaliplatin of PANC-1 (top panel) and MiaPaCa2 (bottom panel) cells. Data are presented as mean values ± s.e.m. (*n*=3). **(E)** Top panel: combination Index curves of PANC-1 and MiaPaCa2 cells CTR and treated with statins, GEM (left panel) and OXA (right panel). We combined the IC25, IC50, IC75 and IC100 of the two chemotherapeutic drugs with the IC25, IC50, IC75 and IC100 of the four statins, deriving these concentrations from the IC50 values shown in Table 1A. The Combination Index for all treatments was then calculated. Data are presented as mean values ± s.e.m. (*n*=3). Bottom panel: combination Index curves of PANC-1 and MiaPaCa2 hERG1 silenced cells CTR and treated with statins, GEM (left panel) and OXA (right panel). Data are presented as mean values ± s.e.m. (*n*=3). **(F)** Representative images of PANC-1 3D cells treated with SIM (IC50 value), OXA (IC50 and IC25) and scDb-hERG1-β1 (20 µg/ml) for 48h. **(G)** Representative images of 3D hERG1 silenced PANC-1 cells treated with (IC50 value), OXA (IC50 and IC25) and scDb-hERG1-β1 (20 µg/ml) for 48h. CTR: control. IC: inhibitory concentration. SIM: Simvastatin; FLUVA: Fluvastatin; LOVA: Lovastatin; ATOR: Atorvastatin. GEM: gemcitabine; OXA: oxaliplatin.
