# Supplementary material for: Targeting the hERG1/β1 integrin complex in lipid rafts potentiates statins anti-cancer activity in pancreatic cancer
Source: Cell Death Discov. 2025 Feb 3;11:39. doi: 10.1038/s41420-025-02321-2 (PMC11790905; doi:10.1038/s41420-025-02321-2)
Supplement: Supplementary file 2 — Supplemental material file with original uncropped membranes [file 41420_2025_2321_MOESM2_ESM.pdf]

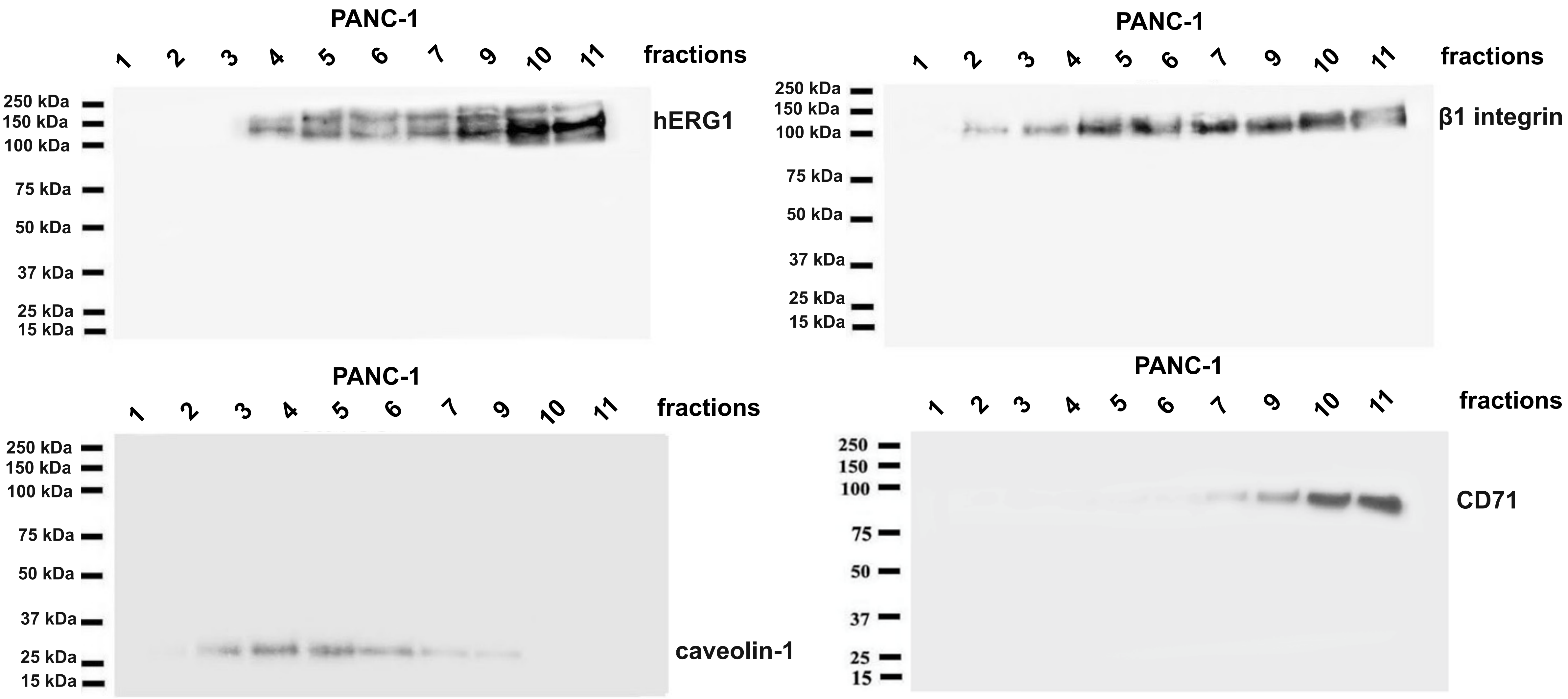

Source data Figure 1A

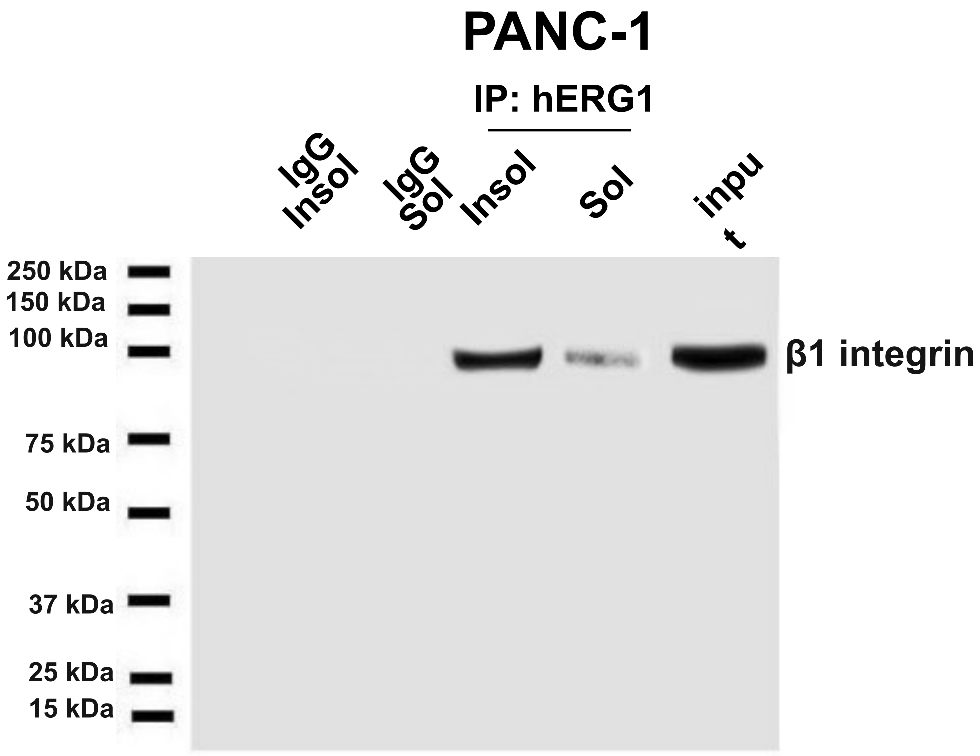

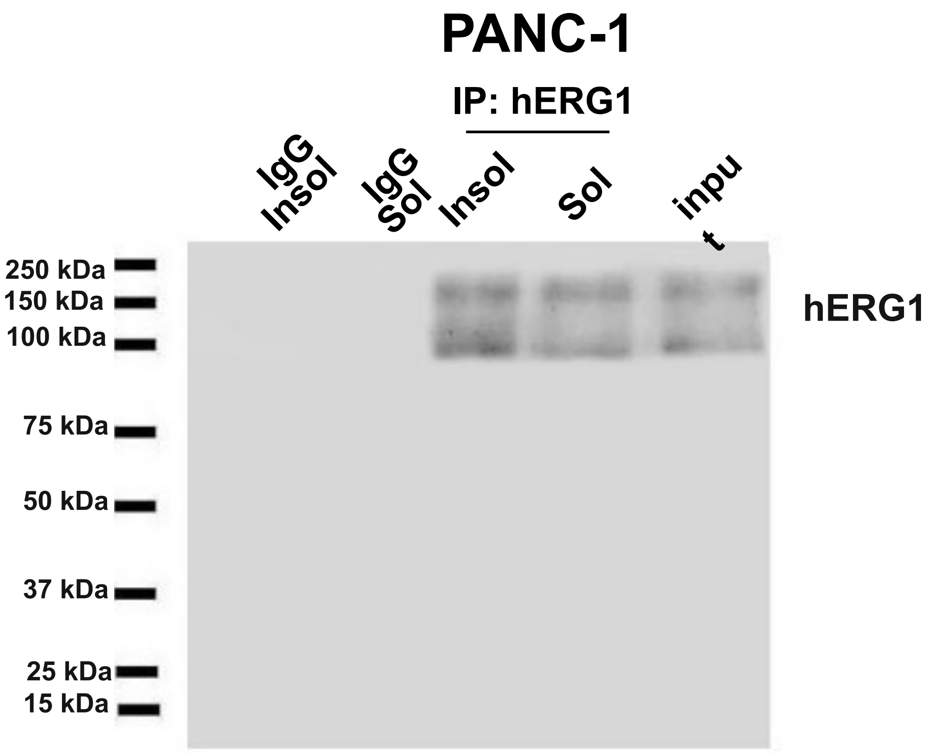

Source data Figure 1C

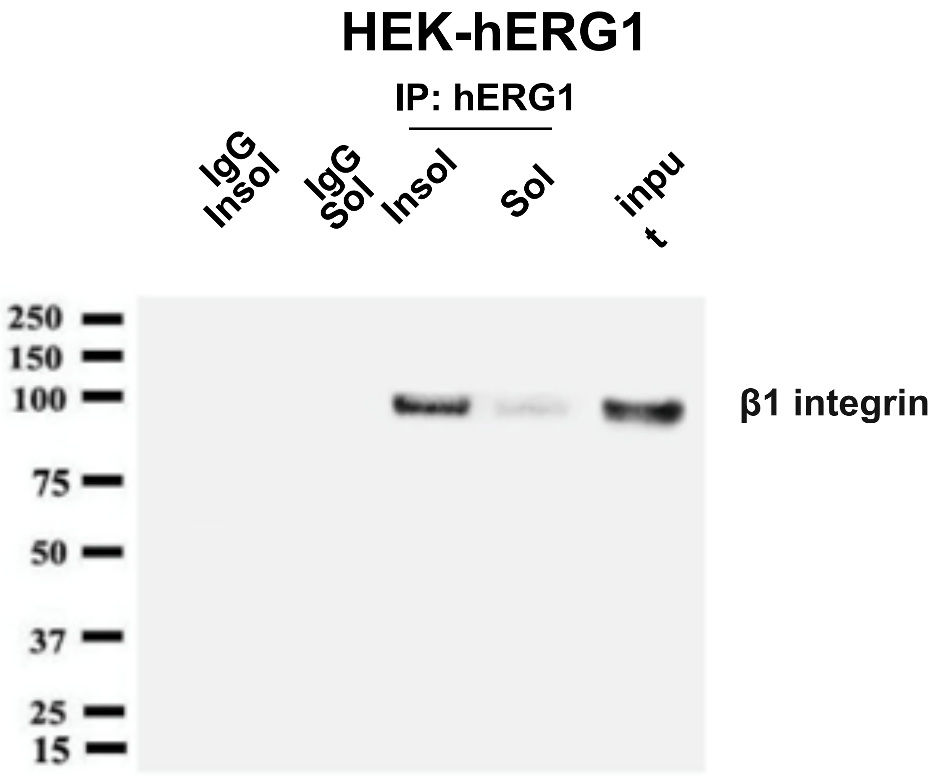

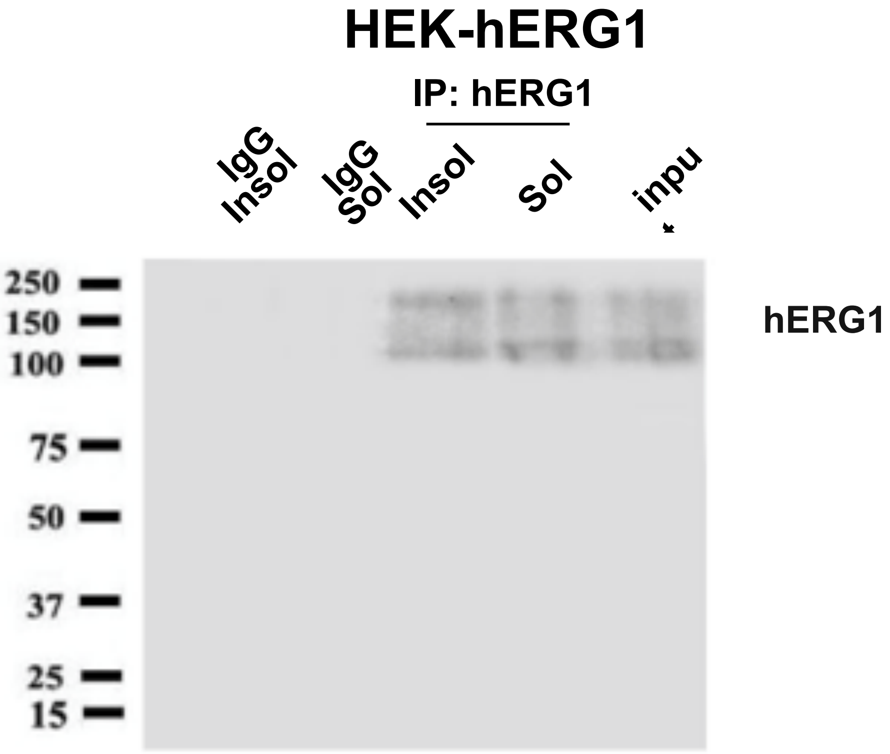

Source data Figure 1D

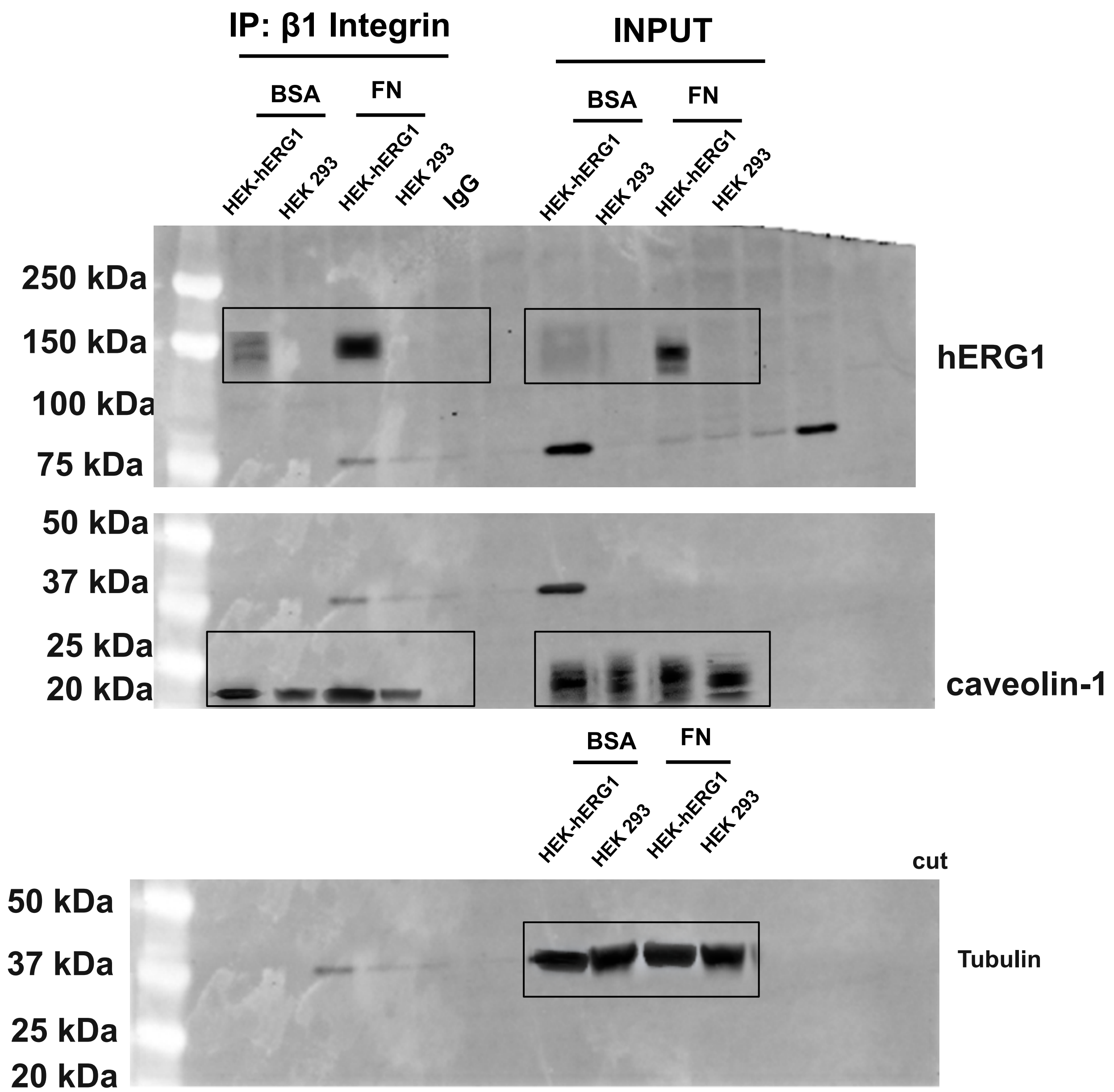

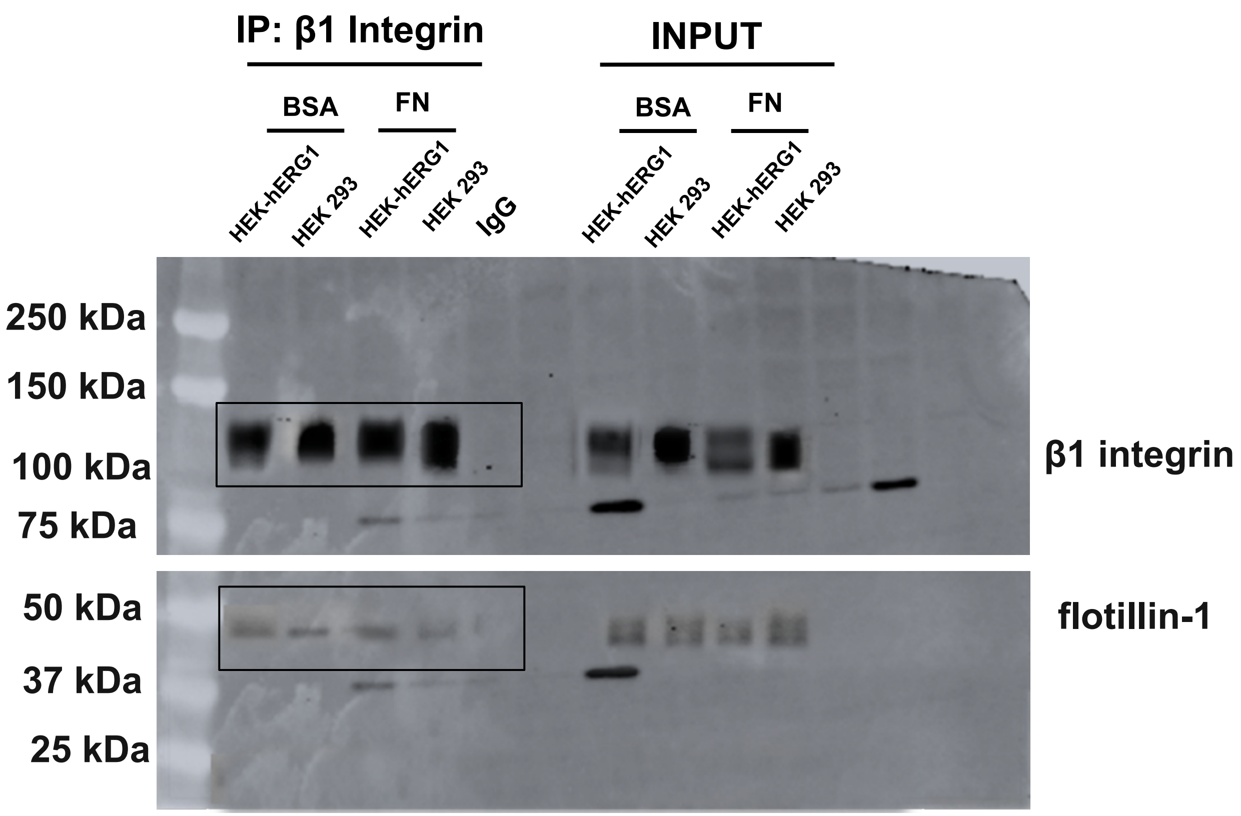

Source data Figure 1E

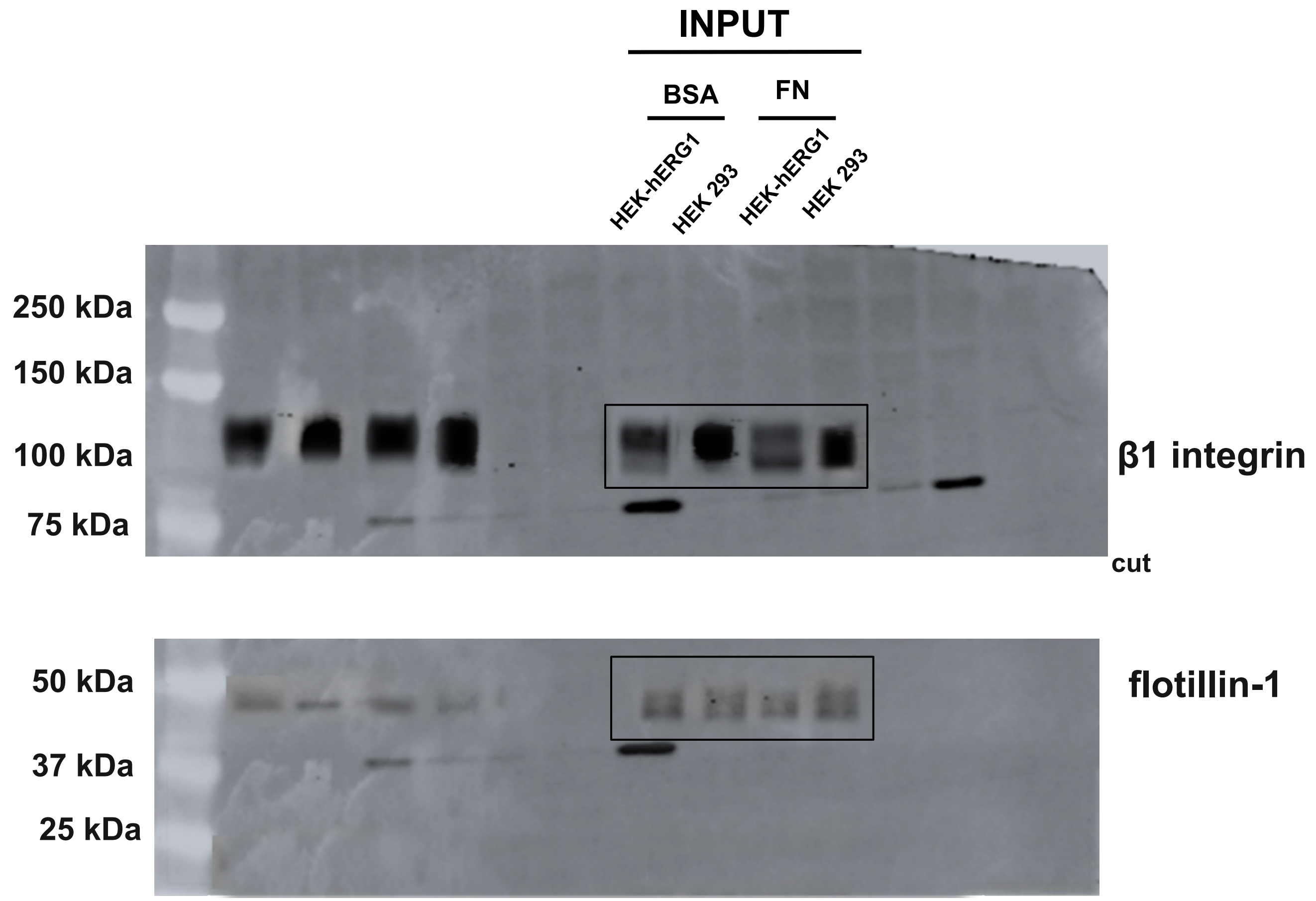

Source data supplementary Figure 1A

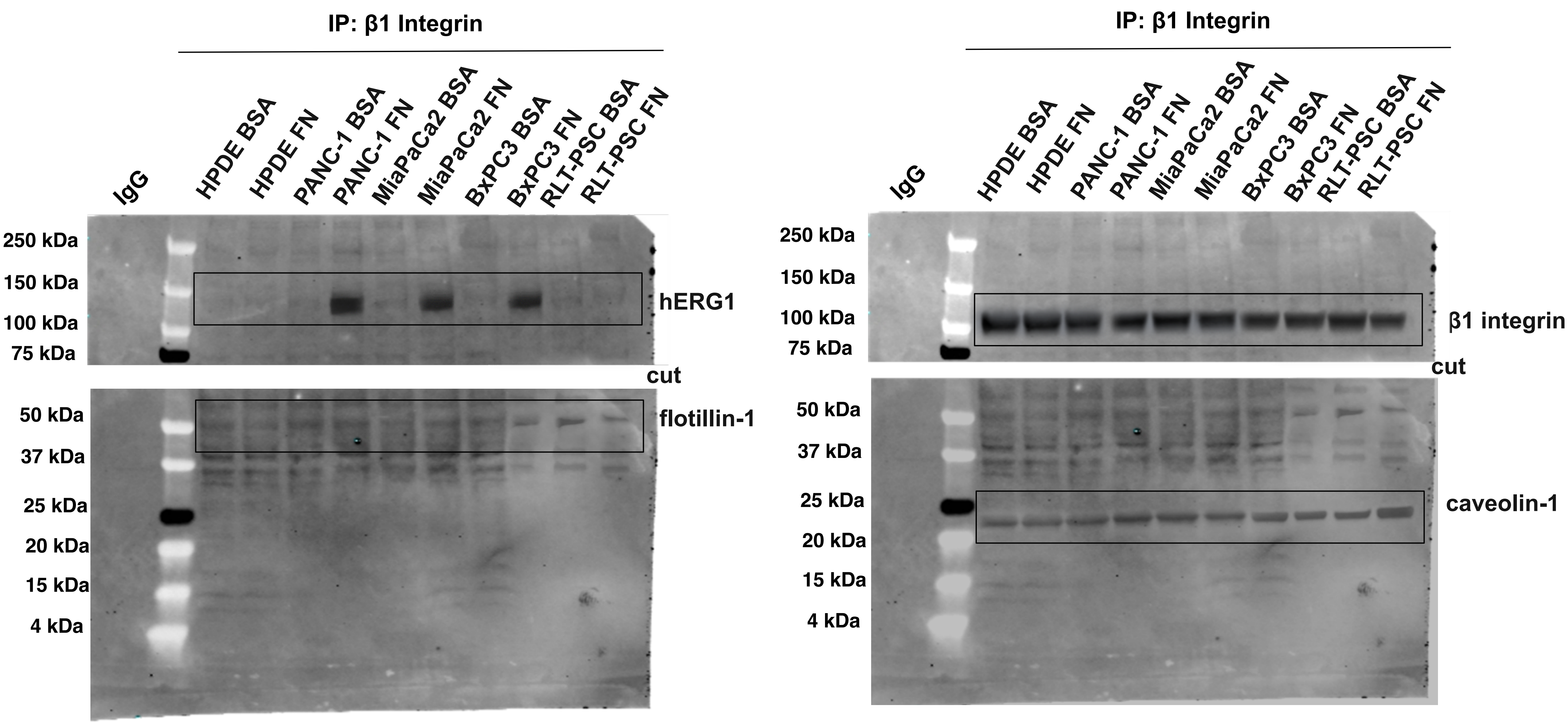

Source data IP Figure 1F

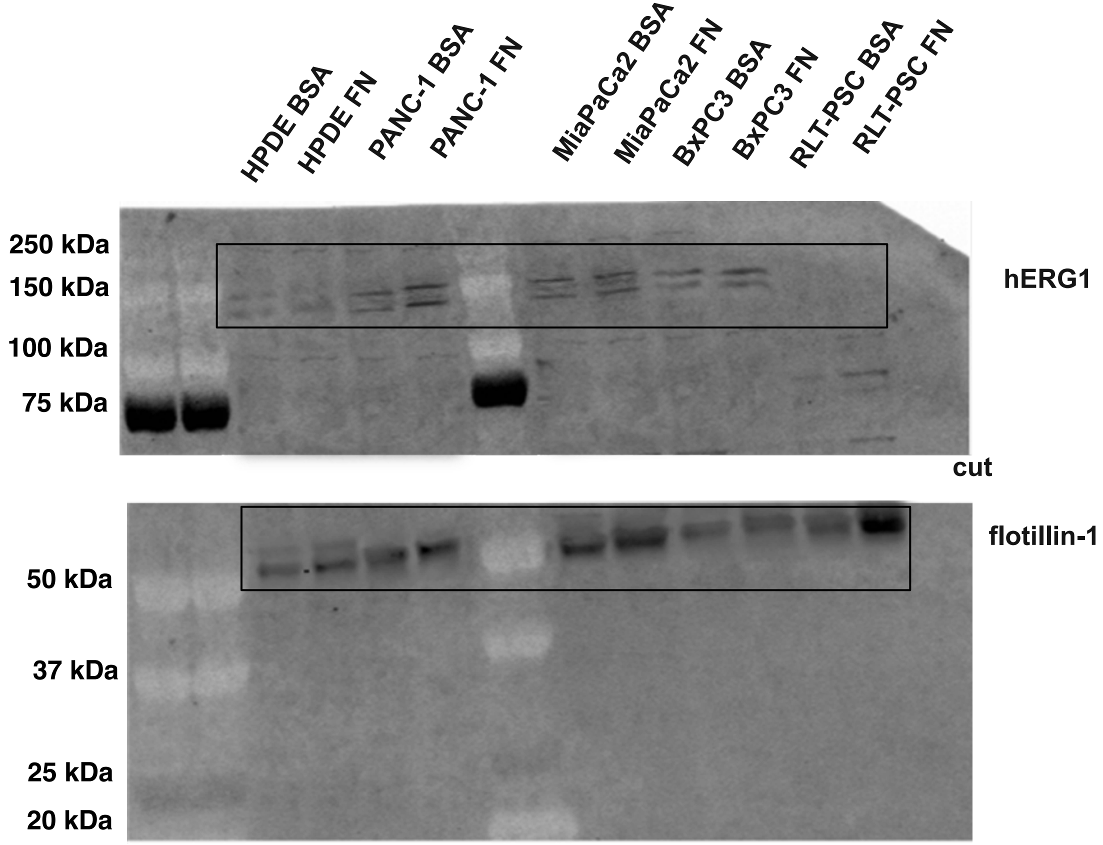

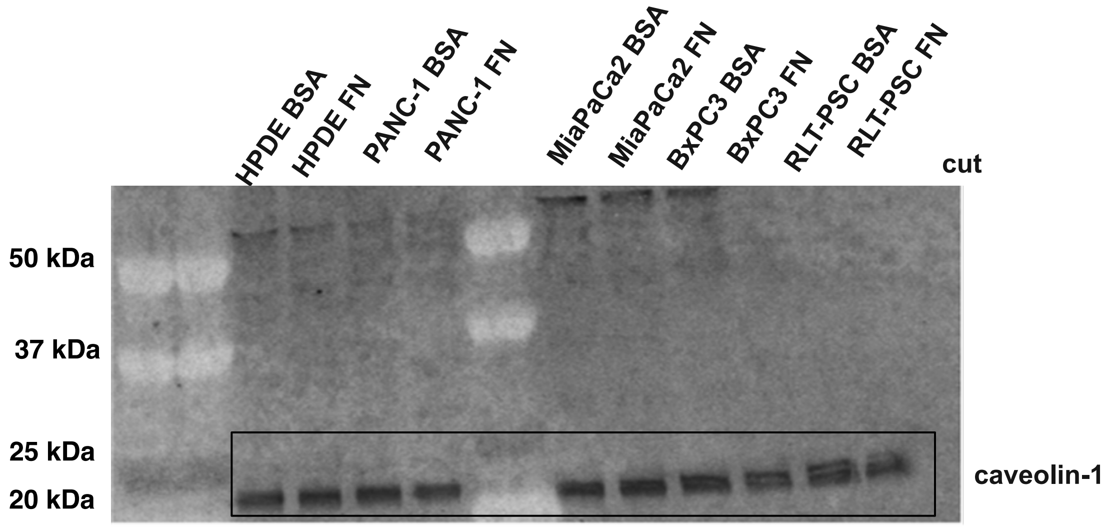

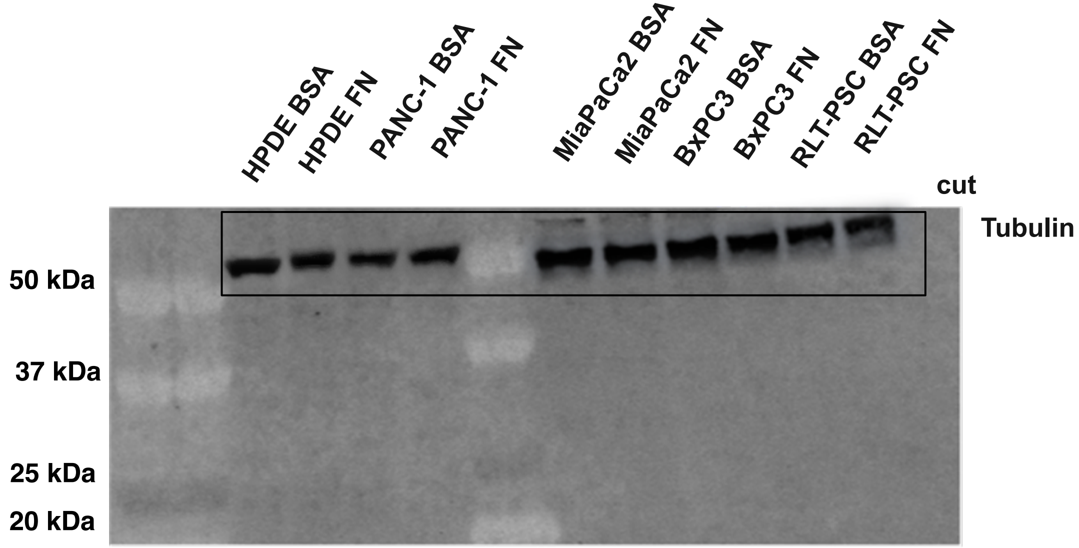

Source data input Figure 1f

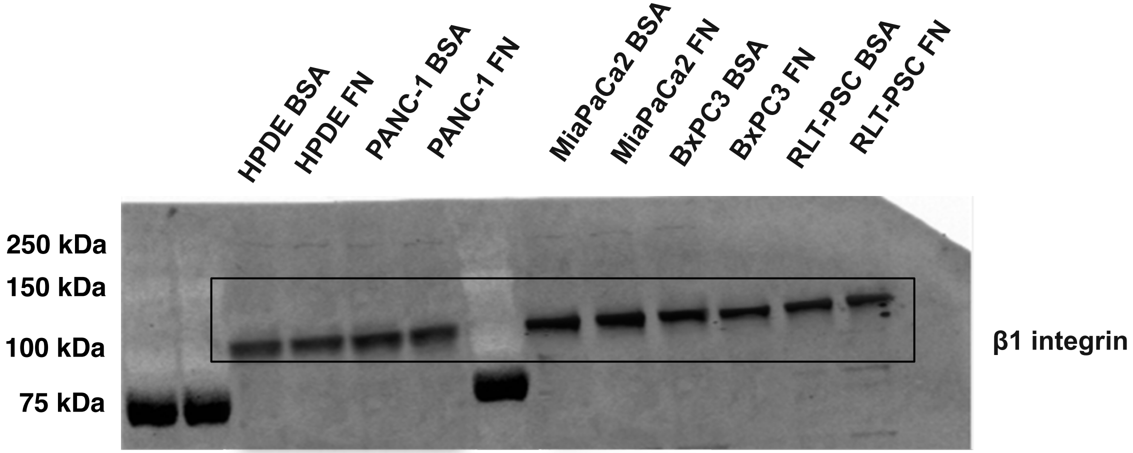

Source data supplementary Figure 1B

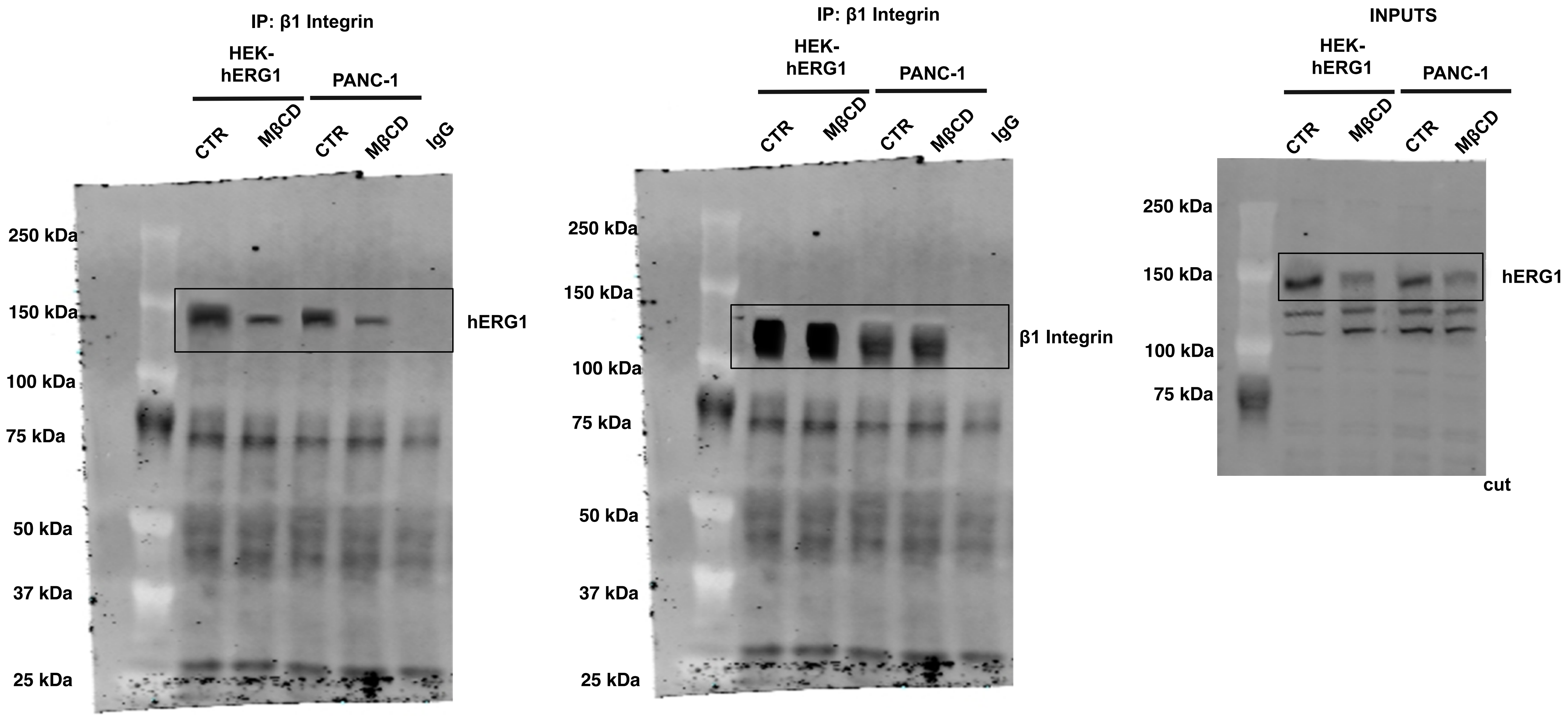

Source data Figure 3B

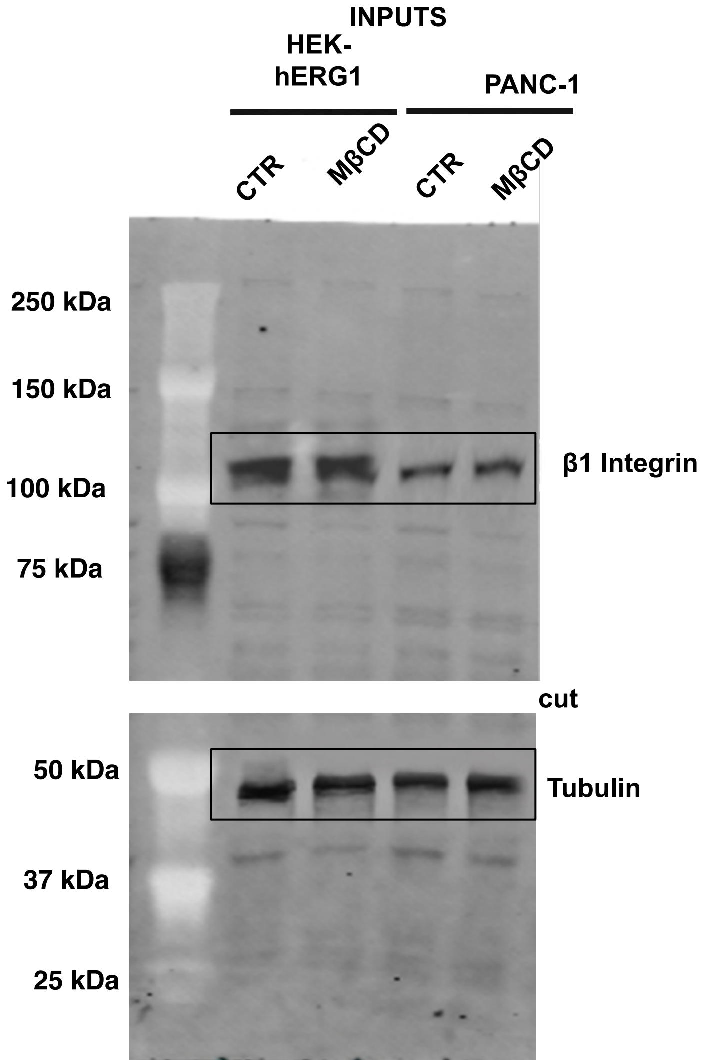

Source data supplementary Figure 3A

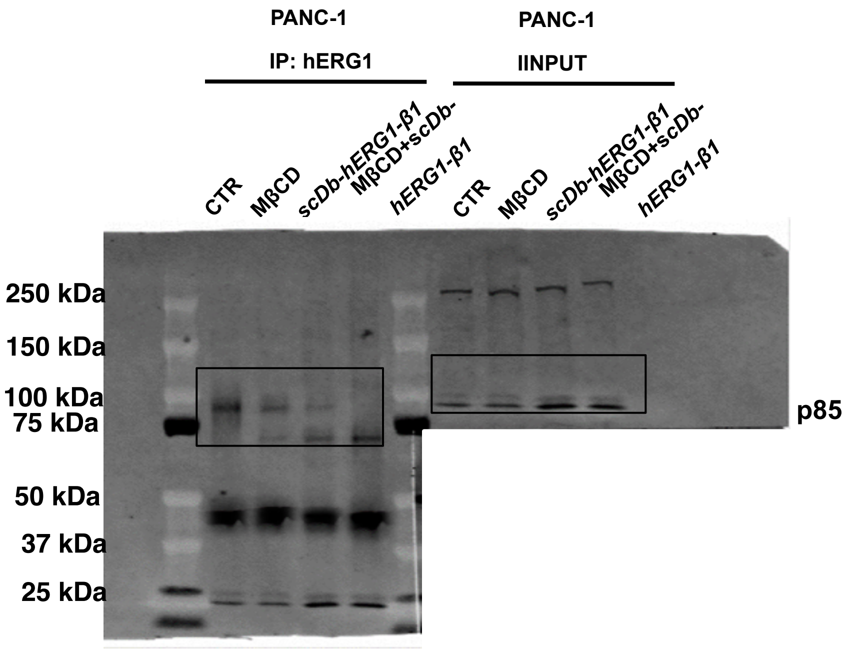

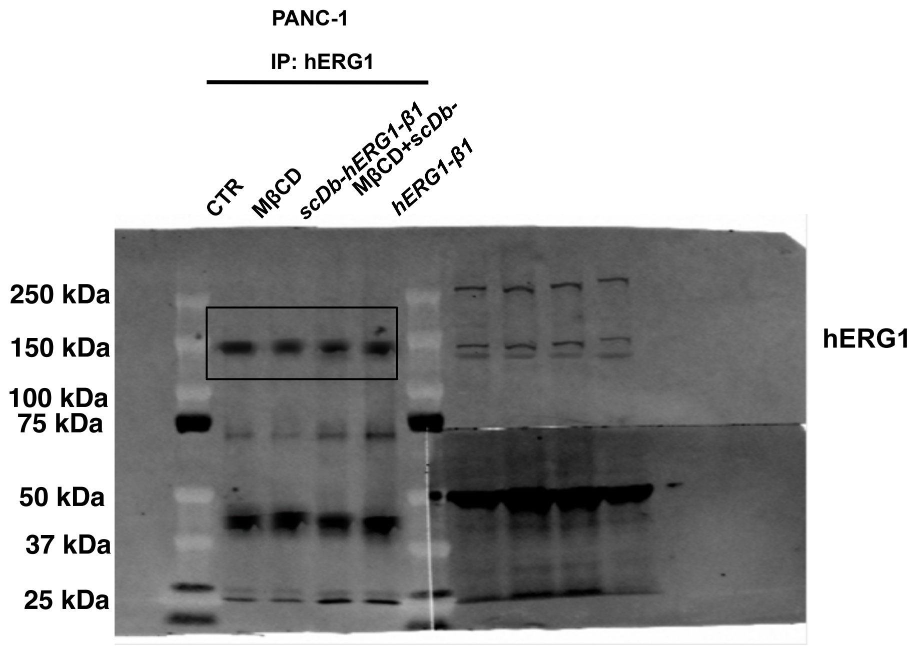

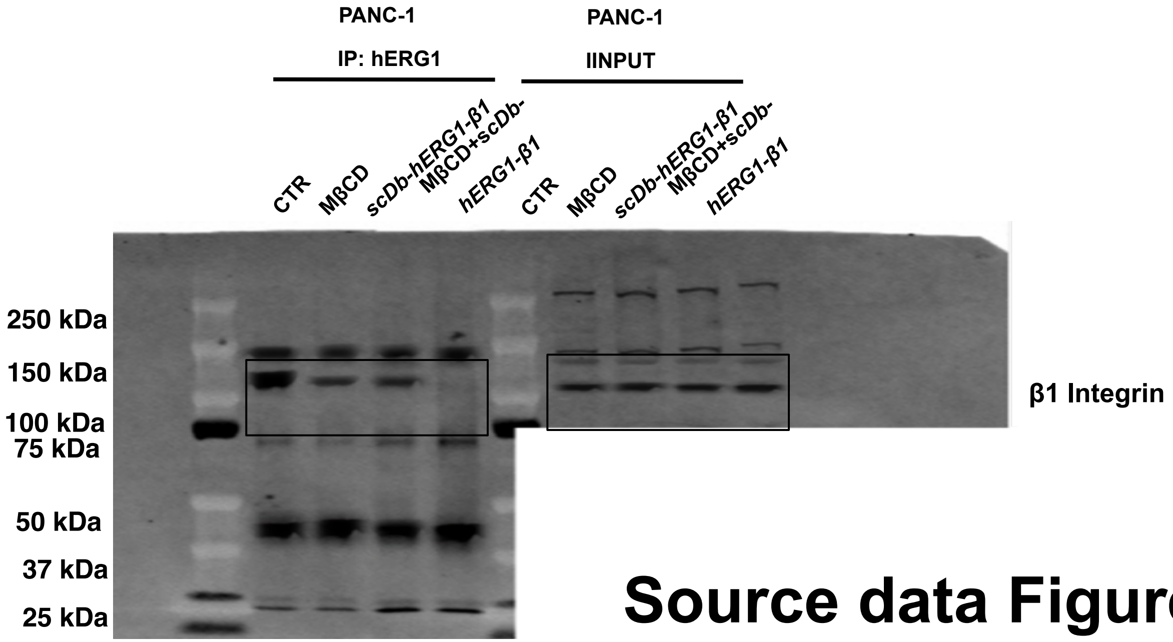

Source data Figure 3F

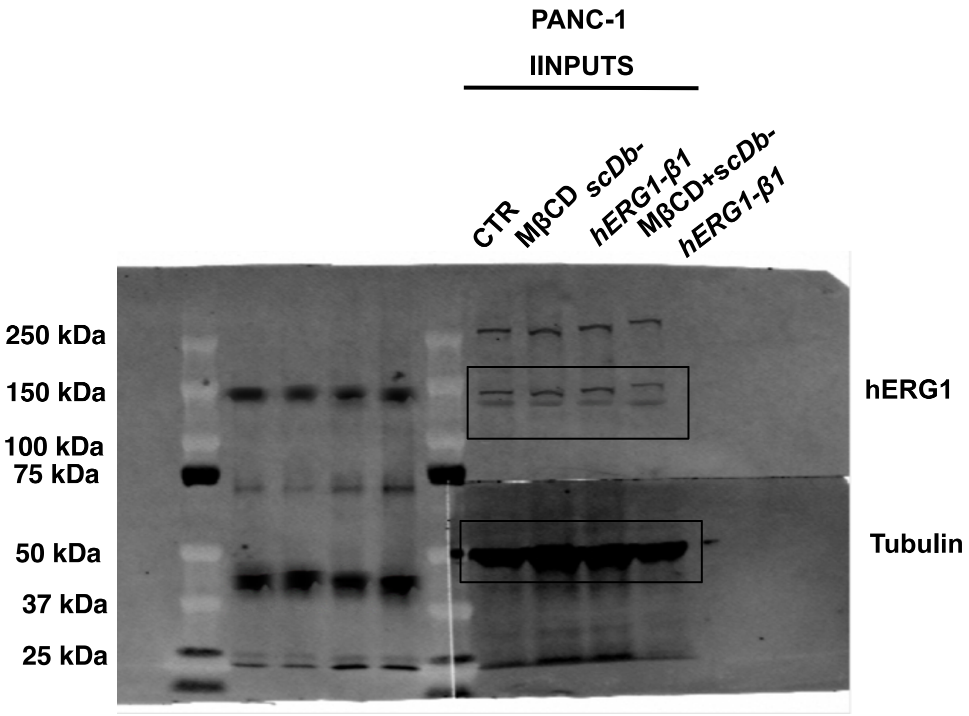

Source data supplementary Figure 3E

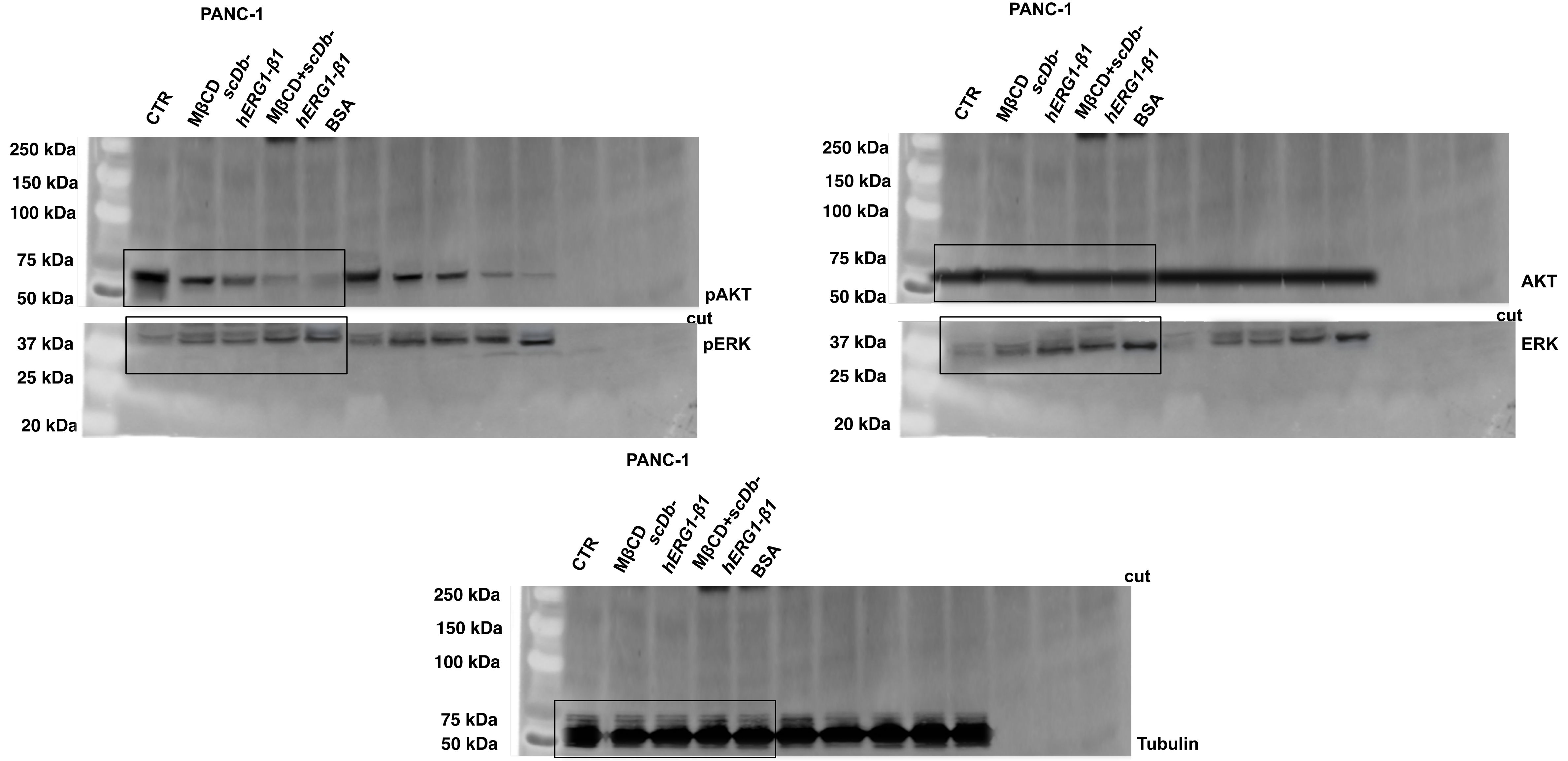

Source data Figure 3H

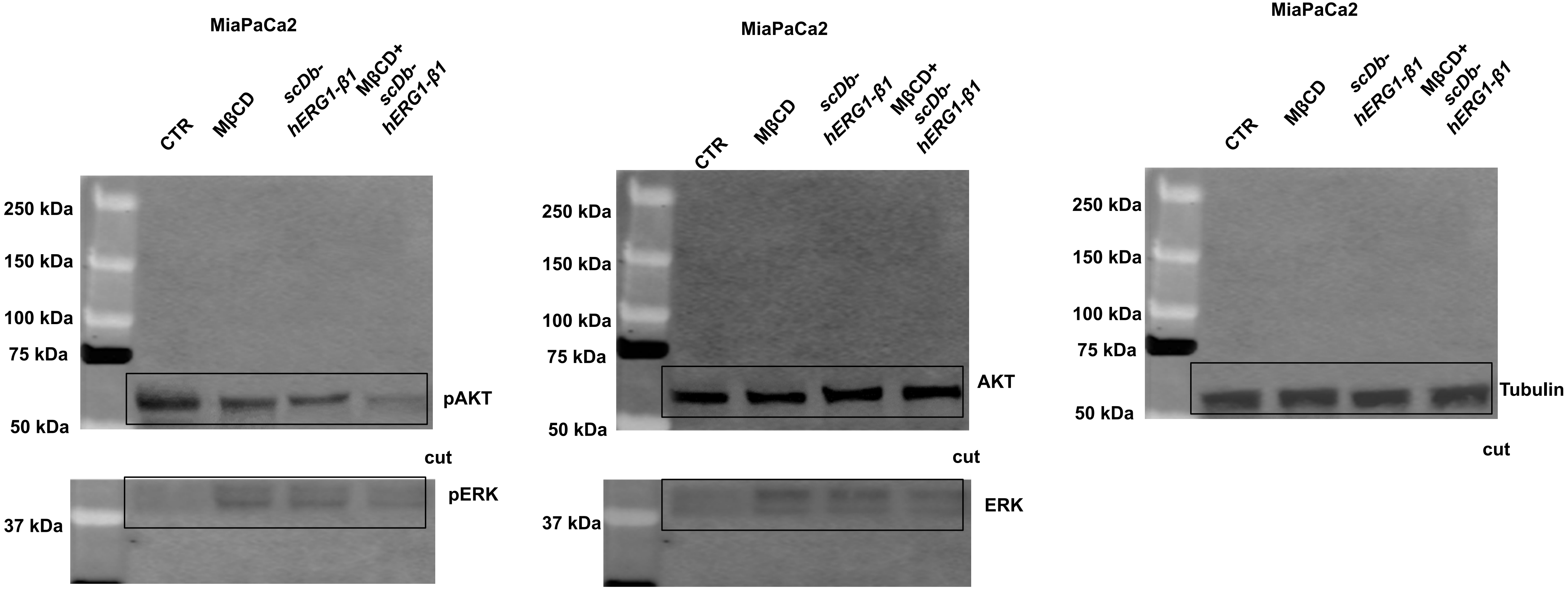

Source data Supplementary Figure 3H

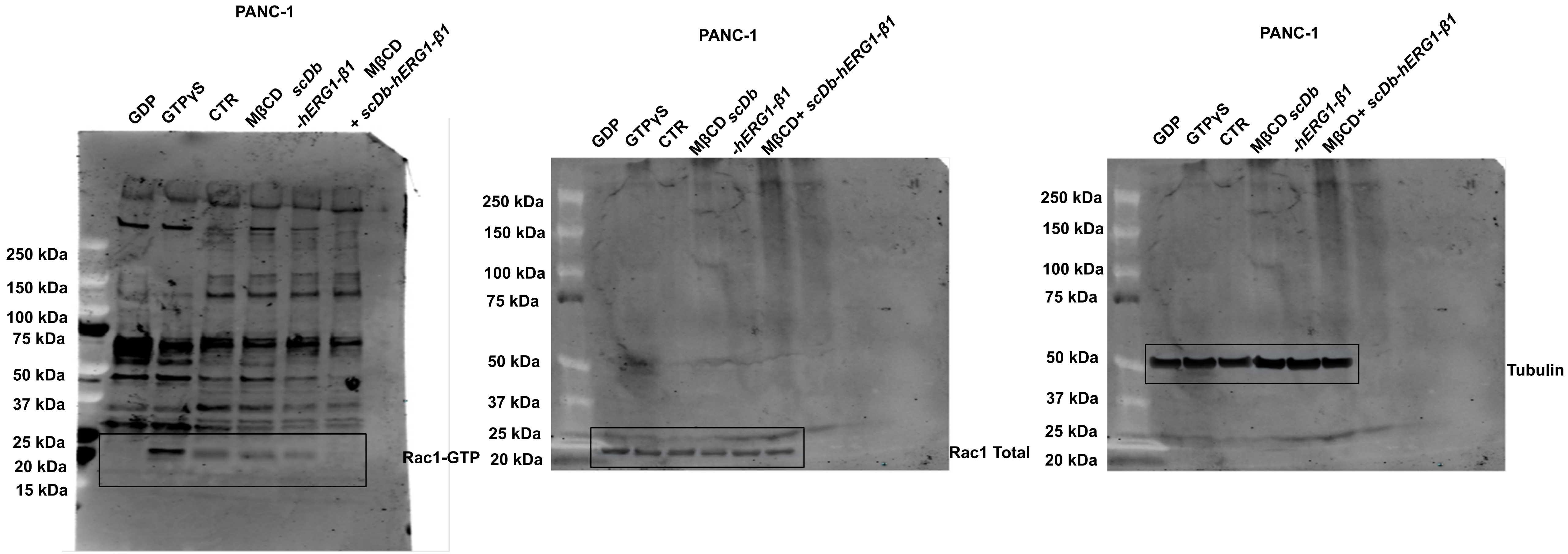

Source data Figure 4A

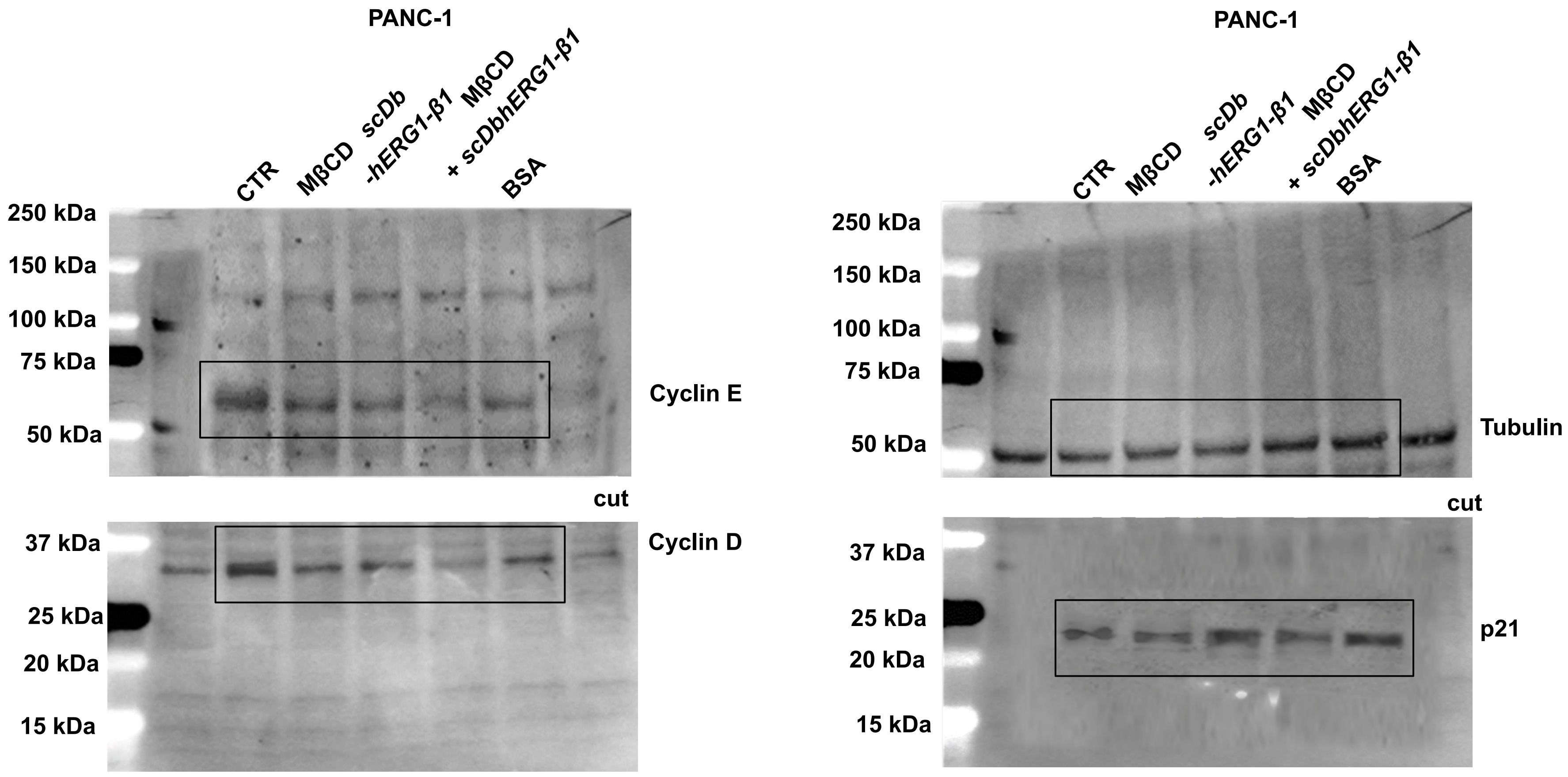

Source data Figure 4D

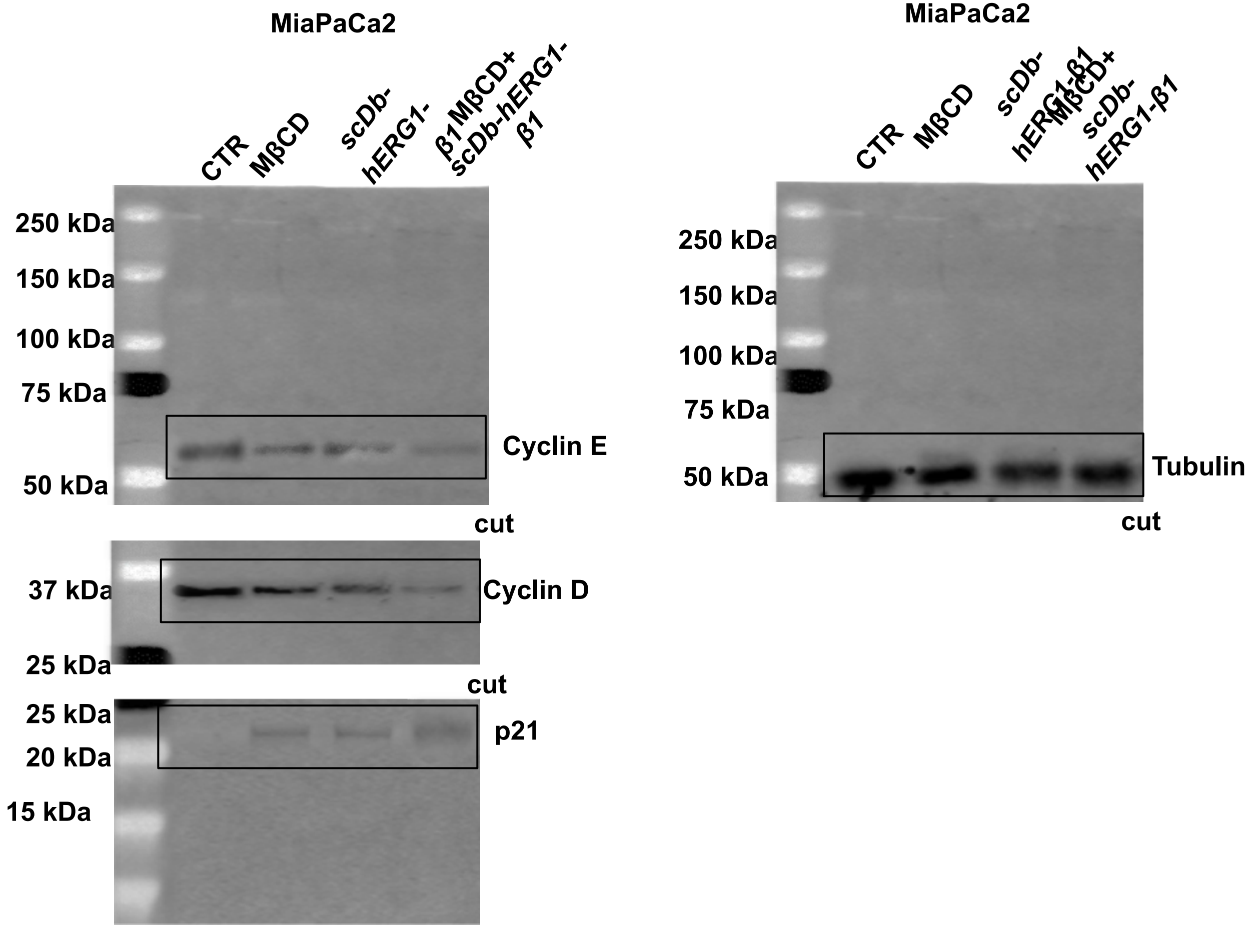

Source data Supplementary Figure 4C

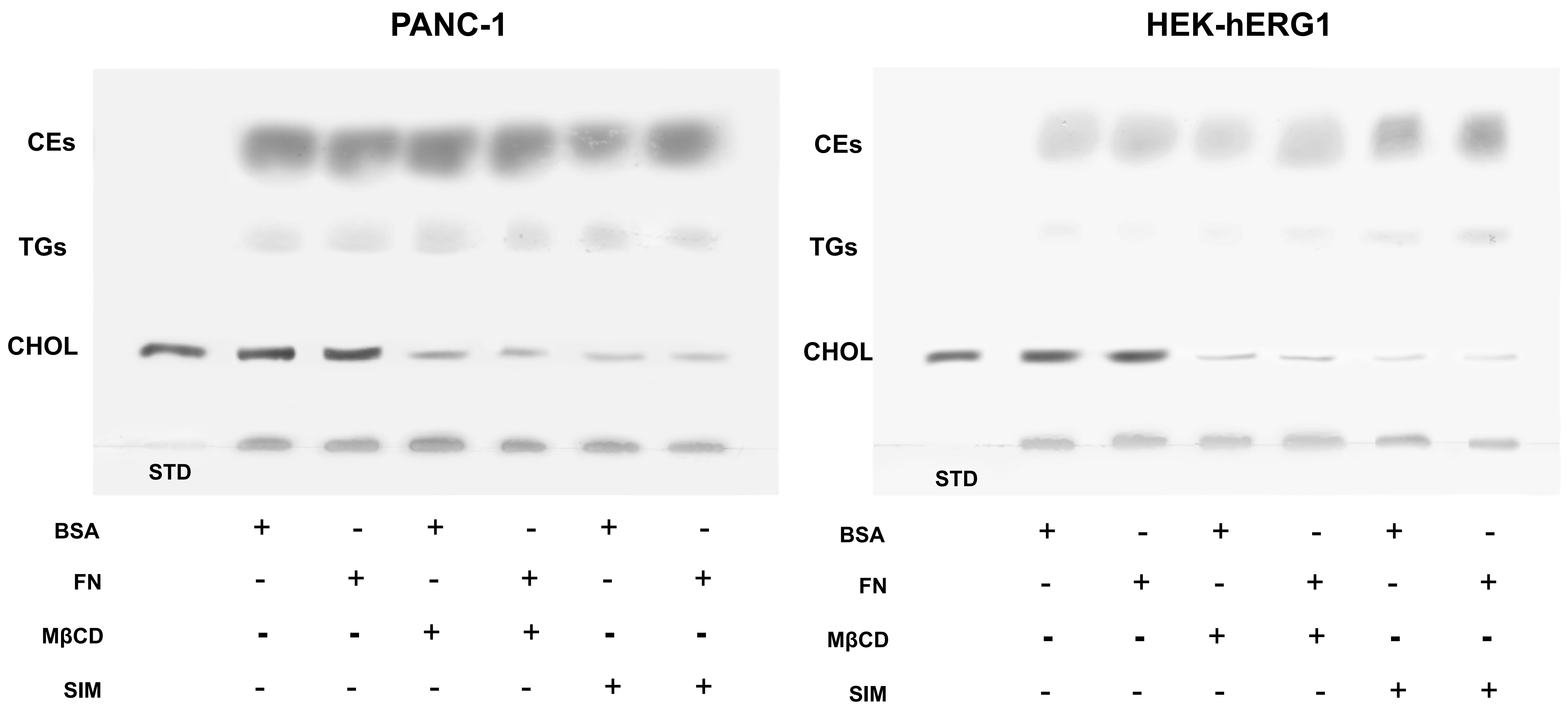

Source data Figure 5A

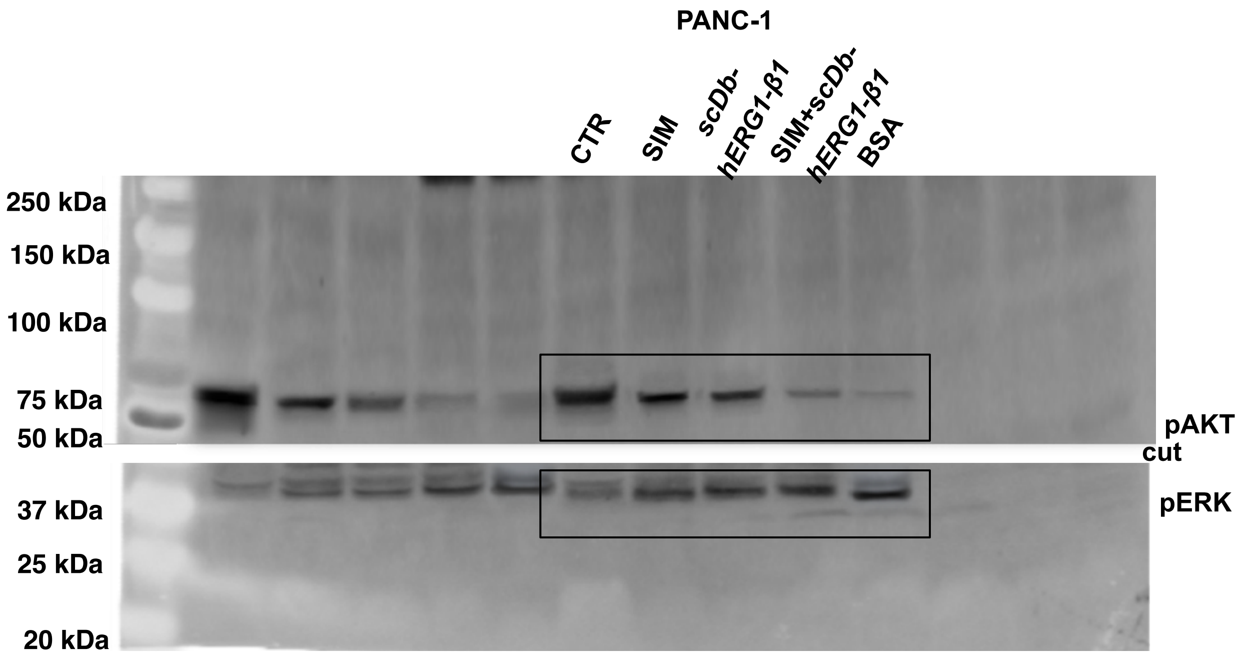

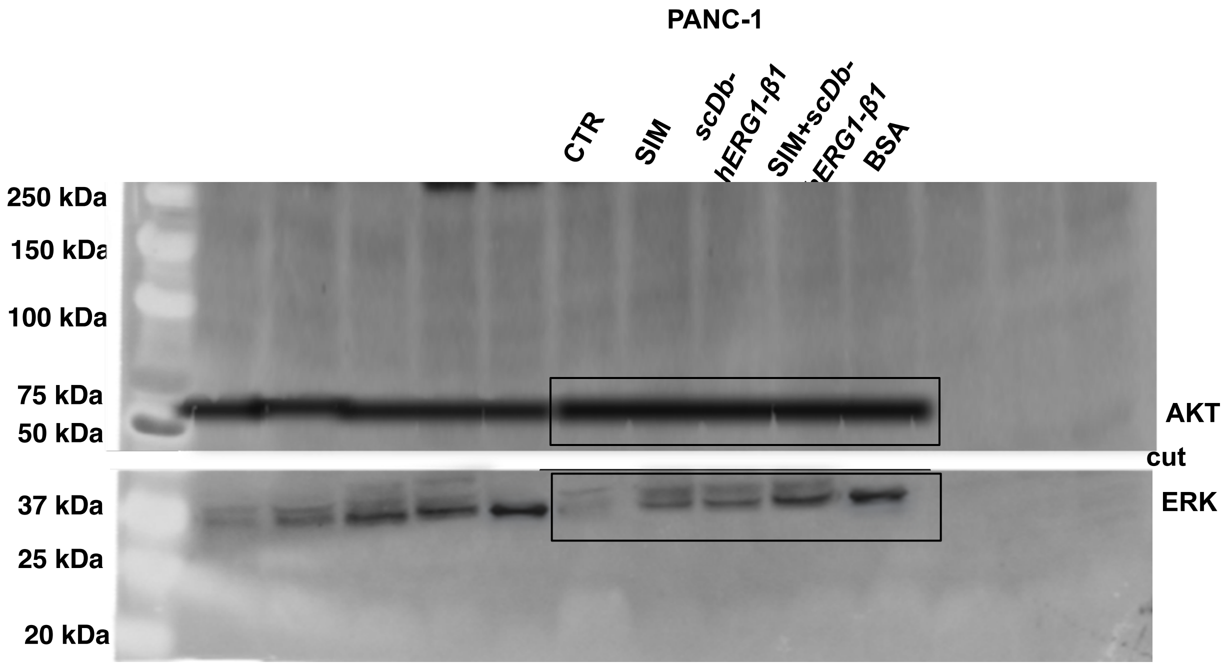

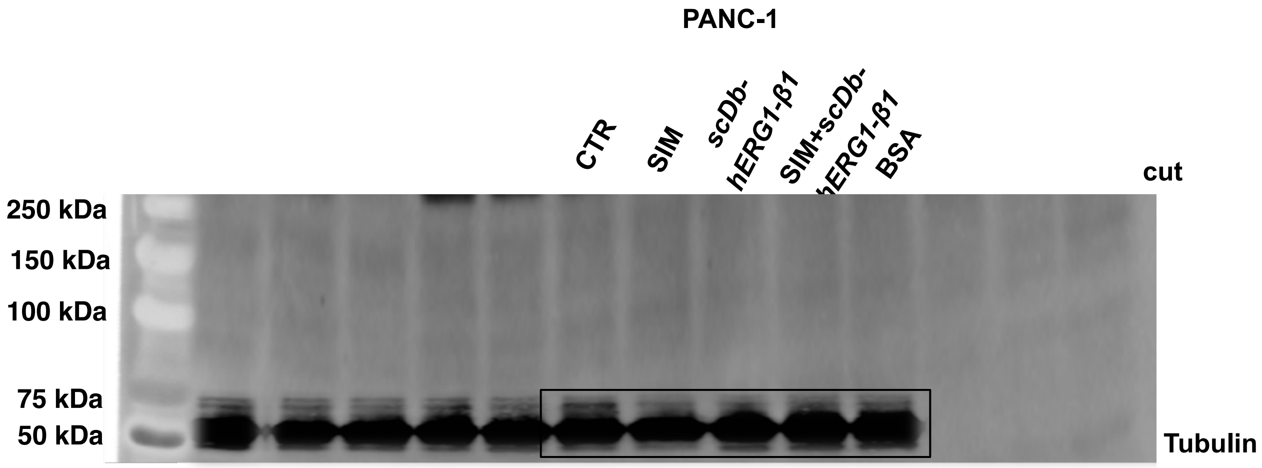

Source data Figure 5D

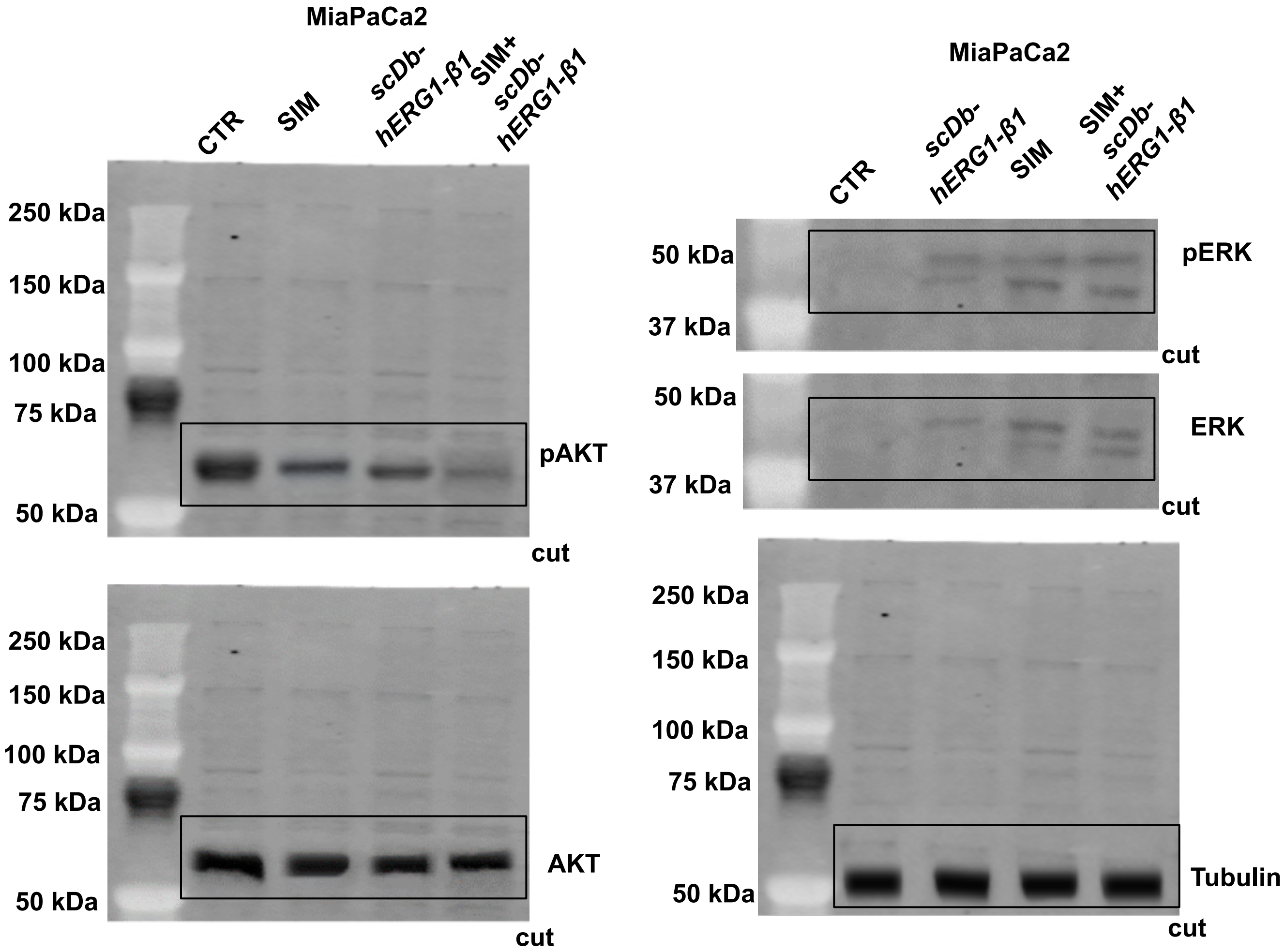

Source data Supplementary Figure 5D

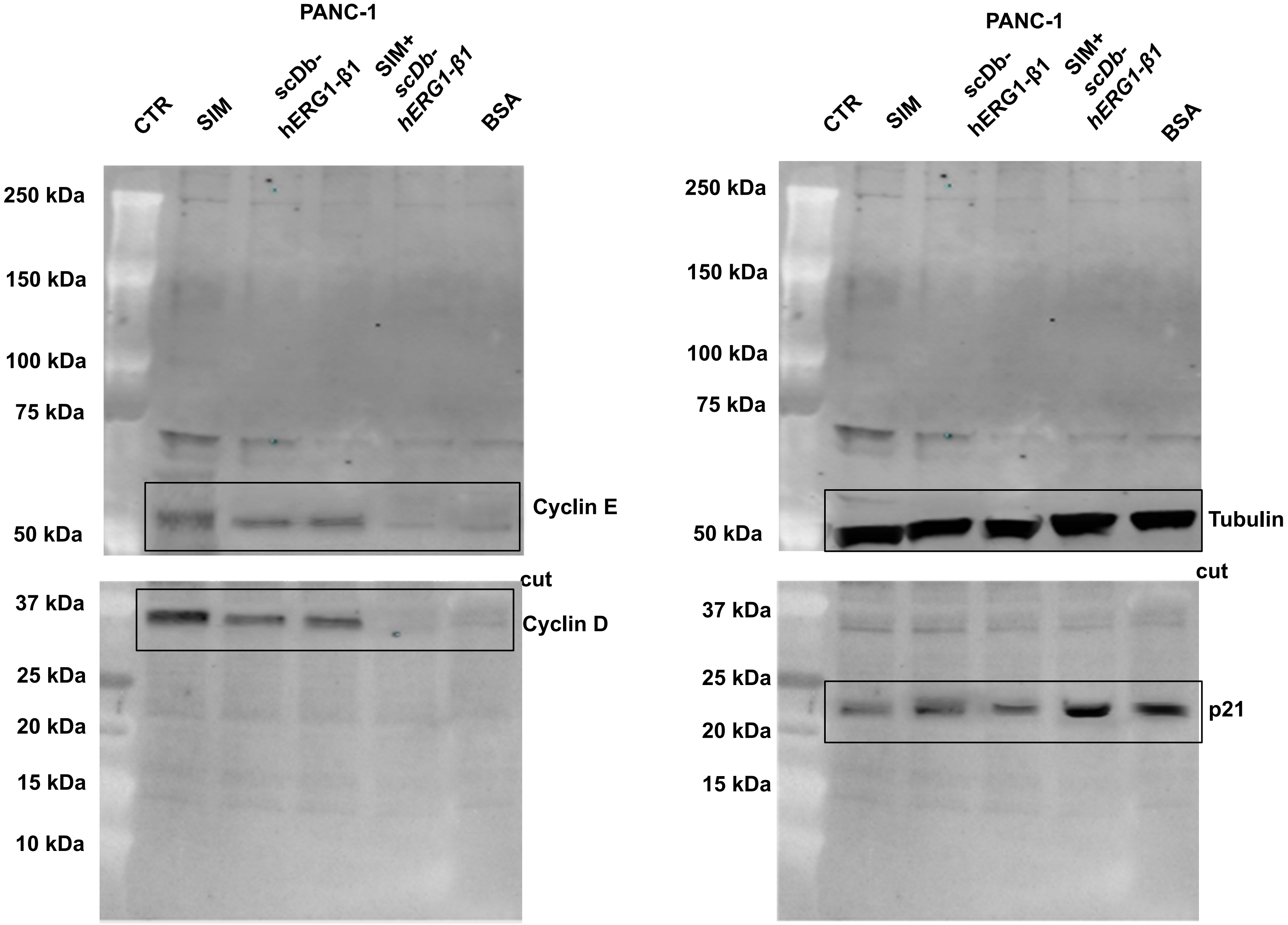

Source data Figure 5F

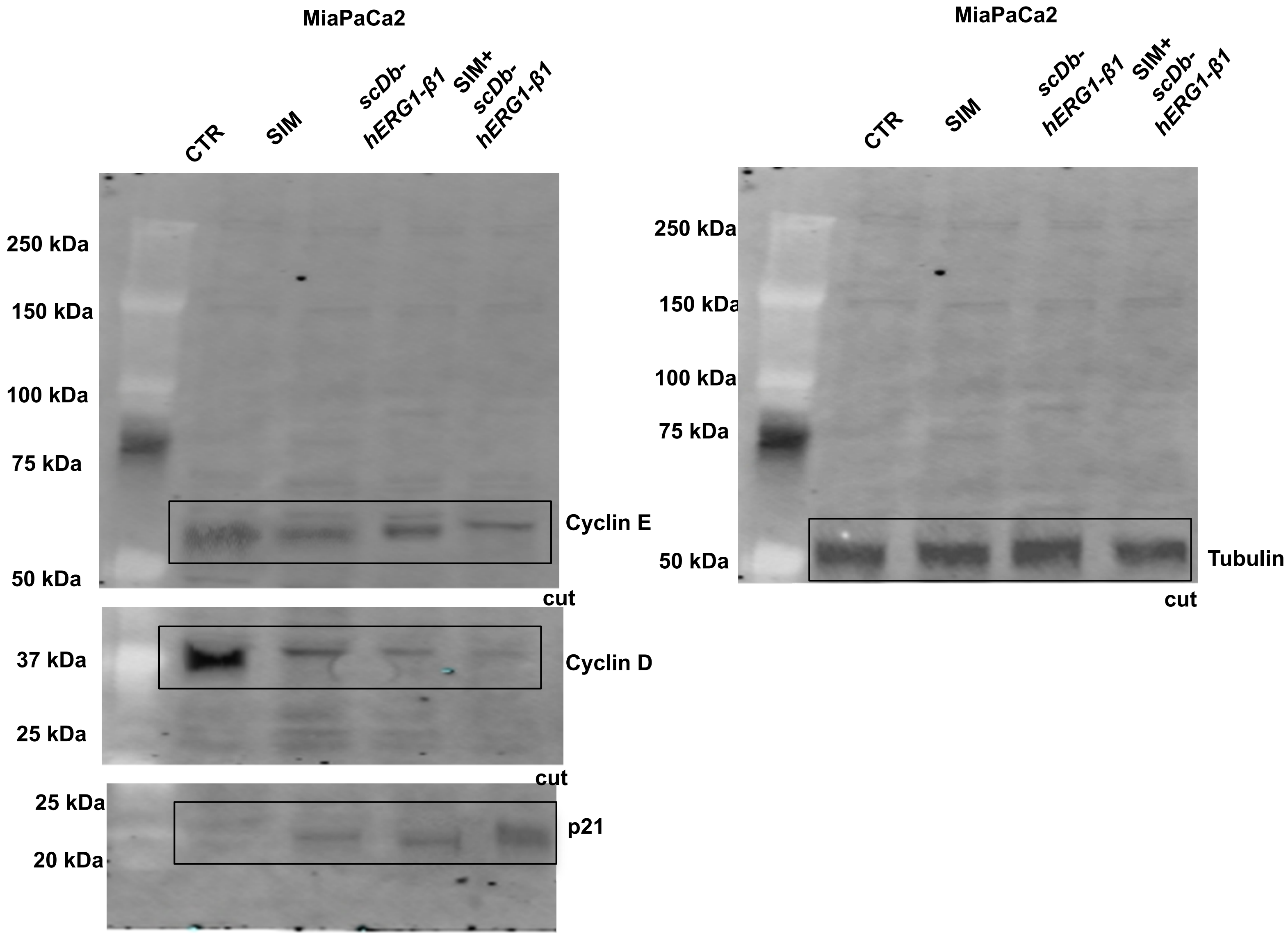

Source data Supplementary Figure 5F
