# Supplementary material for: Targeting the hERG1/β1 integrin complex in lipid rafts potentiates statins anti-cancer activity in pancreatic cancer
Source: Cell Death Discov. 2025 Feb 3;11:39. doi: 10.1038/s41420-025-02321-2 (PMC11790905; doi:10.1038/s41420-025-02321-2)
Supplement: Supplementary file 3 — Supplementary Table S1 [file 41420_2025_2321_MOESM3_ESM.docx]

| **PANC-1** | | | | **PANC-1 siRNA hERG1** | | | |
| --- | --- | --- | --- | --- | --- | --- | --- |
| **Dose** | **Gemcitabine**  **µM** | **Atorvastatin µM** | **CI Value** | **Dose** | **Gemcitabine**  **µM** | **Atorvastatin µM** | **CI Value** |
| **IC25** | 20.33 | 0,90 | 0.488 | **IC25** | 19.87 | 6.44 | 1.20 |
| **IC50** | 40.66 | 1,81 | 0.534 | **IC50** | 39.75 | 12.89 | 2.05 |
| **IC75** | 60.99 | 2,71 | 1.324 | **IC75** | 59.62 | 19.33 | 2.1 |
| **MAX** | 100 | 100 | 12.905 | **MAX** | 100 | 100 | 5.3 |
| **Dose** | **Gemcitabine µM** | **Simvastatin µM** | **CI Value** | **Dose** | **Gemcitabine µM** | **Simvastatin µM** | **CI Value** |
| **IC25** | 20.33 | 2.35 | 0.370 | **IC25** | 19.87 | 5.8 | 1.00 |
| **IC50** | 40.66 | 4.47 | 1.035 | **IC50** | 39.75 | 11.61 | 2.13 |
| **IC75** | 60.99 | 6.07 | 1.050 | **IC75** | 59.62 | 17.41 | 1.32 |
| **MAX** | 100 | 100 | 8.013 | **MAX** | 100 | 100 | 6.51 |
| **Dose** | **Gemcitabine µM** | **Lovastatin µM** | **CI Value** | **Dose** | **Gemcitabine µM** | **Lovastatin µM** | **CI Value** |
| **IC25** | 20.33 | 1,40 | 0.531 | **IC25** | 19.87 | 5.23 | 1.3 |
| **IC50** | 40.66 | 2.80 | 1.147 | **IC50** | 39.75 | 10.46 | 1.2 |
| **IC75** | 60.99 | 4.20 | 1.150 | **IC75** | 59.62 | 15.69 | 1.6 |
| **MAX** | 100 | 100 | 9.589 | **MAX** | 100 | 100 | 5.06 |
| **Dose** | **Gemcitabine µM** | **Fluvastatin µM** | **CI Value** | **Dose** | **Gemcitabine µM** | **Fluvastatin µM** | **CI Value** |
| **IC25** | 20.33 | 1.75 | 0.562 | **IC25** | 19.87 | 3.64 | 1.27 |
| **IC50** | 40.66 | 3.45 | 0.603 | **IC50** | 39.75 | 7.28 | 1.58 |
| **IC75** | 60.99 | 5.20 | 1.227 | **IC75** | 59.62 | 10.92 | 1.69 |
| **MAX** | 100 | 100 | 2.524 | **MAX** | 100 | 100 | 2.53 |
| **Dose** | **Oxaliplatin µM** | **Atorvastatin µM** | **CI Value** | **Dose** | **Oxaliplatin µM** | **Atorvastatin µM** | **CI Value** |
| **IC25** | 1,53 | 0,9 | 0.106 | **IC25** | 1.53 | 6.44 | 1.09 |
| **IC50** | 3.06 | 1,809 | 0.230 | **IC50** | 3.25 | 12.89 | 19.84 |
| **IC75** | 4.59 | 2,71 | 0.125 | **IC75** | 4.59 | 19.33 | 4.13 |
| **MAX** | 100 | 100 | 1.153 | **MAX** | 100 | 100 | 20.67 |
| **Dose** | **Oxaliplatin µM** | **Simvastatin µM** | **CI Value** | **Dose** | **Oxaliplatin µM** | **Simvastatin µM** | **CI Value** |
| **IC25** | 1,53 | 2.35 | 0.476 | **IC25** | 1.53 | 5.23 | 4.55 |
| **IC50** | 3.06 | 4.47 | 0.136 | **IC50** | 3.25 | 10.46 | 1.02 |
| **IC75** | 4.59 | 6.07 | 3.882 | **IC75** | 4.59 | 15.69 | 1.15 |
| **MAX** | 100 | 2.35 | 4.463 | **MAX** | 100 | 100 | 4.89 |
| **Dose** | **Oxaliplatin µM** | **Lovastatin µM** | **CI Value** | **Dose** | **Oxaliplatin µM** | **Lovastatin µM** | **CI Value** |
| **IC25** | 1,53 | 1,40 | 0.796 | **IC25** | 1.53 | 5.23 | 1.84 |
| **IC50** | 3.06 | 2.80 | 0.837 | **IC50** | 3.25 | 10.46 | 1.19 |
| **IC75** | 4.59 | 4.20 | 1.012 | **IC75** | 4.59 | 15.69 | 1.5 |
| **MAX** | 100 | 100 | 23.251 | **MAX** | 100 | 100 | 17.6 |
| **Dose** | **Oxaliplatin µM** | **Fluvastatin µM** | **CI Value** | **Dose** | **Oxaliplatin µM** | **Fluvastatin µM** | **CI Value** |
| **IC25** | 1,53 | 1.75 | 0.325 | **IC25** | 1.53 | 3.64 | 1.23 |
| **IC50** | 3.06 | 3.45 | 0.159 | **IC50** | 3.25 | 7.28 | 4.20 |
| **IC75** | 4.59 | 5.20 | 0.507 | **IC75** | 4.59 | 10.92 | 4.45 |
| **MAX** | 100 | 100 | 2.799 | **MAX** | 100 | 100 | 25.2 |
| **MiaPaCa-2** | | | | **MiaPaCa-2 siRNA hERG1** | | | |
| **Dose** | **Gemcitabine**  **µM** | **Atorvastatin µM** | **CI Value** | **Dose** | **Gemcitabine**  **µM** | **Atorvastatin µM** | **CI Value** |
| **IC25** | 23.95 | 1.56 | 0.768 | **IC25** | 22.9 | 6.93 | 1.10 |
| **IC50** | 47.90 | 4.16 | 0.715 | **IC50** | 45.8 | 13.87 | 1.42 |
| **IC75** | 71.95 | 6.24 | 1.467 | **IC75** | 68.7 | 20.8 | 1.60 |
| **MAX** | 100 | 100 | 4.464 | **MAX** | 100 | 100 | 3.86 |
| **Dose** | **Gemcitabine**  **µM** | **Simvastatin**  **µM** | **CI Value** | **Dose** | **Gemcitabine µM** | **Simvastatin µM** | **CI Value** |
| **IC25** | 23.95 | 2.08 | 0.459 | **IC25** | 22.9 | 6.00 | 1.17 |
| **IC50** | 47.90 | 4.16 | 0.716 | **IC50** | 45.8 | 12.00 | 1.93 |
| **IC75** | 71.95 | 6.24 | 1.467 | **IC75** | 68.7 | 18.00 | 1.67 |
| **MAX** | 100 | 100 | 4.464 | **MAX** | 100 | 100 | 3.67 |
| **Dose** | **Gemcitabine µM** | **Lovastatin µM** | **CI Value** | **Dose** | **Gemcitabine µM** | **Lovastatin µM** | **CI Value** |
| **IC25** | 23.95 | 1.03 | 0.391 | **IC25** | 22.9 | 5.72 | 1.1 |
| **IC50** | 47.90 | 2.07 | 0.718 | **IC50** | 45.8 | 11.45 | 1.68 |
| **IC75** | 71.95 | 3.10 | 0.897 | **IC75** | 68.7 | 17.17 | 1.06 |
| **MAX** | 100 | 100 | 2.426 | **MAX** | 100 | 100 | 2.29 |
| **Dose** | **Gemcitabine µM** | **Fluvastatin µM** | **CI Value** | **Dose** | **Gemcitabine µM** | **Fluvastatin µM** | **CI Value** |
| **IC25** | 23.95 | 1.75 | 0.517 | **IC25** | 22.9 | 3.05 | 1.07 |
| **IC50** | 47.90 | 3.45 | 0.825 | **IC50** | 45.8 | 6.10 | 1.29 |
| **IC75** | 71.95 | 5.20 | 1.015 | **IC75** | 68.7 | 9.15 | 1.21 |
| **MAX** | 100 | 100 | 4.722 | **MAX** | 100 | **100** | 3.52 |
| **Dose** | **Oxaliplatin µM** | **Atorvastatin µM** | **CI Value** | **Dose** | **Oxaliplatin µM** | **Atorvastatin µM** | **CI Value** |
| **IC25** | 6.76 | 1.56 | 0.987 | **IC25** | 6.43 | 6.93 | 1.7 |
| **IC50** | 13.56 | 3.14 | 1.393 | **IC50** | 12.86 | 13.87 | 1.79 |
| **IC75** | 20.32 | 4.70 | 1.183 | **IC75** | 19.29 | 20.8 | 1.58 |
| **MAX** | 100 | 100 | 9.870 | **MAX** | 100 | 100 | 6.22 |
| **Dose** | **Oxaliplatin µM** | **Simvastatin µM** | **CI Value** | **Dose** | **Oxaliplatin µM** | **Simvastatin µM** | **CI Value** |
| **IC25** | 6.76 | 2.08 | 0.954 | **IC25** | 6.43 | 6.00 | 1.12 |
| **IC50** | 13.56 | 4.16 | 1.176 | **IC50** | 12.86 | 12.00 | 1.69 |
| **IC75** | 20.32 | 6.24 | 1.463 | **IC75** | 19.29 | 18.00 | 2.15 |
| **MAX** | 100 | 100 | 60.82 | **MAX** | 100 | 100 | 28.1 |
| **Dose** | **Oxaliplatin µM** | **Lovastatin µM** | **CI Value** | **Dose** | **Oxaliplatin µM** | **Lovastatin µM** | **CI Value** |
| **IC25** | 6.76 | 1.03 | 0.513 | **IC25** | 6.43 | 5.72 | 1.67 |
| **IC50** | 13.56 | 2.07 | 0.417 | **IC50** | 12.86 | 11.45 | 1.12 |
| **IC75** | 20.32 | 3.10 | 1.901 | **IC75** | 19.29 | 17.17 | 2.76 |
| **MAX** | 100 | 100 | 14.22 | **MAX** | 100 | 100 | 8.8 |
| **Dose** | **Oxaliplatin µM** | **Fluvastatin µM** | **CI Value** | **Dose** | **Oxaliplatin µM** | **Fluvastatin µM** | **CI Value** |
| **IC25** | 6.76 | 1.75 | 0.415 | **IC25** | 6.43 | 3.05 | 1.5 |
| **IC50** | 13.56 | 3.45 | 0.828 | **IC50** | 12.86 | 6.10 | 1.9 |
| **IC75** | 20.32 | 5.20 | 3.203 | **IC75** | 19.29 | 9.15 | 3.9 |
| **MAX** | 100 | 100 | 15.84 | **MAX** | 100 | **100** | 72.2 |

**Table S1. Combination index (CI) between Gemcitabin and statins, and Oxaliplatin and statins in PANC-1 and MiaPaCa2 cells and in hERG1 silenced (siRNA hERG1) PANC-1 and MiaPaCa2 cells.** 25% inhibitory concentration (IC25) of drugs are reported. Data are means±s.e.m. of three independent experiments, each carried out in triplicate. CI values were calculated using the Calcusyn software Version 2 (Biosoft).
